# Supplementary material for: Deciphering the evolutionary origin of the enantioselectivity of short-chain dehydrogenases from plants toward 1-borneol
Source: Nat Commun. 2026 Aug 14;17:9858. doi: 10.1038/s41467-026-76435-1 (PMC13578766; doi:10.1038/s41467-026-76435-1)
Supplement: Supplementary file 1 [file 41467_2026_76435_MOESM1_ESM.pdf]

Supplementary Information for:

Deciphering the evolutionary origin of the enantioselectivity of short-chain dehydrogenases from plants toward 1-borneol

Jasmin Zuson[#1], Carl P.O. Helmer[#2], Bruno Di Geronimo[#3], Andrea M. Chánique[1], Katarína Kavčiaková[1], Rosa Teijeiro-Juiz[2], Sebastian Brickel[4], Nuria Ramirez Molina[5], Ivana Drienovská[6], Daniel Kracher[1,7], Eric A. Gaucher[5], Shina Caroline Lynn Kamerlin[3,8,9], Bernhard Loll*[2], Robert Kourist*[1,7]*

[1] Institute of Molecular Biotechnology, Graz University of Technology, Petersgasse 14, Graz 8010, Austria. [2] Institute of Chemistry and Biochemistry, Laboratory of Structural Biochemistry, Freie Universität Berlin, Takustraße 6, 14195 Berlin, Germany. [3] School of Chemistry and Biochemistry, Georgia Institute of Technology, 901 Atlantic Drive NW, Atlanta, Georgia 30332-0400, United States. [4] Department of Chemistry - BMC, Uppsala University, BMC Box 576, S-751 23 Uppsala, Sweden. [5] Department of Biology, Georgia State University, Atlanta, Georgia, 30303, USA. [6] UVA Amsterdam, Department of Chemistry and Pharmaceutical Sciences, Vrije Universiteit Amsterdam, De Boelelaan 1108, 1081 HZ Amsterdam, The Netherlands. [7] BioTechMed-Graz, Mozartgasse 12, 8010 Graz, Austria. [8] School of Chemical and Biomolecular Engineering, Georgia Institute of Technology, 311 Ferst Dr., Atlanta, Georgia 30332-0400, United States. [9] Department of Chemistry, Lund University, Box 124, 22100 Lund, Sweden

[#]Equally contributing authors

*Corresponding authors:

Robert Kourist, Email: kourist@tugraz.at

Shina Caroline Lynn Kamerlin, Email: skamerlin3@gatech.edu

Bernhard Loll, Email: loll@chemie.fu-berlin.de

Table of contents

Table of contents 2
Supplementary Discussion 3
Supplementary Figures 4
Sequence logo BDH 4
Phylogenetic tree of plant BDHs 5
(-)-borneol conversion of ancestral variants 6
Addition of DMSO 7
Apparent Michaelis-Menten kinetics 8
Apparent E-value 9
Structural Alignment 10
N6 AlphaFold prediction 11
Ancestral Reconstruction Node Probabilities 13
EVcouplings analysis 14
Peripheral mutations 15
Kinetic data of mutant variants 16
Docking and ML/MM simulations 17
SDS-PAGE 59
Structure determination 62
Supplementary Tables 63
Biochemical data ancestors 63
Apparent K_M-values 63
Kinetic values of ancestral mutant variants 63
Crystallographic data 65
Docking and ML/MM data 68
Structural comparisons 70
Isoborneol conversion 71
Supplementary Data 72
Sequences of ancestral proteins 72
Equations 74
Supplementary References 76
Source Data 77

Supplementary Discussion

Despite identical processing of the diffraction data for SrBDH N32 and SrBDH N39, and refinement of both structures in PHENIX using essentially the same parameters, noticeable differences were observed in several refinement and validation statistics (Supplementary Table 4). Most prominently, the B-factors and clashscores differ substantially between the two structures.

To investigate the origin of these discrepancies, we examined the crystallographic properties of both datasets more closely. *Sr*BDH N32 crystallized in the monoclinic space group $P2_1$, whereas *Sr*BDH N39 crystallized in the orthorhombic space group *I*222, indicating a higher degree of crystal symmetry in the latter. The increased clashscore of *Sr*BDH N32 is likely related not only to its slightly lower resolution, but primarily to the reduced symmetry and consequently lower number of crystal contacts. These differences in crystal packing may likewise account for the pronounced variation in the Wilson B-factors obtained from XDS.

Notably, the average B-factors of the refined models are consistent with the Wilson B-factors derived from the experimental diffraction data, arguing against over-refinement of either structure. Overall, the observed differences in refinement metrics are most plausibly explained by intrinsic differences in crystal properties, including crystal symmetry, molecular packing, thermal motion, and the presence or absence of low-molecular-weight compounds. Therefore, although both structures were determined at comparable resolution, a direct comparison of their refinement statistics is not straightforward.

Supplementary Figures

Sequence logo BDH

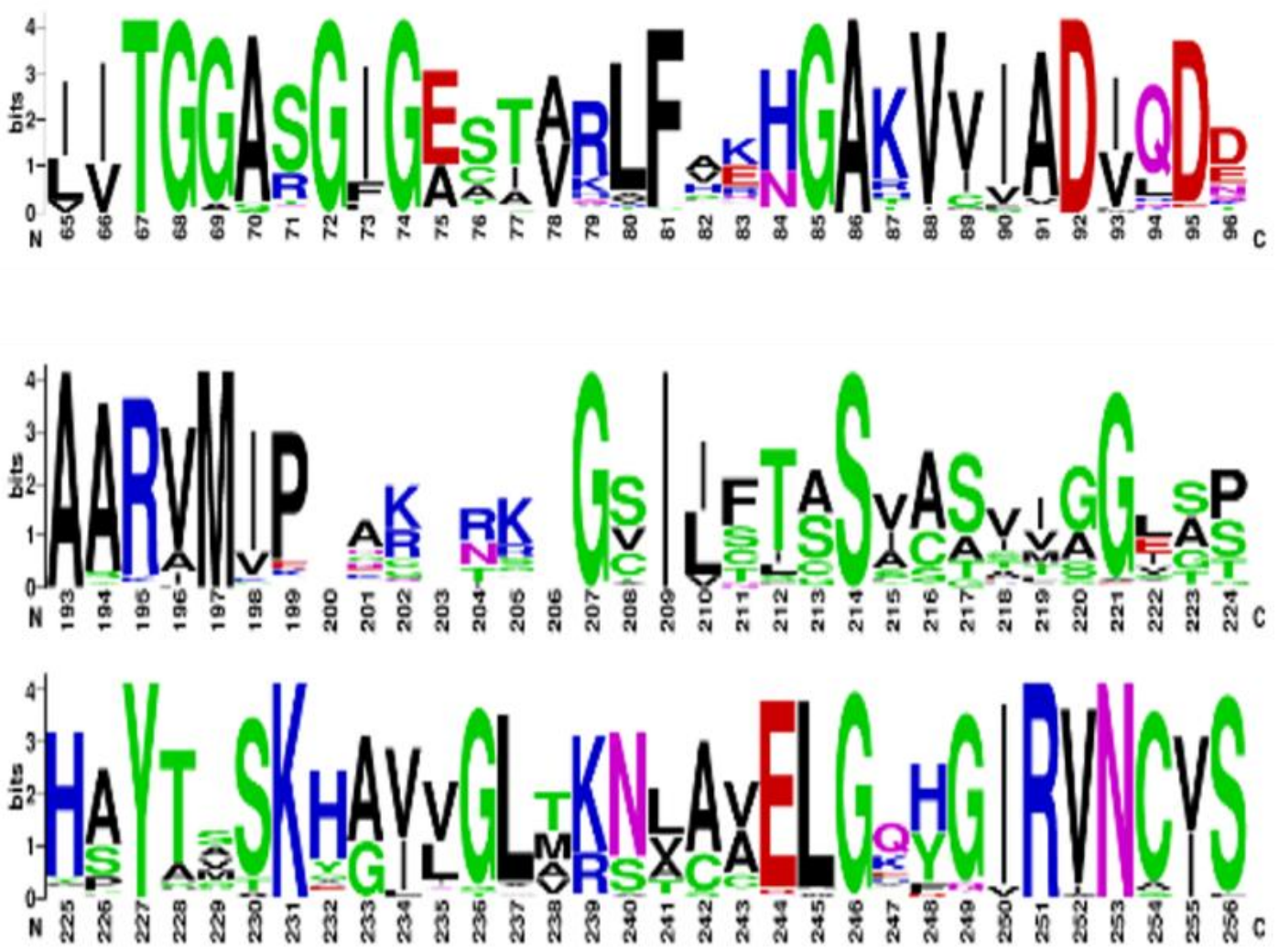

Supplementary Fig. 1. Graphic representation of the conserved motifs between the extant enzymes. The motif (SxxxxxxxxxxxxYxxxK) can be identified as S214, Y227 and K231 in the consensus sequence. The motif (TGxxx[AG]xG) can be identified between positions 67 and 74.

Phylogenetic tree of plant BDHs

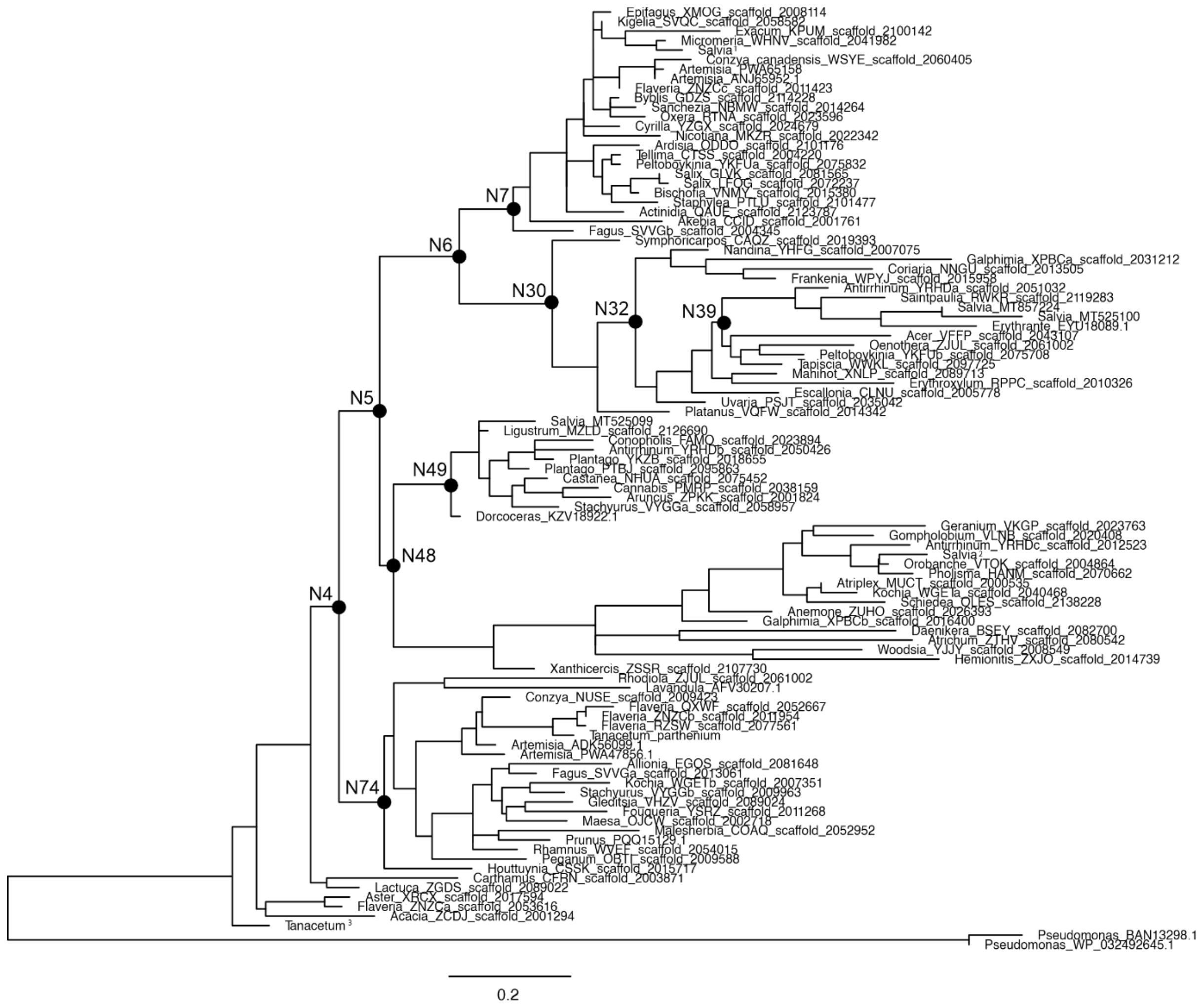

Supplementary Fig. 2. **Phylogenetic tree of plant BDHs.** The ten highlighted ancestors were characterized computationally or biochemically, or both. *Salvia* BDH sequences were reported by Chánique et. al. (2021)[1], *Tanacetum* BDHs are reported under https://db.cngb.org/onekp/species/Tanacetum%20parthenium; sample code = DUQG.

(-)-borneol conversion of ancestral variants

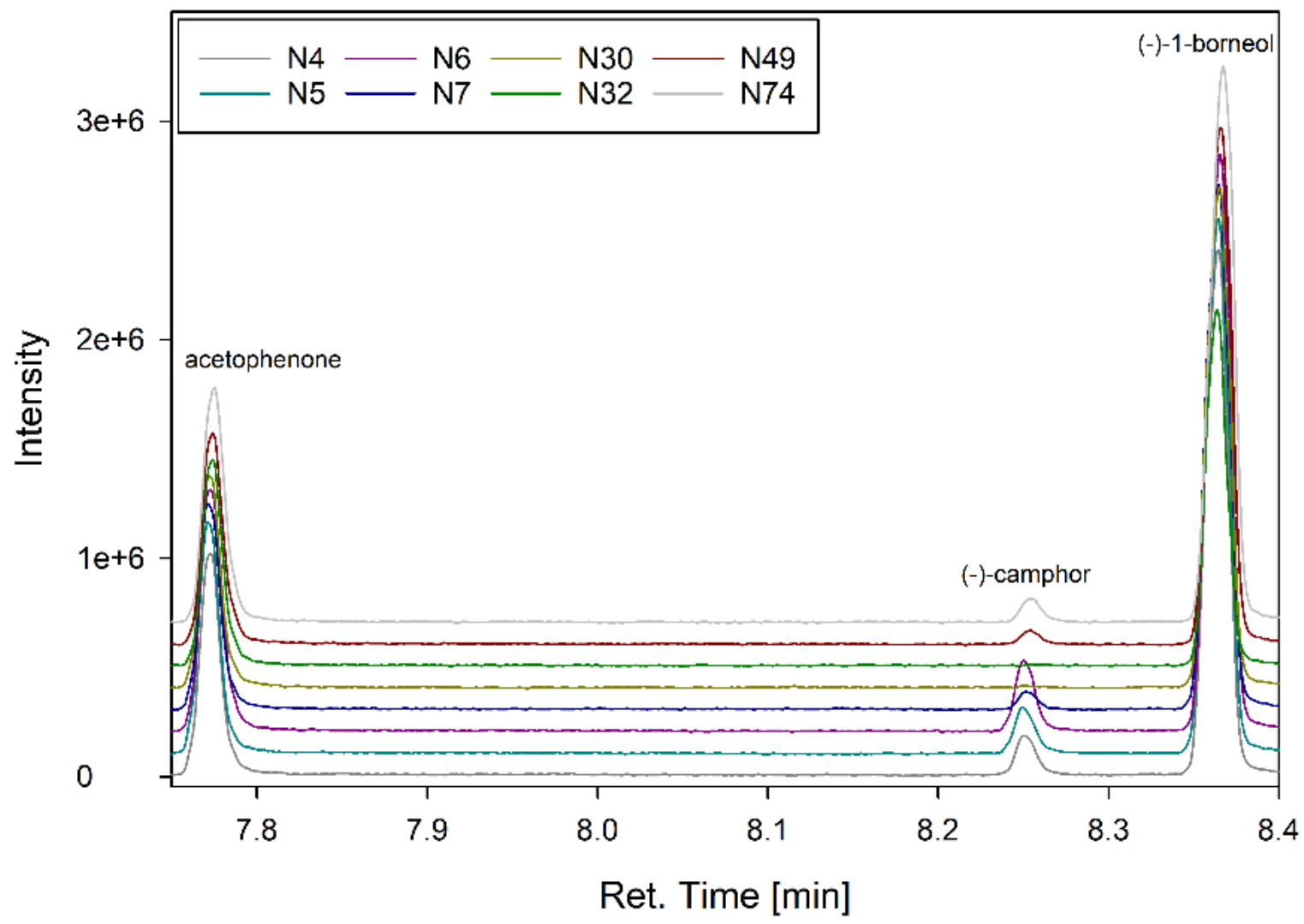

Supplementary Fig. 3. Endpoint measurements of BDH ancestors. Chromatogram of endpoint measurements (1.5 h) of (−)-1-borneol conversion with BDH ancestors N4, N5, N6, N7, N30, N32, N49 and N74 measured on GC-MS.

Addition of DMSO

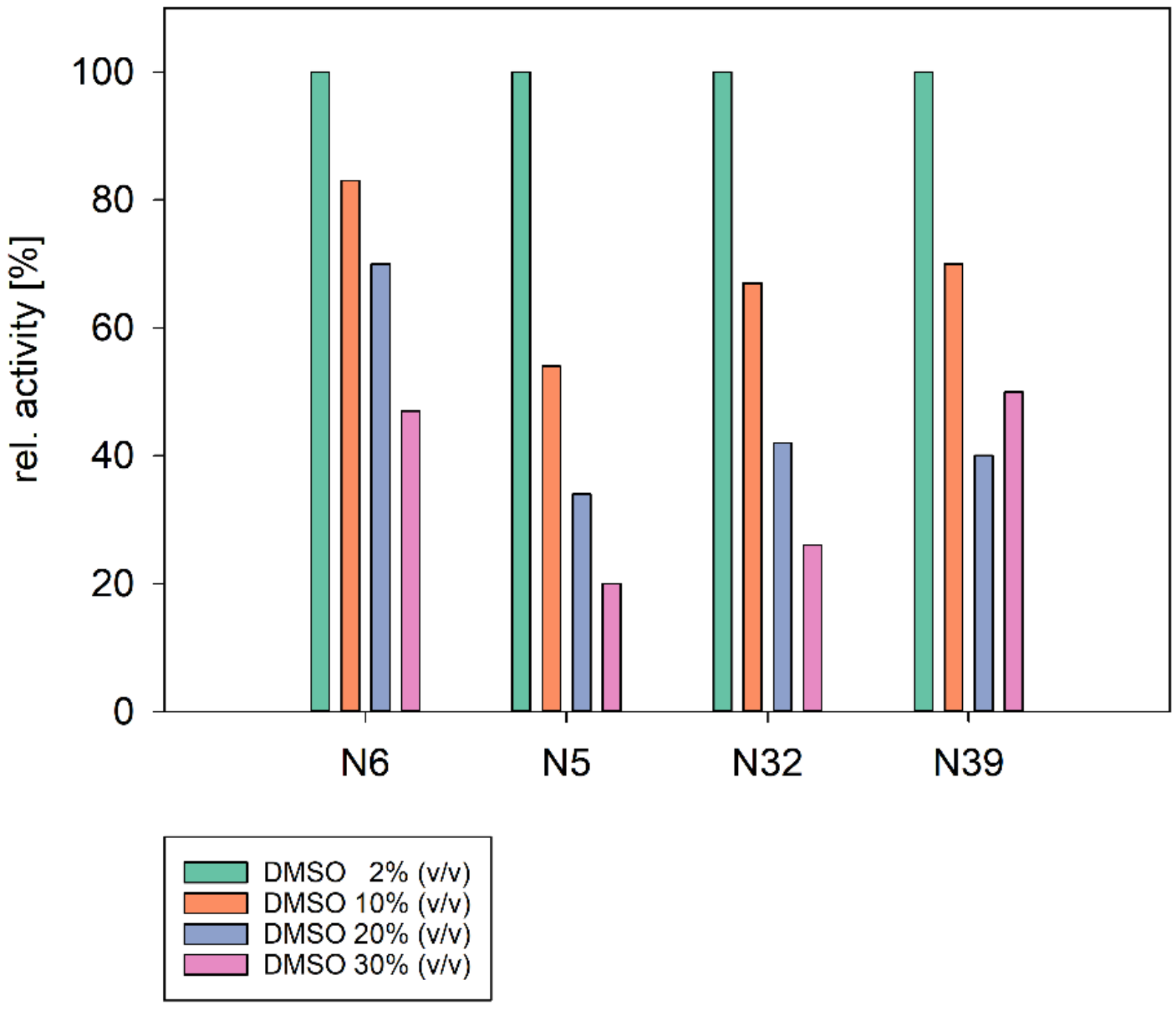

Supplementary Fig. 4. DMSO tolerance measurement. With (+)-1-borneol of ancestors N6, N5, N32, and N39 at 2%, 10%, 20% and 30% (v/v) DMSO. n=1.

Apparent Michaelis-Menten kinetics

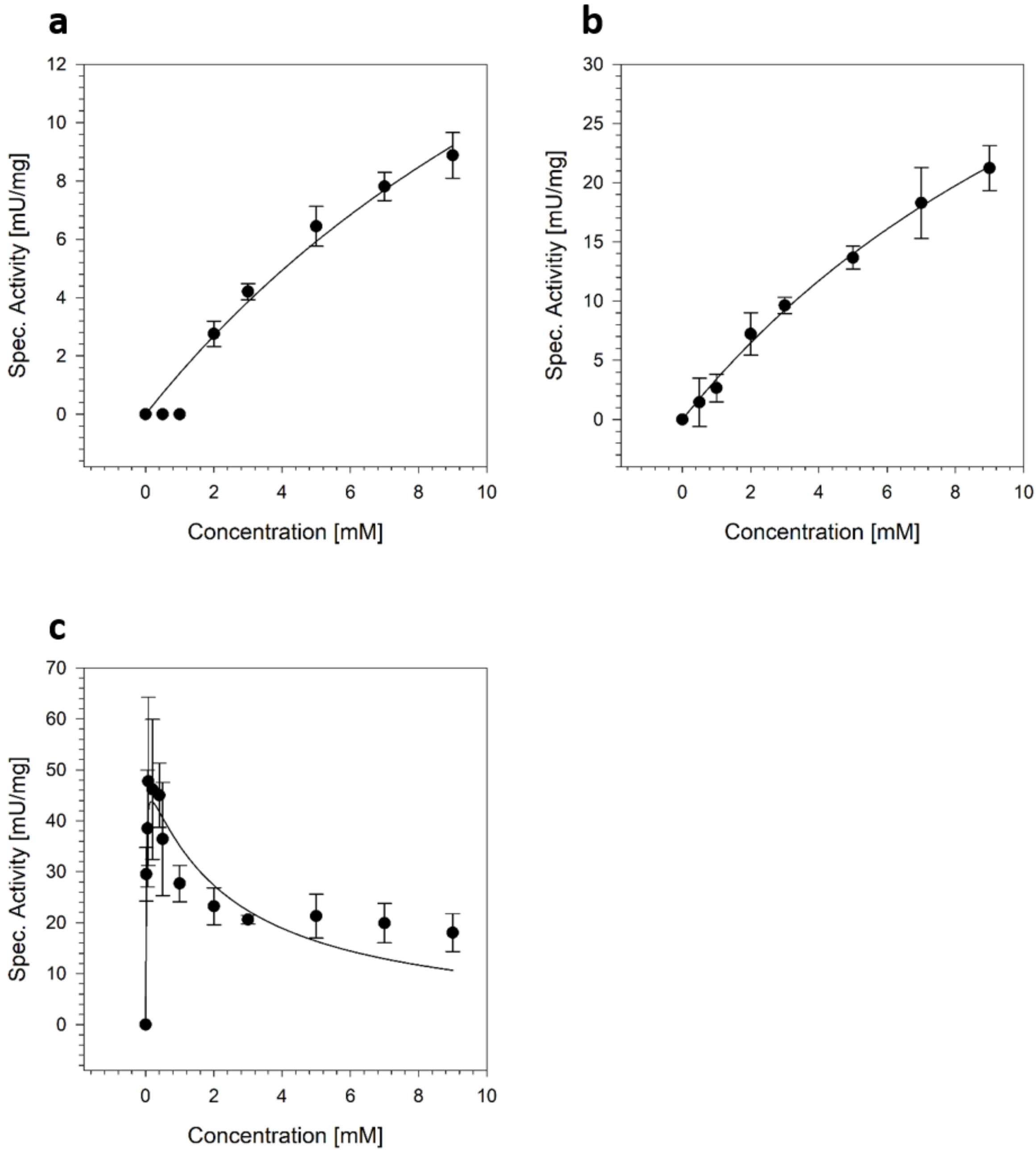

Supplementary Fig. 5. Determination of specific activities of the ancestors plotted against (+)-1-borneol concentration and Michaelis Menten regression visualized in Sigma Plot. Kinetic data represent variants: **a** N30, **b** N32, and **c** N6. Error bars represent the standard deviation of measurements carried out in quintuplicates (n=5). Raw data of the measurement is deposited under 10.5281/zenodo.18925996 (Raw data kinetics_N6_N30_N32.xlsx).

Apparent E-value

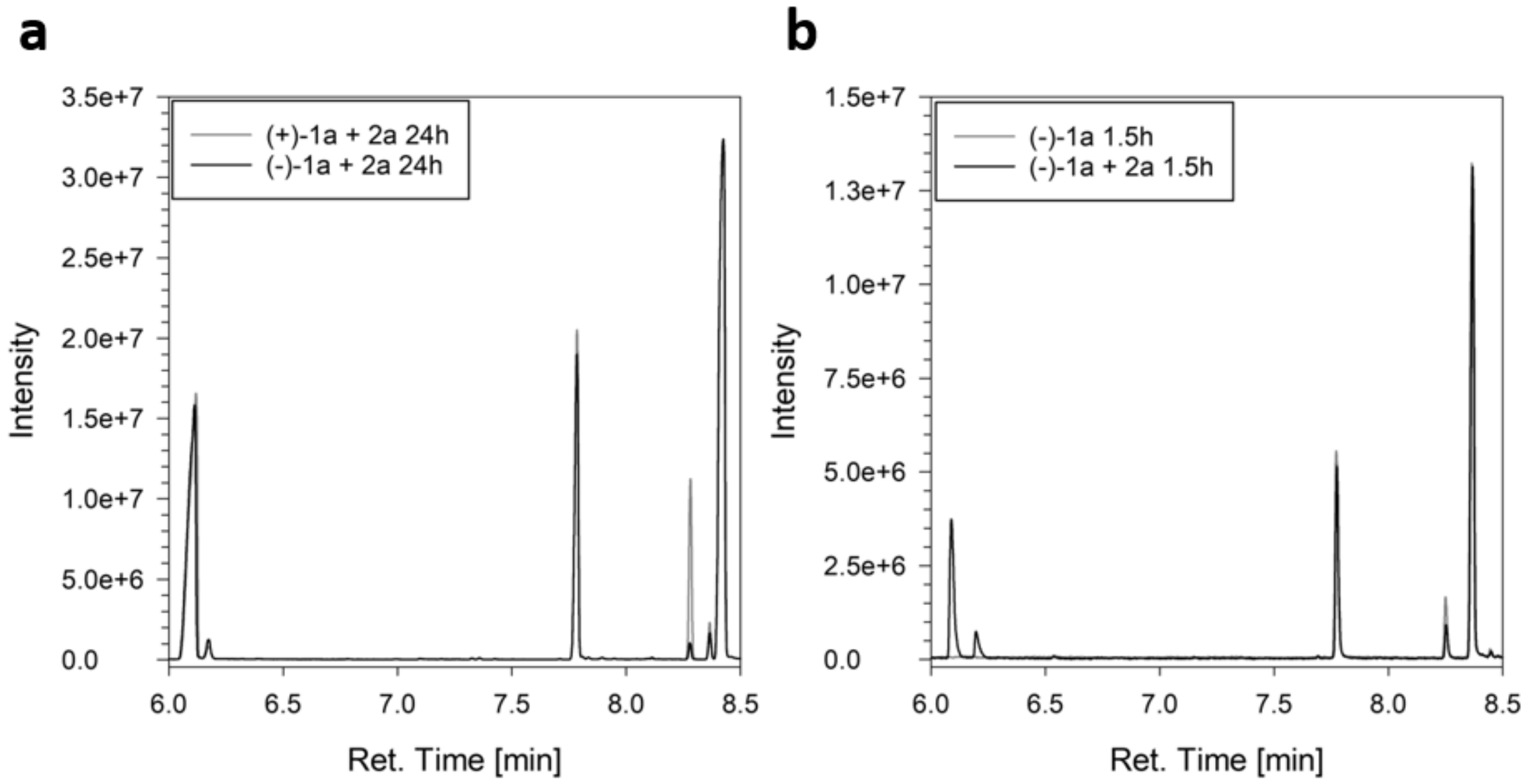

Supplementary Fig. 6. GC-MS chromatograms. GC-MS chromatograms highlighting the conversion of (+)-1-borneol ((+)-1a), (−)-1-borneol ((−)-1a) in comparison to the surrogate substrate cyclohexanol (2a). Retention (Ret.) times: cyclohexanol 6.06 min, cyclohexanone 6.17 min, acetophenone 7.79 min, camphor 8.29 min, isoborneol 8.35 min, and borneol 8.41 min. **a** Ancestor N6 under competitive conditions. **b** N6 under (non-)competitive conditions.

Structural Alignment

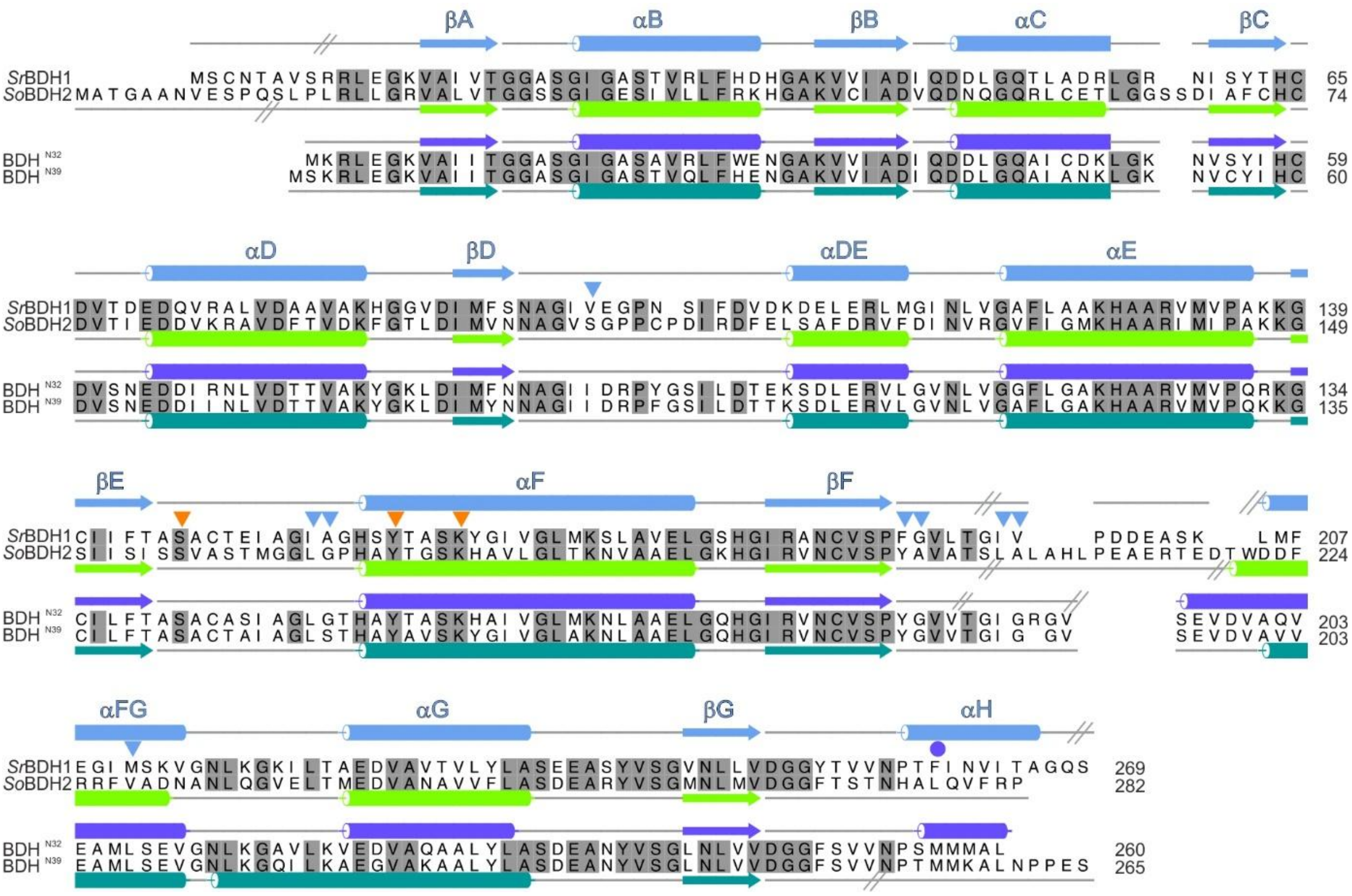

Supplementary Fig. 7. Structure-based sequence alignment of *Sr*BDH1 (GeneBank ID MT857224), *So*BDH2 (GeneBank ID MT525099), N32, and N39. Secondary structure elements are drawn above the alignment for *Sr*BDH1 and below for *So*BDH2 or N32 and below for N39 with α-helices depicted as cylinders and β-strands as arrows. Grey, inclined lines indicated sections of the structures, since they were not resolved in the reconstruction and could not be modelled. Orange triangles indicate the catalytic motif. Amino acids lining the putative active site of *Sr*BDH1, based on its crystal structure (PDB ID: 6ZYZ) with bound NAD^+, are indicated by blue triangles and as dark blue triangle if derived from another *Sr*BDH1 monomer within the tetramer. The dark green triangle marks a residue derived from another protomer of *So*BDH2 that completes the active site. Grey shaded amino acids are identical. The TGxxx[AG]xG NAD^+ binding motif, between βA and αB is indicated with a magenta rectangle.

N6 AlphaFold prediction

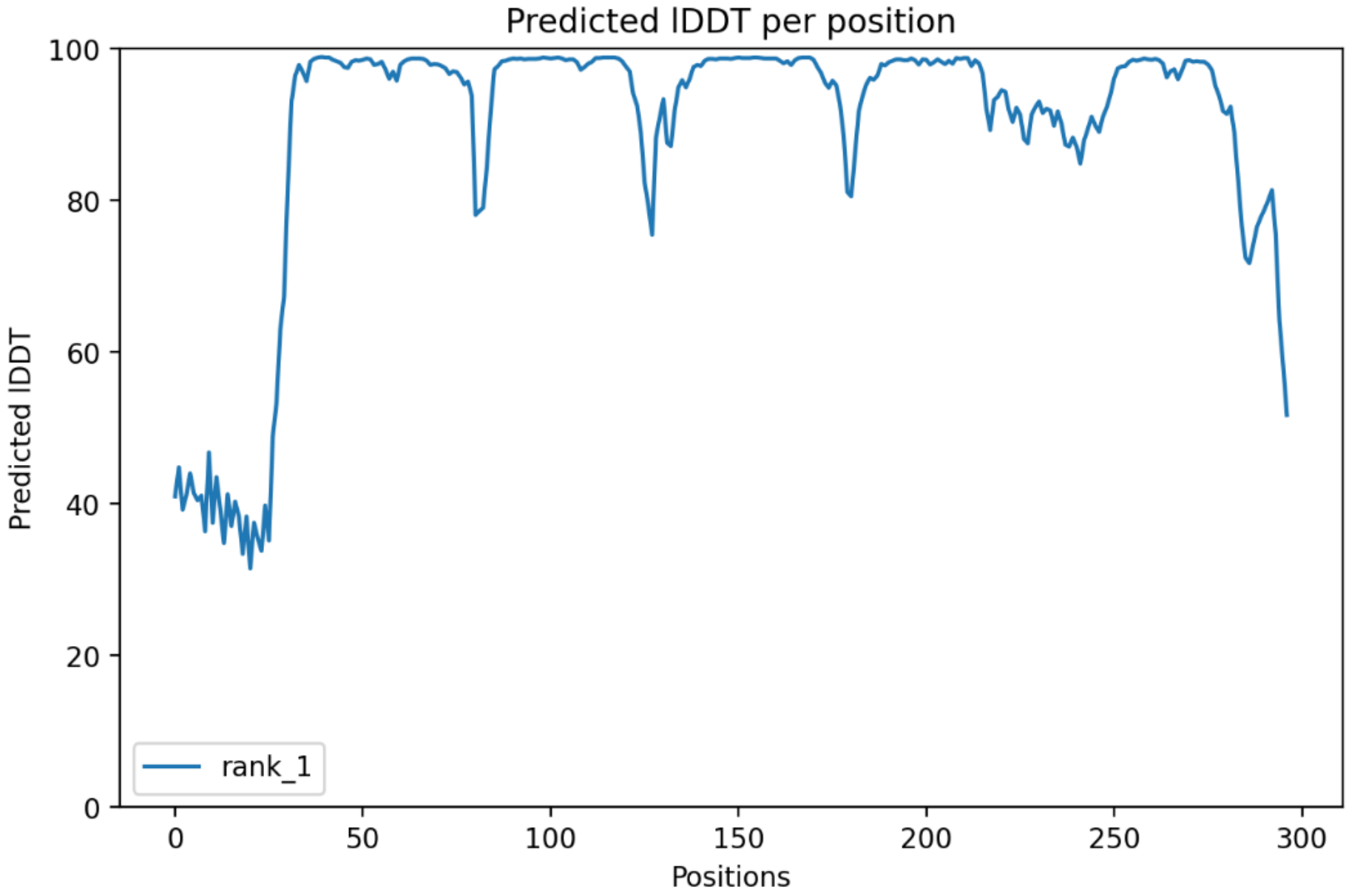

Supplementary Fig. 8. pLDDT score distribution. Model of N6 created with AlphaFold3.

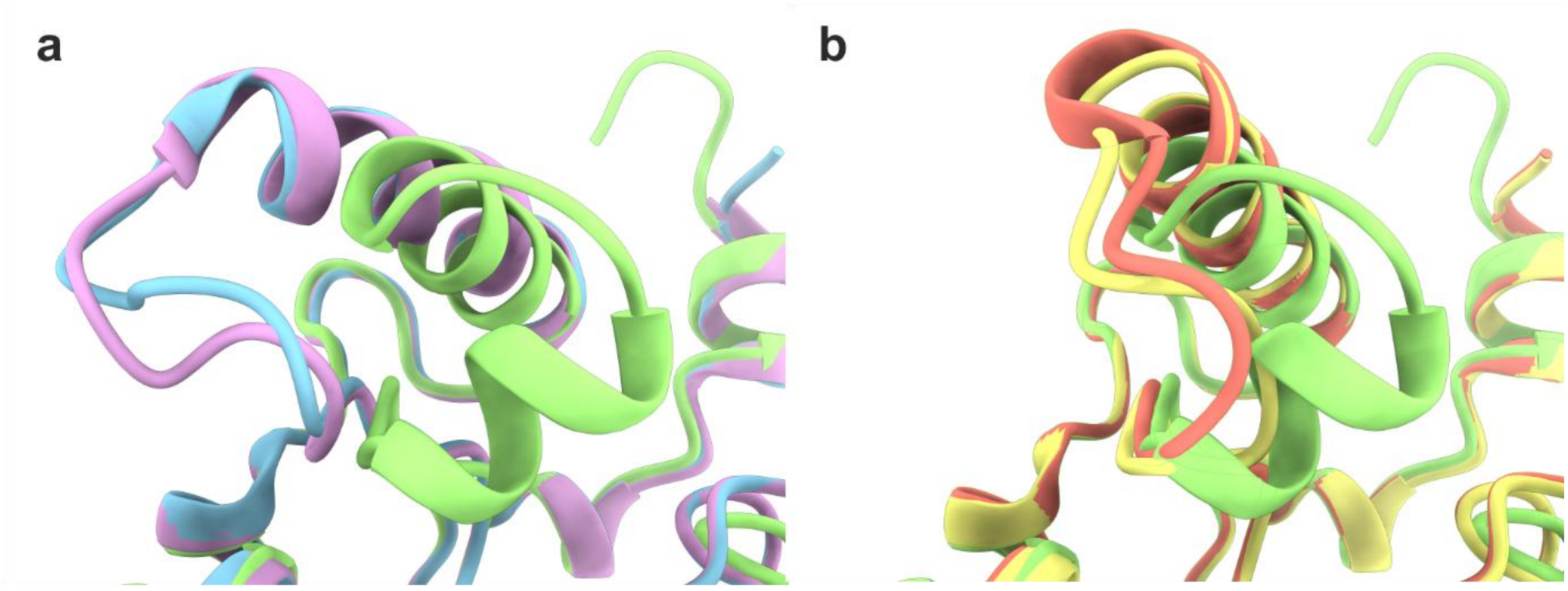

Supplementary Fig. 9. **Comparison of the loop-αFG motif structure in the N6 AlphaFold3 model and in the 4 chains of *Sr*BDH1 (PDB-ID: 6ZYZ). a** Chains A (blue) and B (lilac) show an open conformation of the loop, differing from the predicted N6 model. **b** Chains C (yellow) and D (salmon), while not completely aligning with the predicted model, show a closed conformation, closer to N6.

Ancestral Reconstruction Node Probabilities

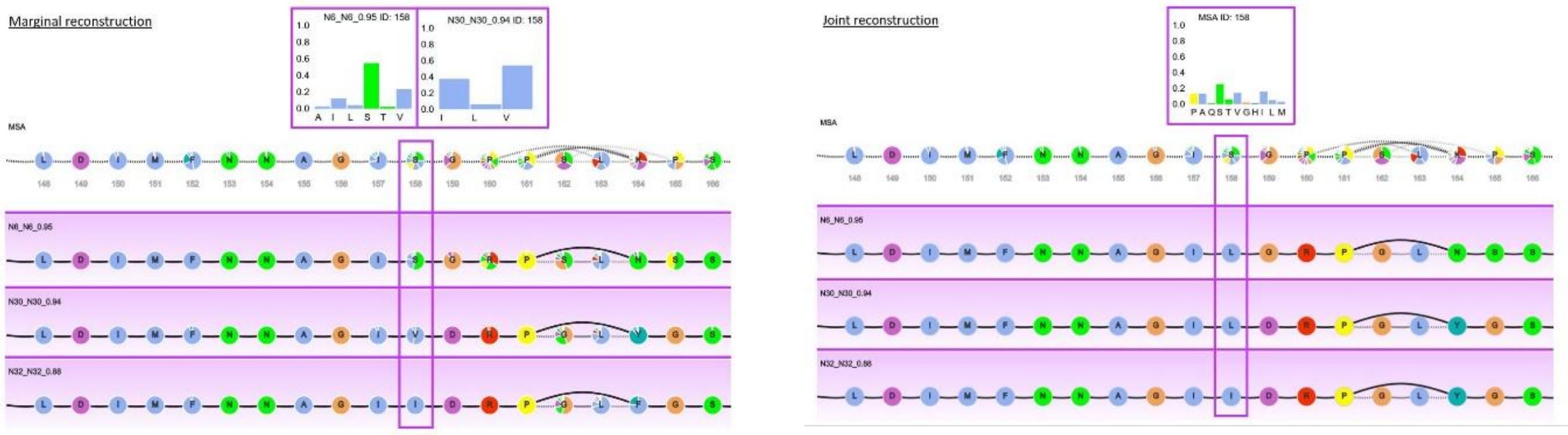

Supplementary Fig. 10. **Comparison of marginal and joint sequence reconstruction conducted on the GRASP webserver.** Statistics indicate the posterior probability of the residue corresponding to amino acid position 111 in N32 and N30. Visualization based on the GRASP web interface. Purple boxes highlight the reconstructed sequence of the ancestral nodes at the indicated positions. Dashed lines indicate the probable occurrence of gaps in the sequence.

EVcouplings analysis

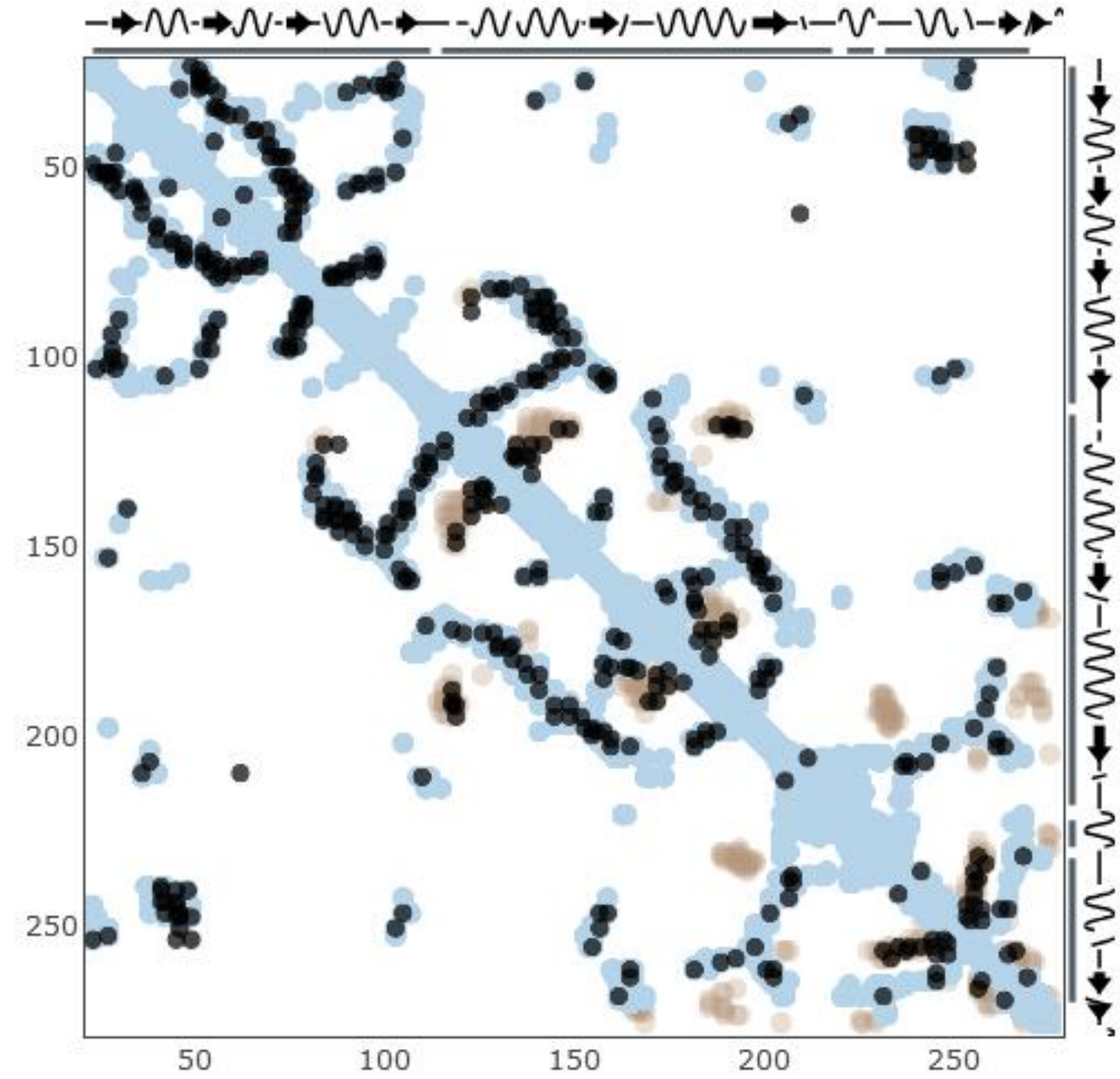

Supplementary Fig. 11. EVcouplings analysis. Distance map computed by EVcouplings based on N32. Black dots indicate evolutionary couples, light blue dots monomer contacts, and brown dots multimer contacts.

Peripheral mutations

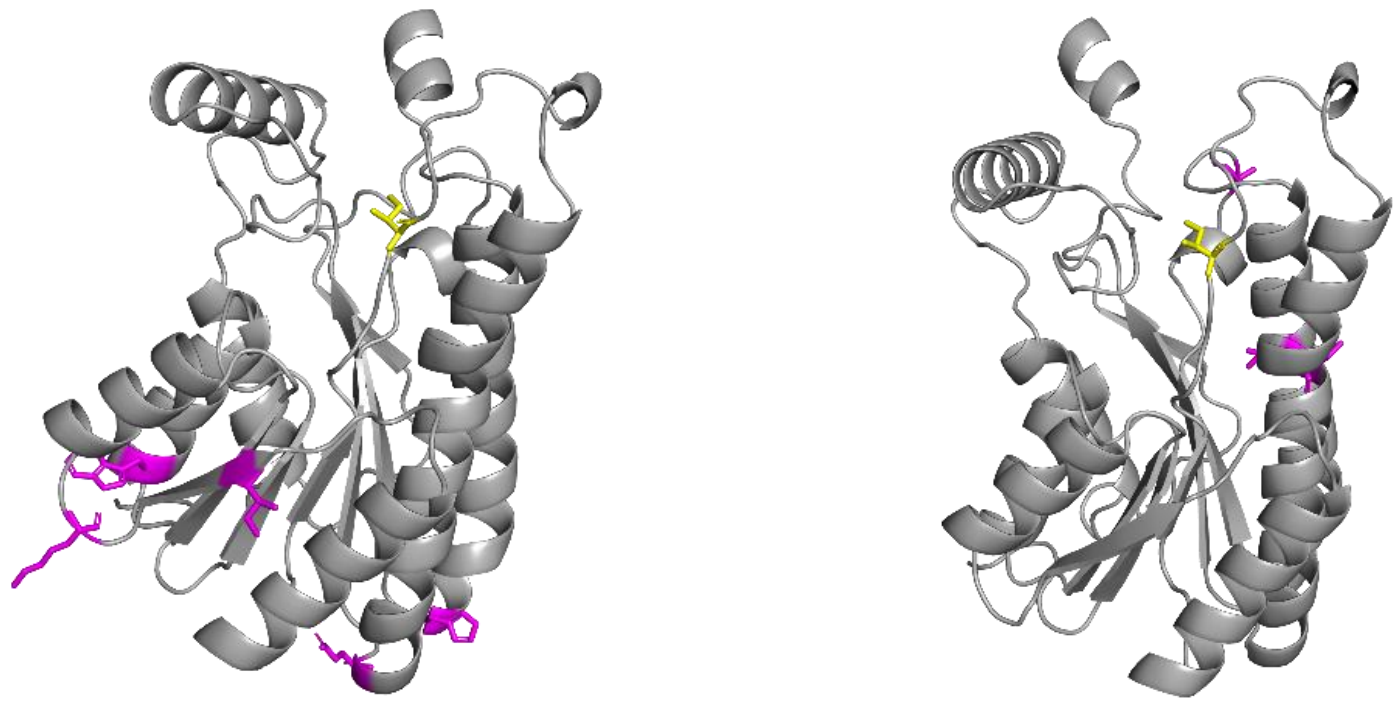

Supplementary Fig. 12. **AlphaFold3 models.** AlphaFold3 models with high-lighted mutations in the (left) *Sr*BDH N30_L111I/L47W/E74K/V79I/E107A/E153Q/Y197H variant and in the (right) *Sr*BDH N30_L111I/V136L/G169A/V183I variant. Yellow indicates position 111 as well as in purple indicates additional mutations.

Kinetic data of mutant variants

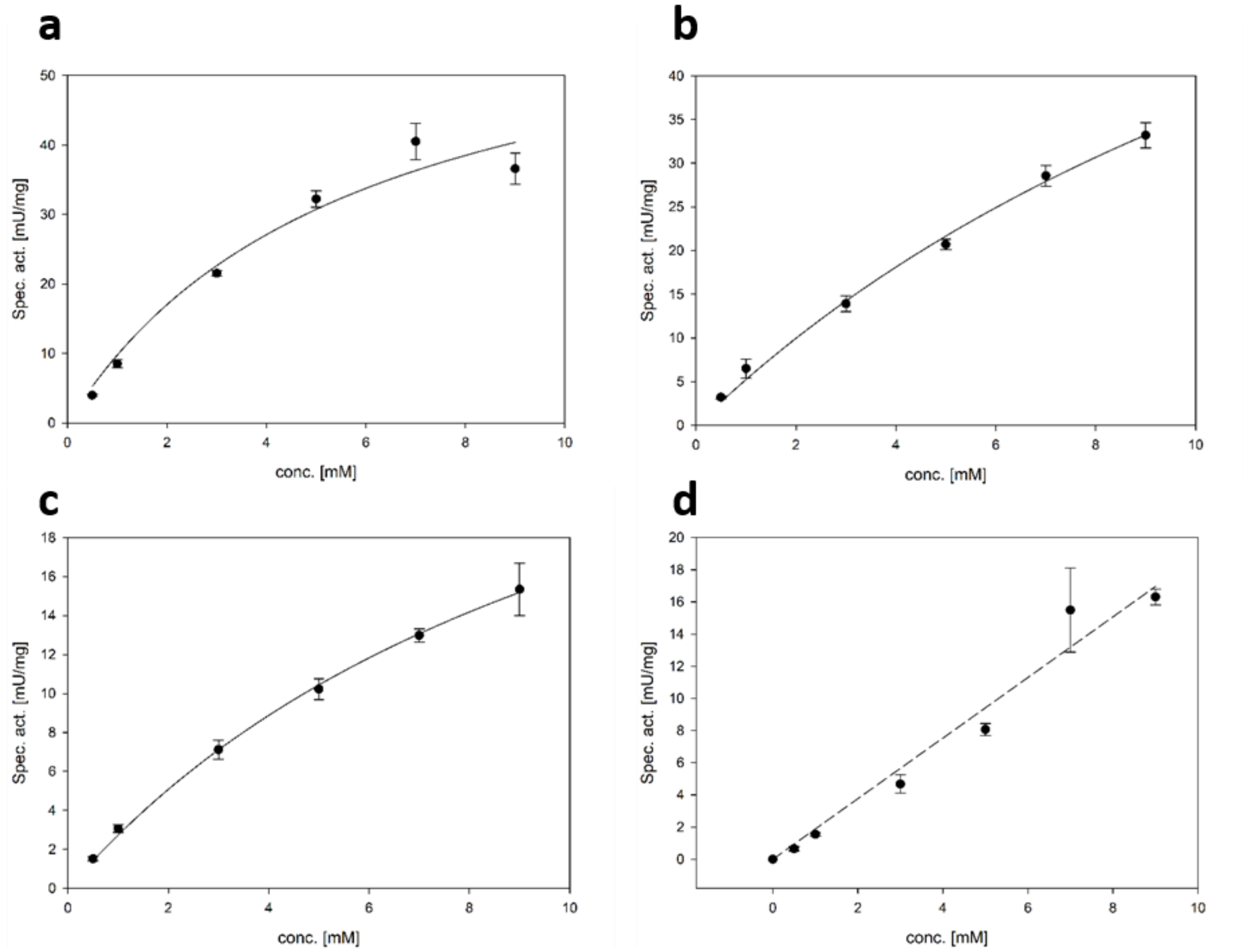

Supplementary Fig. 13. Kinetic data fitted to the Michaelis-Menten model. **a** N32_I111L, **b** N30_L111I **c** N30_L111I/G169A/V136L/V183I, and N30_L111I/V136L/G169A/V183I **d** Varying substrate conc. of (+)-borneol, 20% DMSO, 9 mM NAD+, 20 µM purified enzyme, 0.1 M Tris-HCl pH = 9. Measured on GC-FID equipped with a ZB-5 column. (n=3). Raw data and GC-chromatograms of the measurement are deposited under 10.5281/zenodo.18925996 (Chromatogramms.zip; N32_I111L_kin.xlsx; N30_L111IG169AV136LV183I_kin.xlsx; and N30_L111I_kin.xlsx;).

Docking and ML/MM simulations

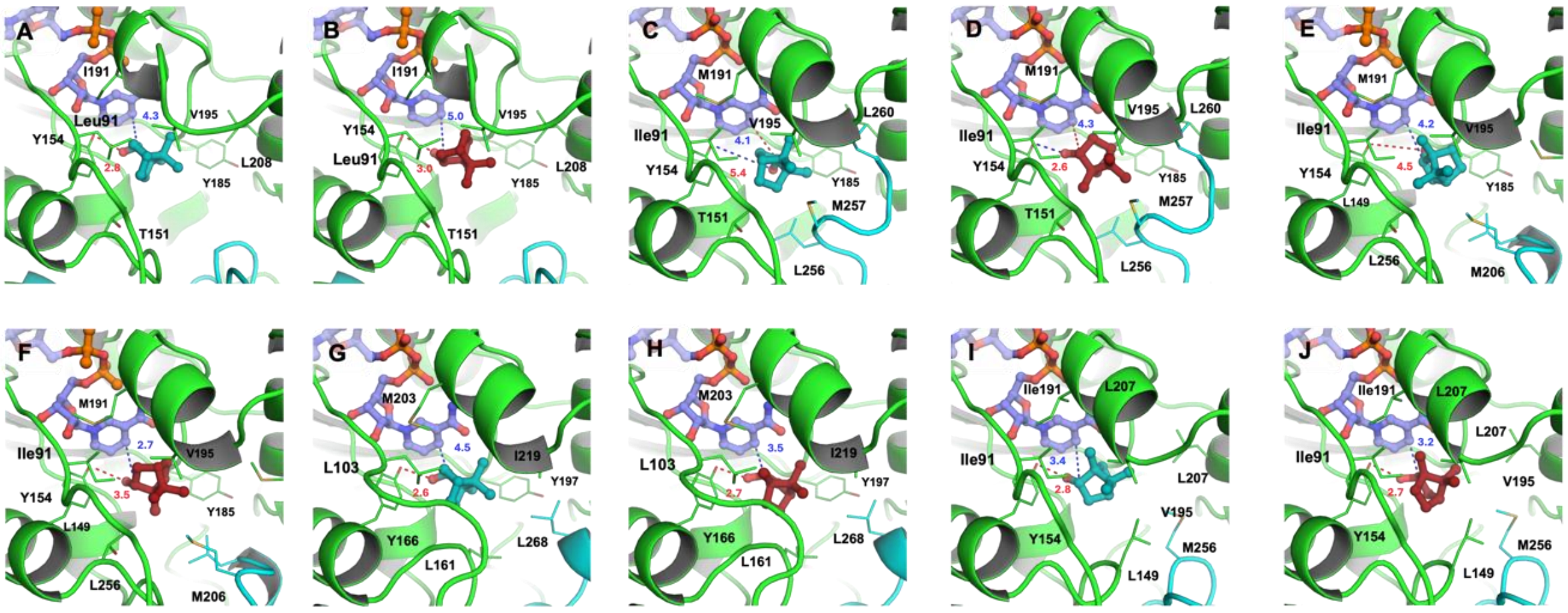

Supplementary Fig. 14. Selected docking poses. Selected docking poses for the complexes between: N32_I111L (**a-b**); N30_L111I (**c-d**); N30_L111I/G169A/V136L/V183I (**e-f**); N6 (**g-h**); N32 (**i-j**) with (+)-borneol is and (−)-borneol showed in teal and firebrick colour. Relevant amino acids in the binding are represented in sticks as well as NAD^+ coloured in slate.

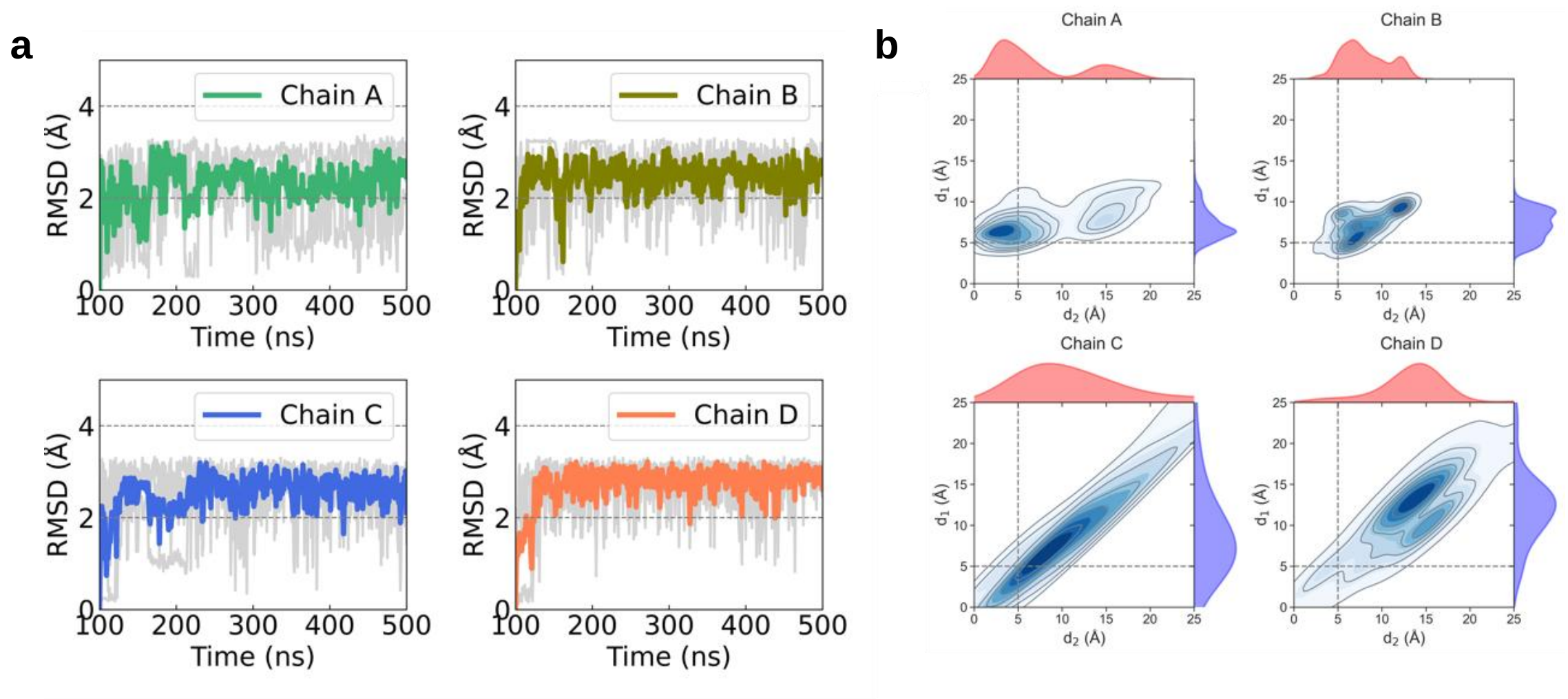

Supplementary Fig. 15. MD simulations of *Sr*BDH1 complex with (+)-borneol. a RMSD values (Å) from 3x400 ns MD simulations of *Sr*BDH1 complex with (+)-borneol. Individual replica trajectories are shown in gray, while the colored trace represents the mean RMSD (Å) averaged over the replicas. **b** Distributions of the two monitored catalytic distances (Å) across the simulation replicas; d_1 corresponds to the hydride transfer geometry between the C4 atom of the nicotinamide ring of NAD^+ and the oxidizable C5 carbon of borneol, and d_2 corresponds to the hydrogen-bond interaction between the borneol hydroxyl oxygen and the catalytic oxygen atom of the conserved tyrosine side chain.

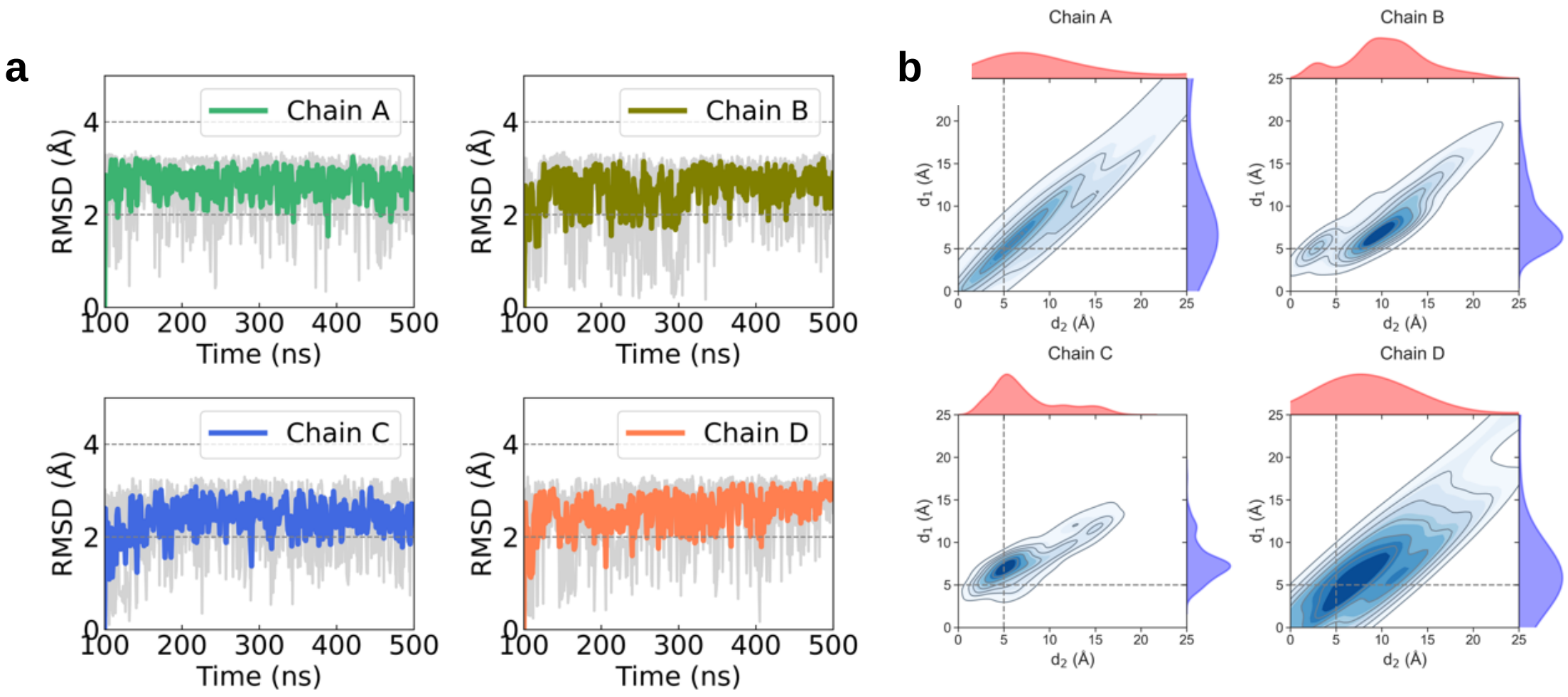

Supplementary Fig. 16. **a** RMSD values (Å) from 3x400 ns MD simulations of *Sr*BDH1 complex with (−)-borneol. Individual replica trajectories are shown in gray, while the colored trace represents the mean RMSD (Å) averaged over the replicas. **b** Distributions of the two monitored catalytic distances (Å) across the simulation replicas; d_1 corresponds to the hydride transfer geometry between the C4 atom of the nicotinamide ring of NAD^+ and the oxidizable C5 carbon of borneol, and d_2 corresponds to the hydrogen-bond interaction between the borneol hydroxyl oxygen and the catalytic oxygen atom of the conserved tyrosine side chain.

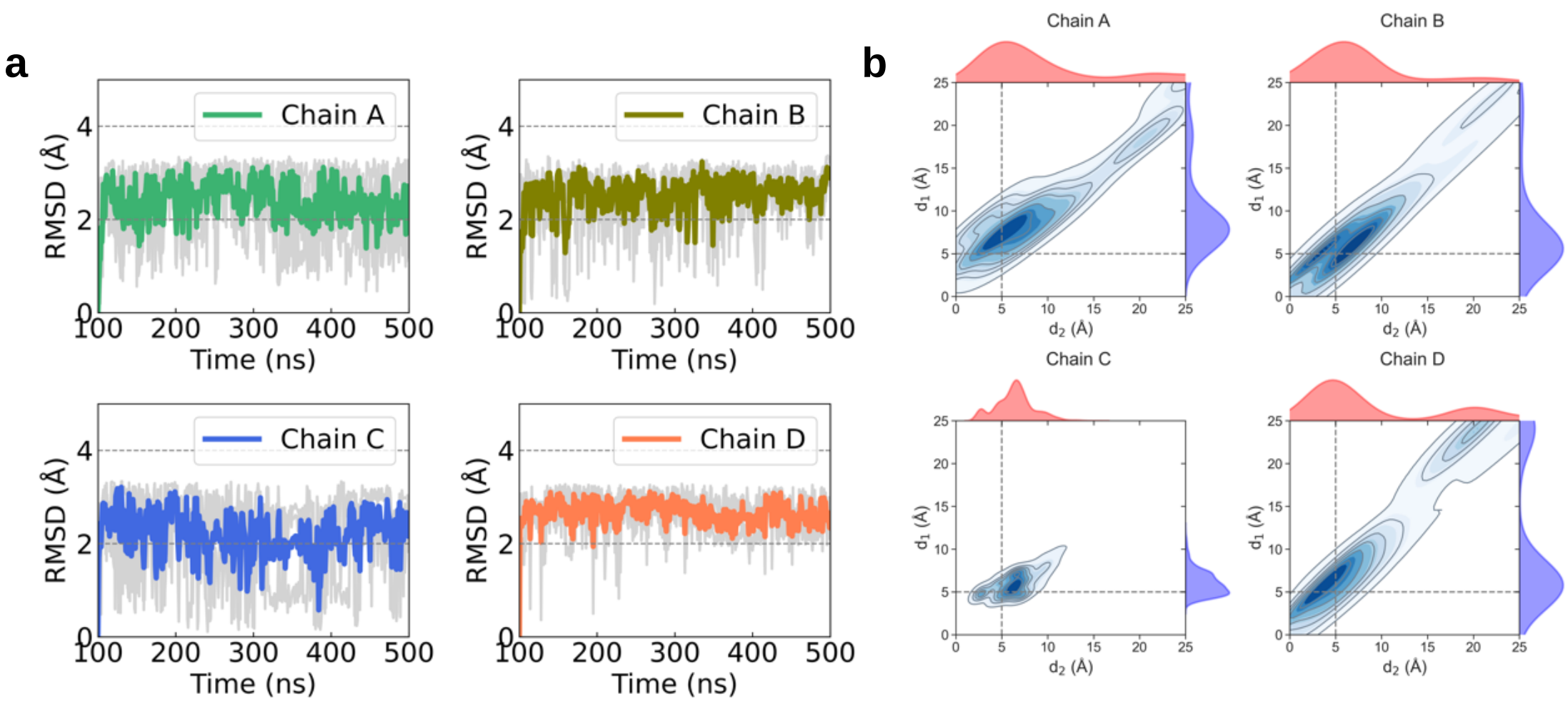

Supplementary Fig. 17. **a** RMSD values (Å) from 3x400 ns MD simulations of *Sr*BDH1 N32_I111L ancestor complex with (+)-borneol. Individual replica trajectories are shown in gray, while the colored trace represents the mean RMSD (Å) averaged over the replicas. **b** Distributions of the two monitored catalytic distances (Å) across the simulation replicas; d_1 corresponds to the hydride transfer geometry between the C4 atom of the nicotinamide ring of NAD^+ and the oxidizable C5 carbon of borneol, and d_2 corresponds to the hydrogen-bond interaction between the borneol hydroxyl oxygen and the catalytic oxygen atom of the conserved tyrosine side chain.

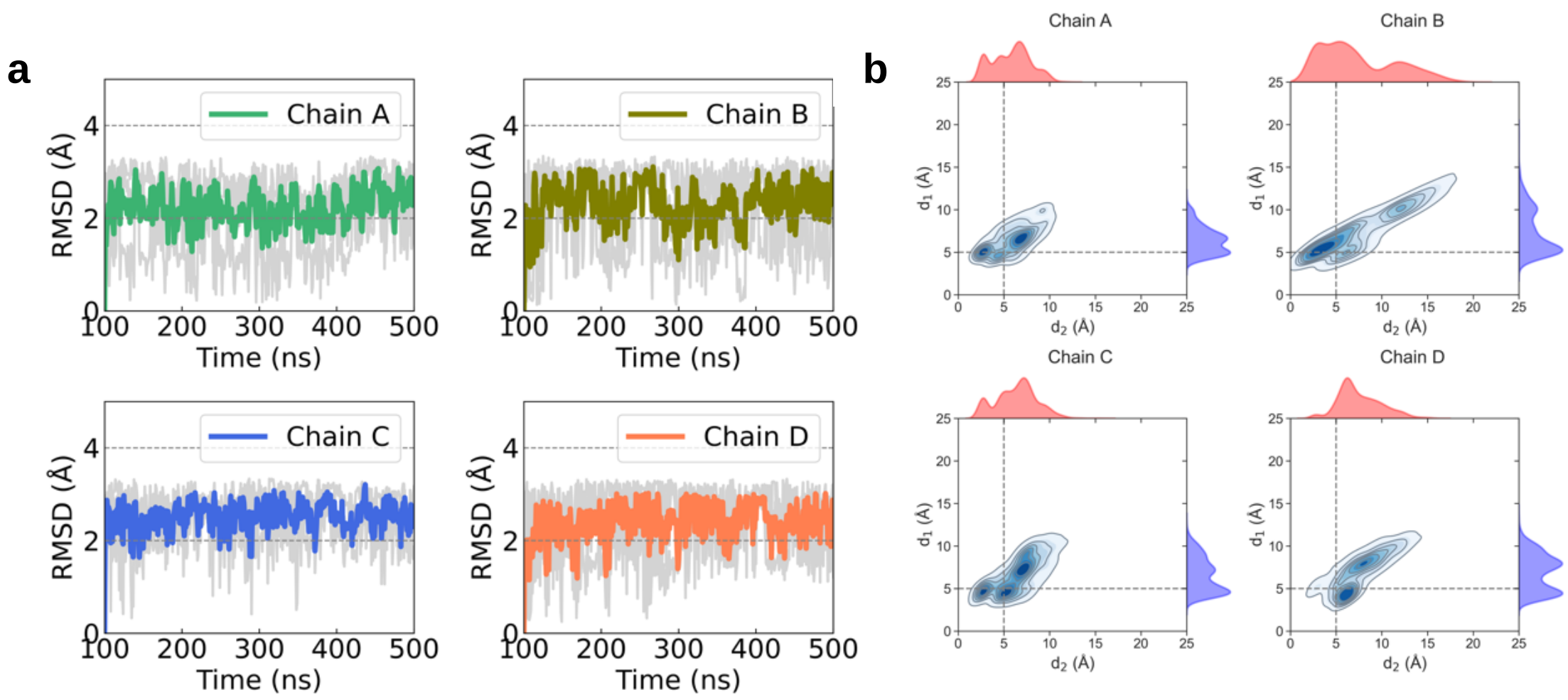

Supplementary Fig. 18. **a** RMSD values (Å) from 3x400 ns MD simulations of *Sr*BDH1 N32_I111L ancestor complex with (−)-borneol. Individual replica trajectories are shown in gray, while the colored trace represents the mean RMSD (Å) averaged over the replicas. **b** Distributions of the two monitored catalytic distances (Å) across the three simulation replicas; d_1 corresponds to the hydride transfer geometry between the C4 atom of the nicotinamide ring of NAD^+ and the oxidizable C5 carbon of borneol, and d_2 corresponds to the hydrogen-bond interaction between the borneol hydroxyl oxygen and the catalytic oxygen atom of the conserved tyrosine side chain.

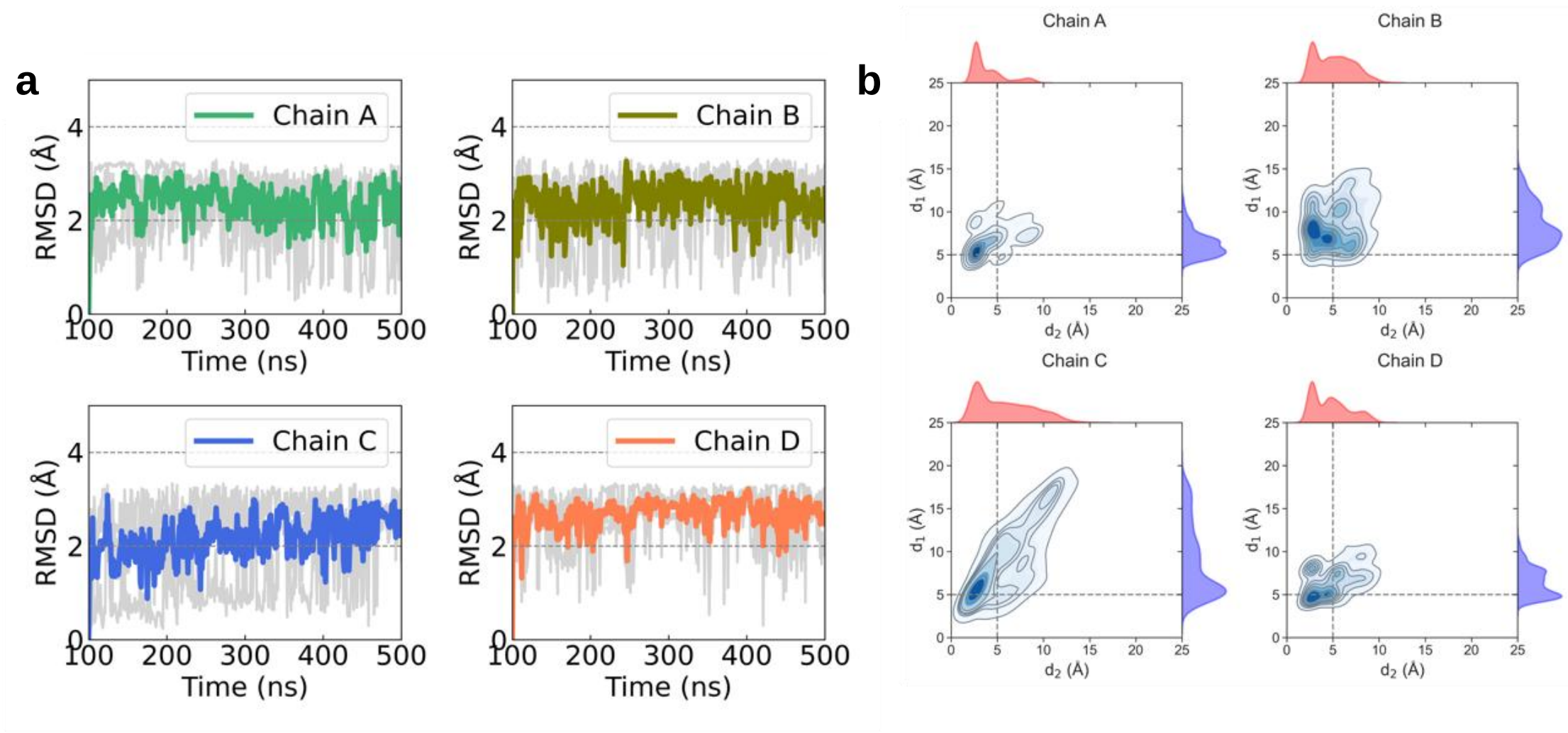

Supplementary Fig. 19. **a** RMSD values (Å) from 3x400 ns MD simulations of *Sr*BDH1 N30_L111I ancestor complex with (+)-borneol. Individual replica trajectories are shown in gray, while the colored trace represents the mean RMSD (Å) averaged over the replicas. **b** Distributions of the two monitored catalytic distances (Å) across the simulation replicas; d_1 corresponds to the hydride transfer geometry between the C4 atom of the nicotinamide ring of NAD^+ and the oxidizable C5 carbon of borneol, and d_2 corresponds to the hydrogen-bond interaction between the borneol hydroxyl oxygen and the catalytic oxygen atom of the conserved tyrosine side chain.

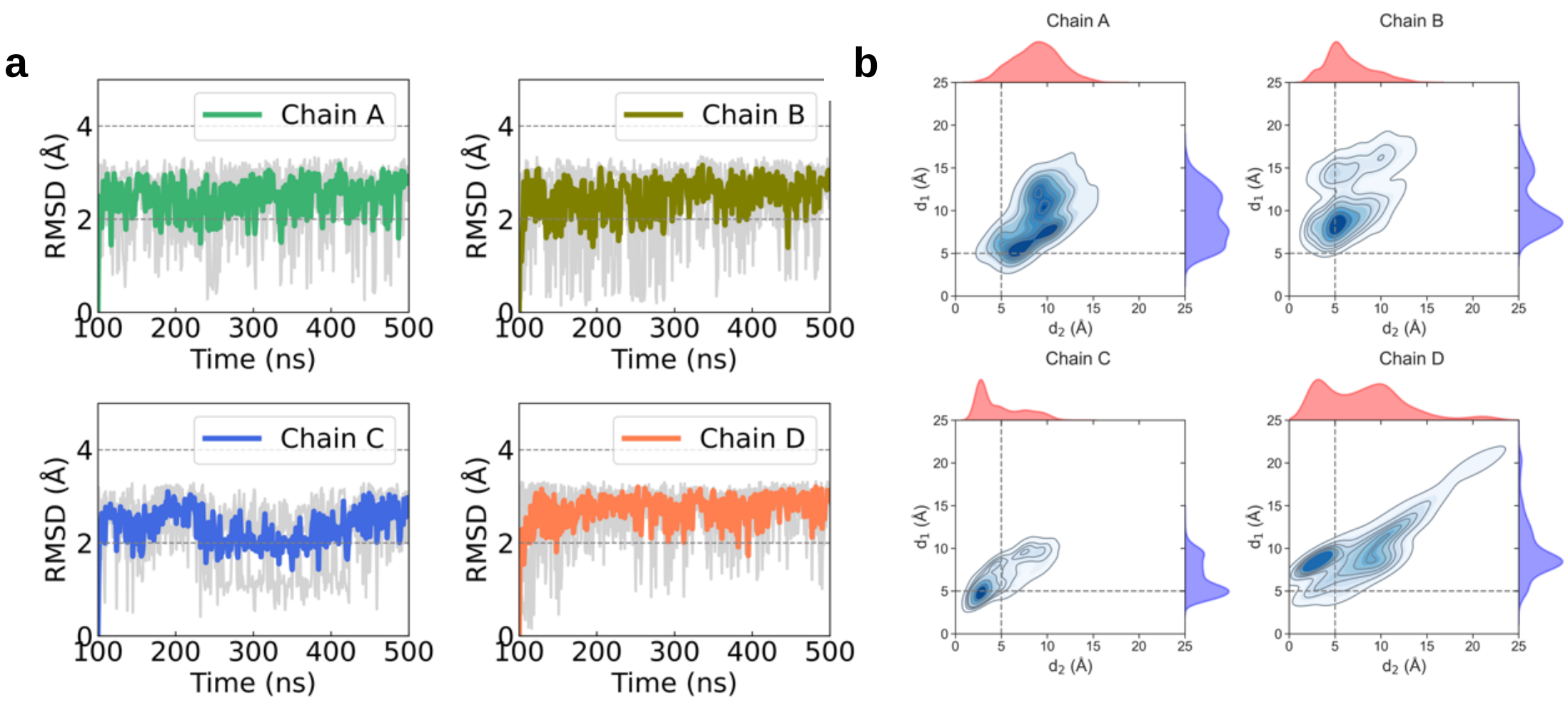

Supplementary Fig. 20. **a** RMSD values (Å) from 3x400 ns MD simulations of *Sr*BDH1 N30_L111I ancestor complex with (−)-borneol. Individual replica trajectories are shown in gray, while the colored trace represents the mean RMSD (Å) averaged over the replicas. **b** Distributions of the two monitored catalytic distances (Å) across the simulation replicas; d_1 corresponds to the hydride transfer geometry between the C4 atom of the nicotinamide ring of NAD^+ and the oxidizable C5 carbon of borneol, and d_2 corresponds to the hydrogen-bond interaction between the borneol hydroxyl oxygen and the catalytic oxygen atom of the conserved tyrosine side chain.

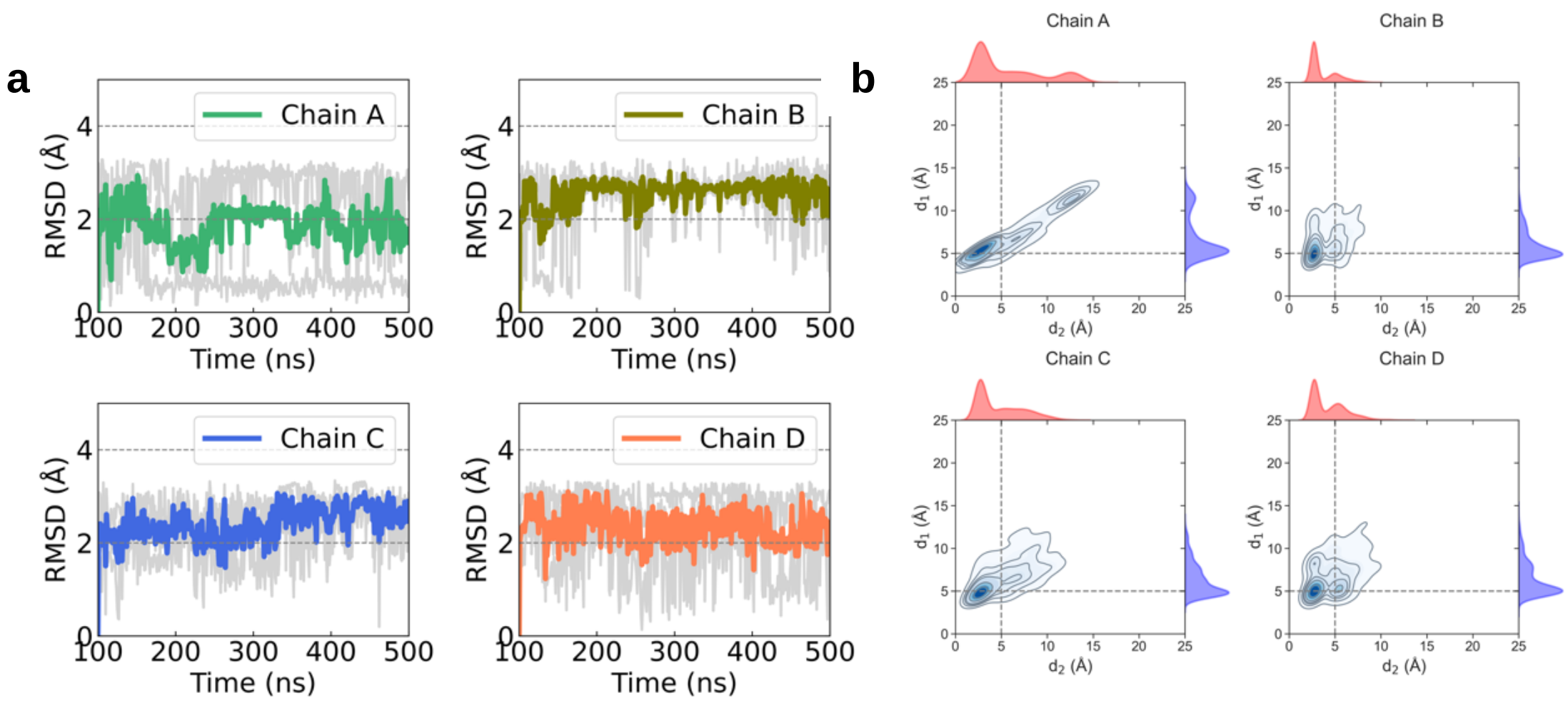

Supplementary Fig 21. **a** RMSD values (Å) from 3x400 ns MD simulations of *Sr*BDH1 N30_L111I/G169A/V136L/V183I ancestor complex with (+)-borneol. Individual replica trajectories are shown in gray, while the colored trace represents the mean RMSD (Å) averaged over the replicas. **b** Distributions of the two monitored catalytic distances (Å) across the simulation replicas; d_1 corresponds to the hydride transfer geometry between the C4 atom of the nicotinamide ring of NAD^+ and the oxidizable C5 carbon of borneol, and d_2 corresponds to the hydrogen-bond interaction between the borneol hydroxyl oxygen and the catalytic oxygen atom of the conserved tyrosine side chain.

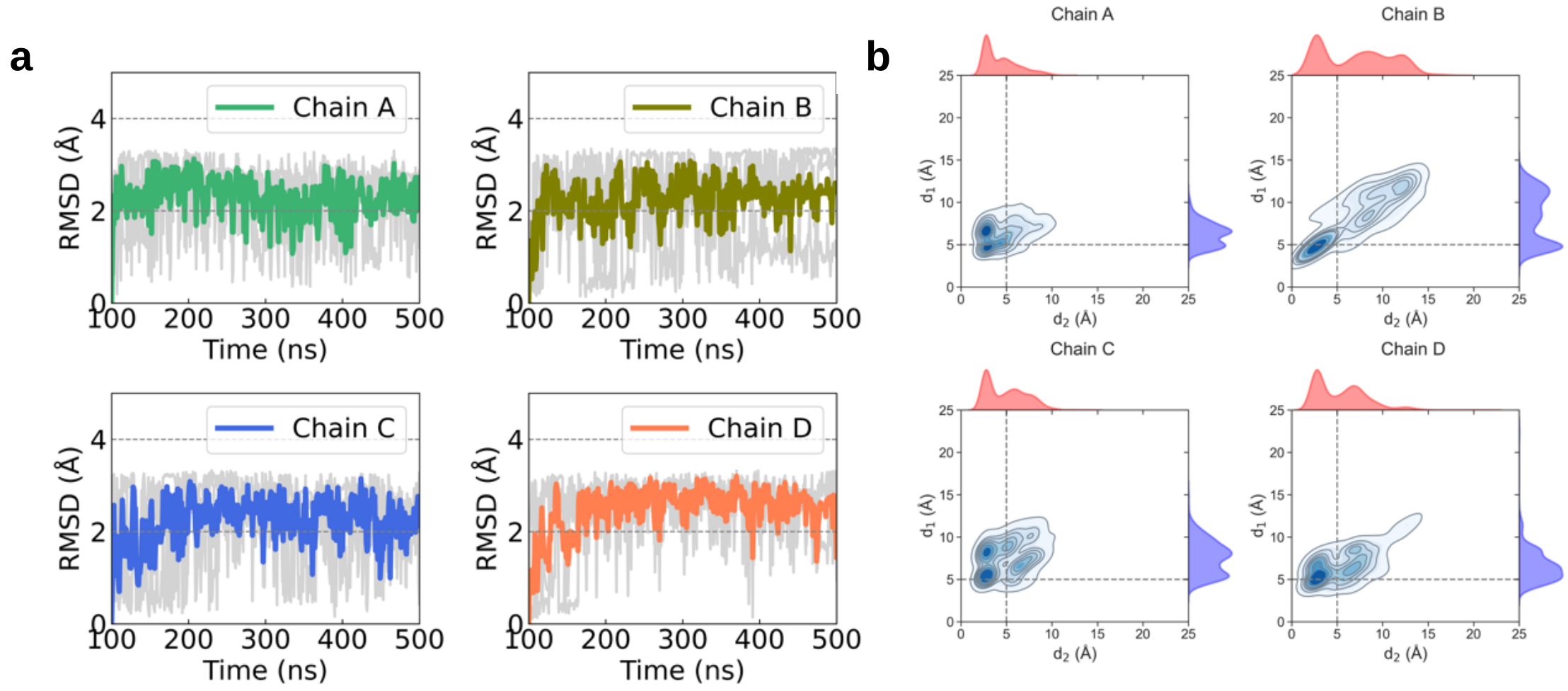

Supplementary Fig. 22. **a** RMSD values (Å) from 3x400 ns MD simulations of *Sr*BDH1 N30_L111I/G169A/V136L/V183I ancestor complex with (−)-borneol. Individual replica trajectories are shown in gray, while the colored trace represents the mean RMSD (Å) averaged over the replicas. **b** Distributions of the two monitored catalytic distances (Å) across the simulation replicas; d_1 corresponds to the hydride transfer geometry between the C4 atom of the nicotinamide ring of NAD^+ and the oxidizable C5 carbon of borneol, and d_2 corresponds to the hydrogen-bond interaction between the borneol hydroxyl oxygen and the catalytic oxygen atom of the conserved tyrosine side chain.

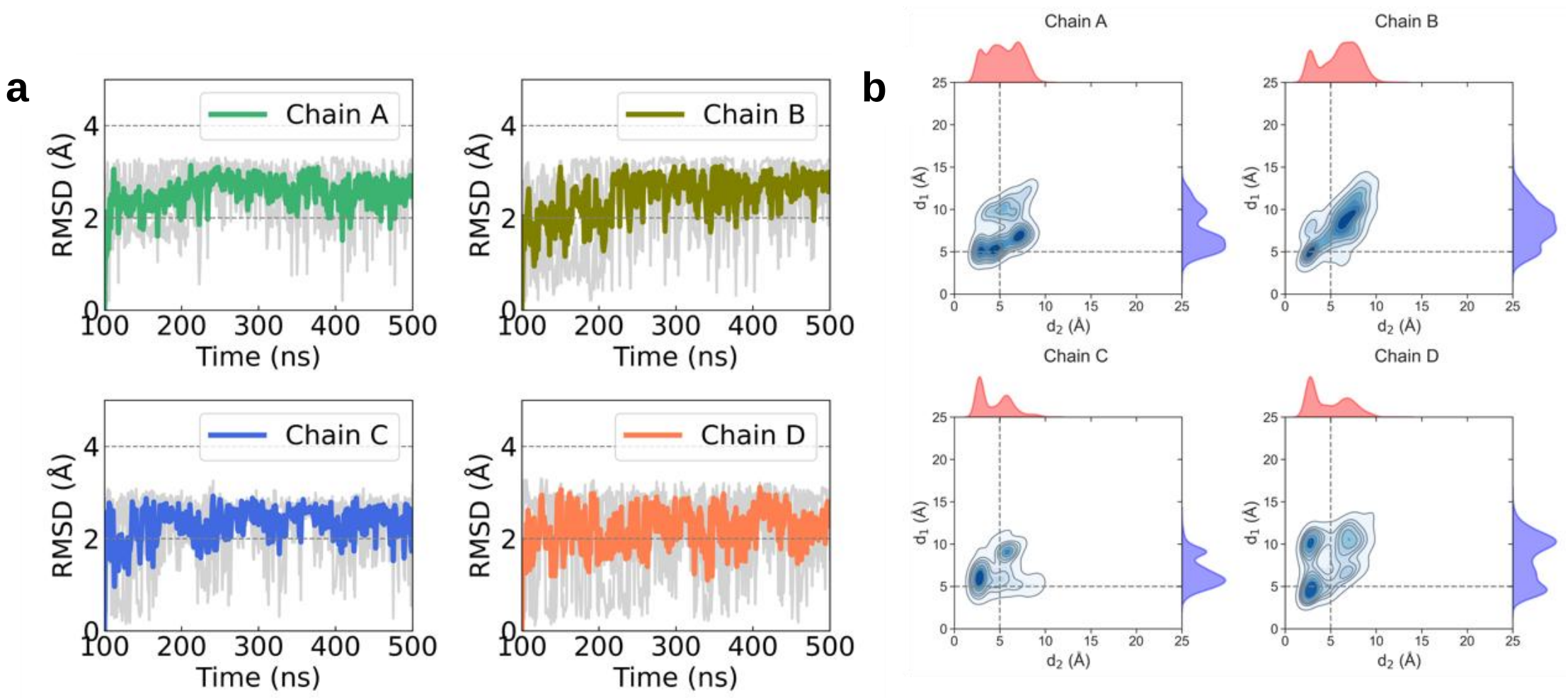

Supplementary Fig. 23. **a** RMSD values (Å) from 3x400 ns MD simulations of *Sr*BDH1 N6 ancestor complex with (+)-borneol. Individual replica trajectories are shown in gray, while the colored trace represents the mean RMSD (Å) averaged over the replicas. **b** Distributions of the two monitored catalytic distances (Å) across the simulation replicas; d_1 corresponds to the hydride transfer geometry between the C4 atom of the nicotinamide ring of NAD^+ and the oxidizable C5 carbon of borneol, and d_2 corresponds to the hydrogen-bond interaction between the borneol hydroxyl oxygen and the catalytic oxygen atom of the conserved tyrosine side chain.

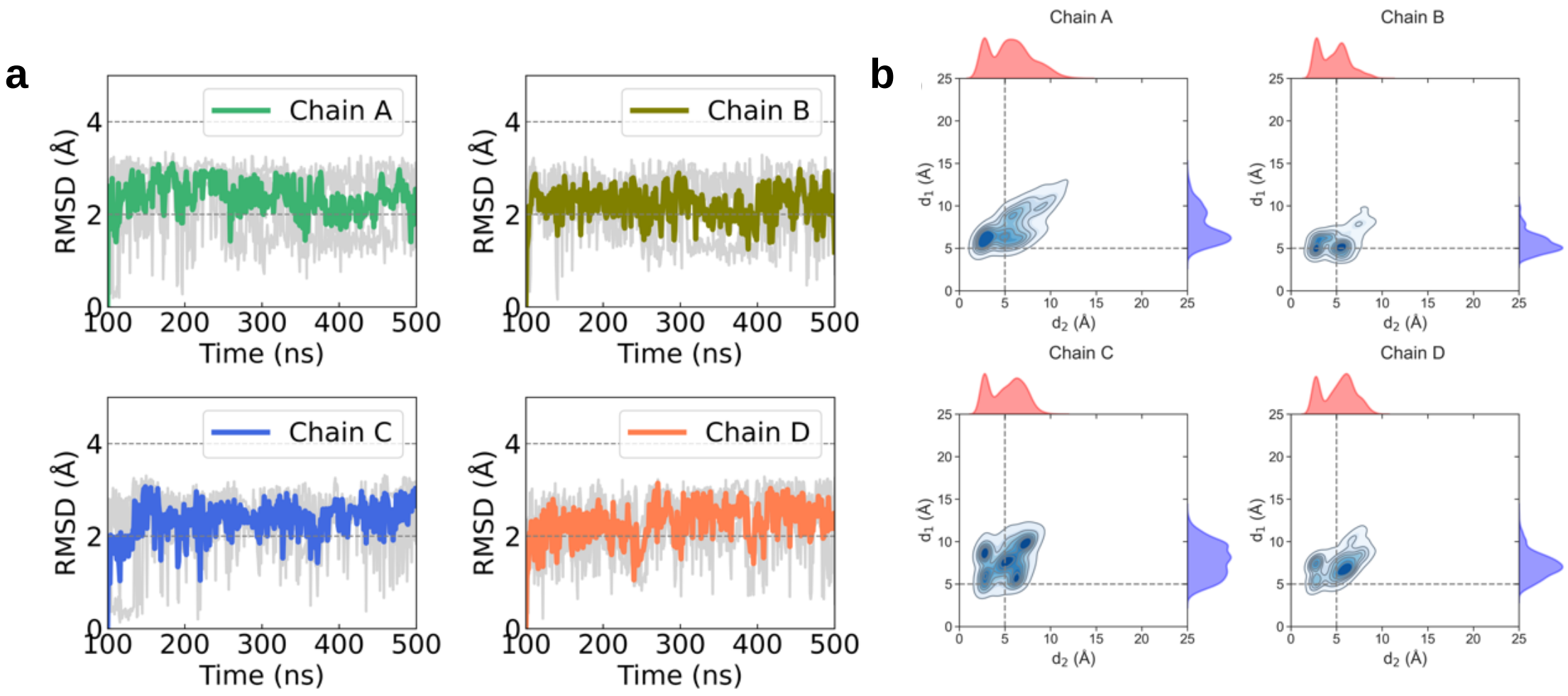

Supplementary Fig. 24. **a** RMSD values (Å) from 3x400 ns MD simulations of *Sr*BDH1 N6 ancestor complex with (−)-borneol. Individual replica trajectories are shown in gray, while the colored trace represents the mean RMSD (Å) averaged over the replicas. **b** Distributions of the two monitored catalytic distances (Å) across the simulation replicas; d_1 corresponds to the hydride transfer geometry between the C4 atom of the nicotinamide ring of NAD^+ and the oxidizable C5 carbon of borneol, and d_2 corresponds to the hydrogen-bond interaction between the borneol hydroxyl oxygen and the catalytic oxygen atom of the conserved tyrosine side chain.

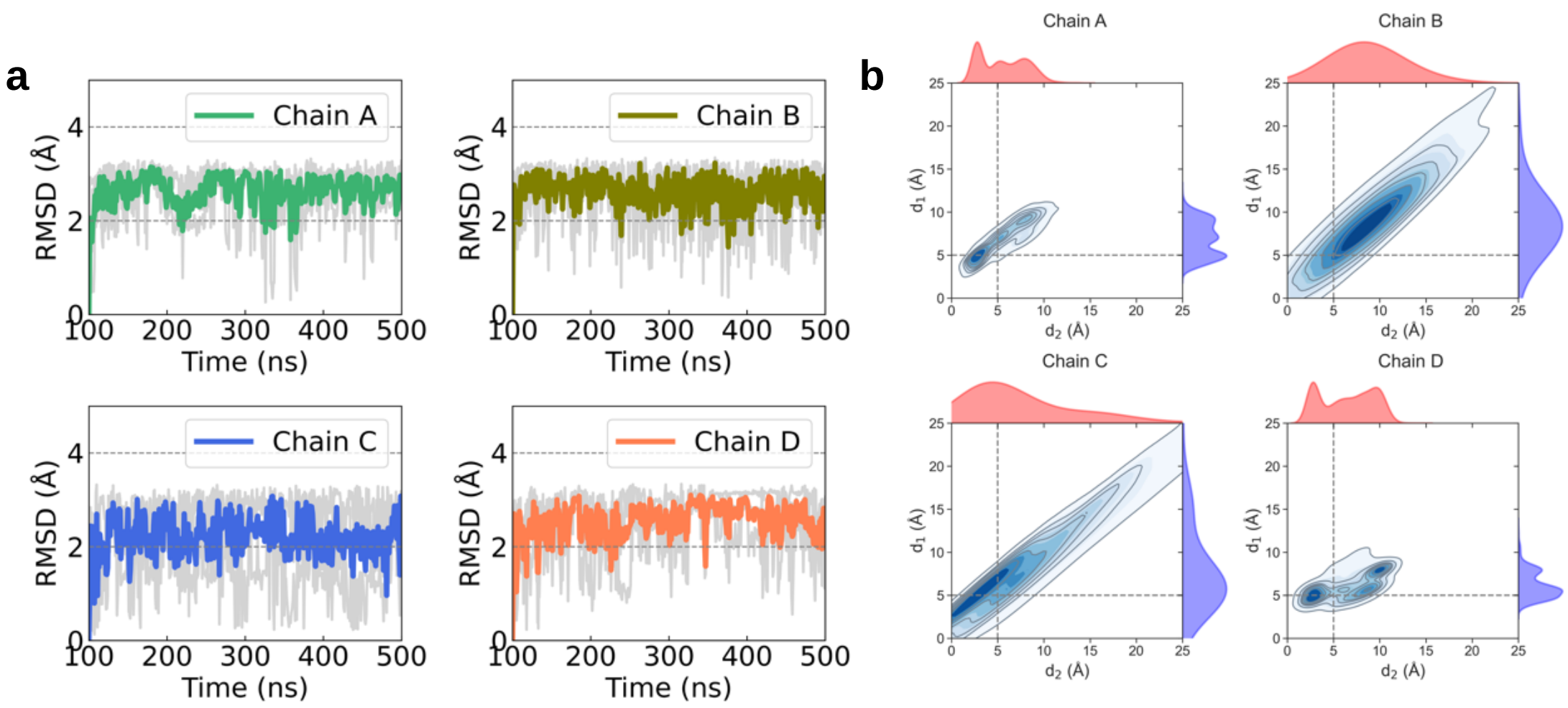

Supplementary Fig. 25. **a** RMSD values (Å) from 3x400 ns MD simulations of *Sr*BDH1 N32 ancestor complex with (+)-borneol. Individual replica trajectories are shown in gray, while the colored trace represents the mean RMSD (Å) averaged over the replicas. **b** Distributions of the two monitored catalytic distances (Å) across the simulation replicas; d_1 corresponds to the hydride transfer geometry between the C4 atom of the nicotinamide ring of NAD^+ and the oxidizable C5 carbon of borneol, and d_2 corresponds to the hydrogen-bond interaction between the borneol hydroxyl oxygen and the catalytic oxygen atom of the conserved tyrosine side chain.

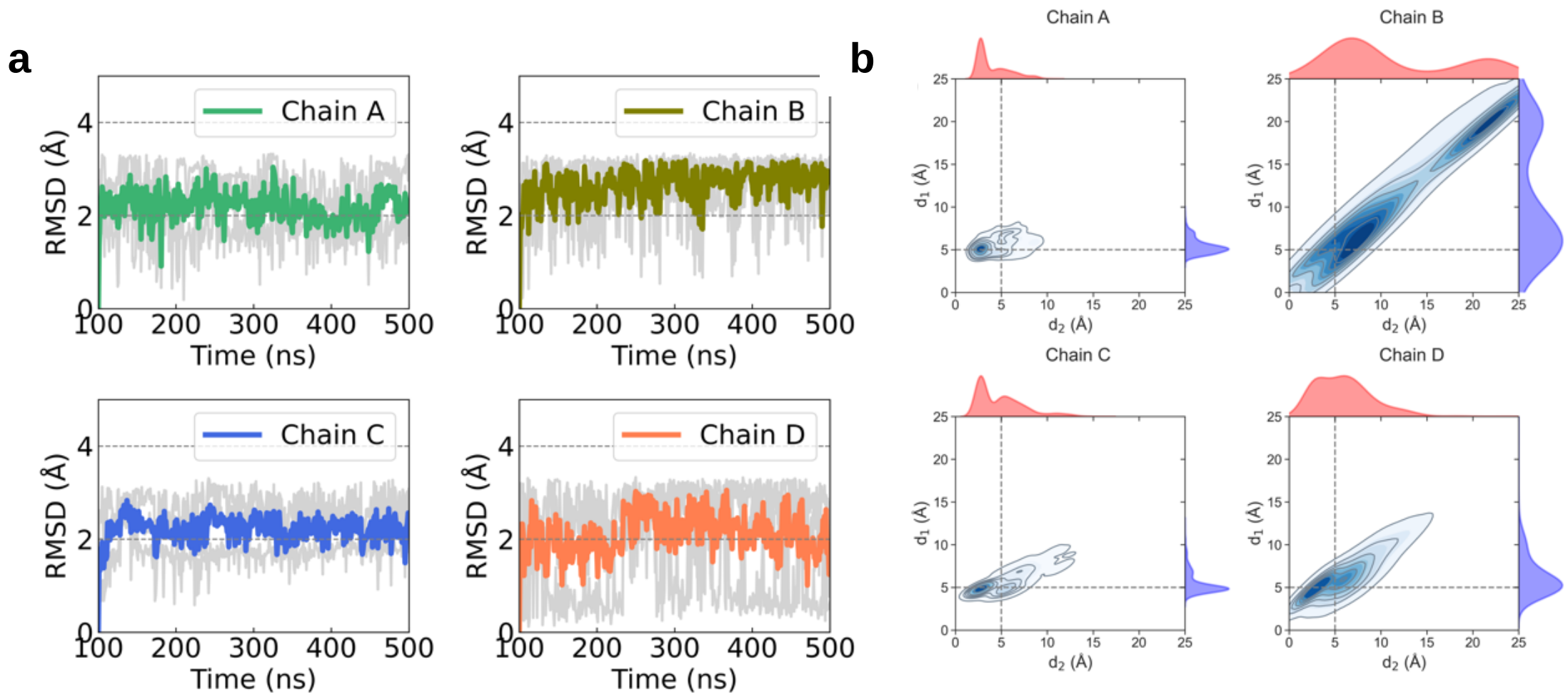

Supplementary Fig. 26. **a** RMSD values (Å) from 3x400 ns MD simulations of *Sr*BDH1 N32 ancestor complex with (−)-borneol. Individual replica trajectories are shown in gray, while the colored trace represents the mean RMSD (Å) averaged over the replicas. **b** Distributions of the two monitored catalytic distances (Å) across the simulation replicas; d_1 corresponds to the hydride transfer geometry between the C4 atom of the nicotinamide ring of NAD^+ and the oxidizable C5 carbon of borneol, and d_2 corresponds to the hydrogen-bond interaction between the borneol hydroxyl oxygen and the catalytic oxygen atom of the conserved tyrosine side chain.

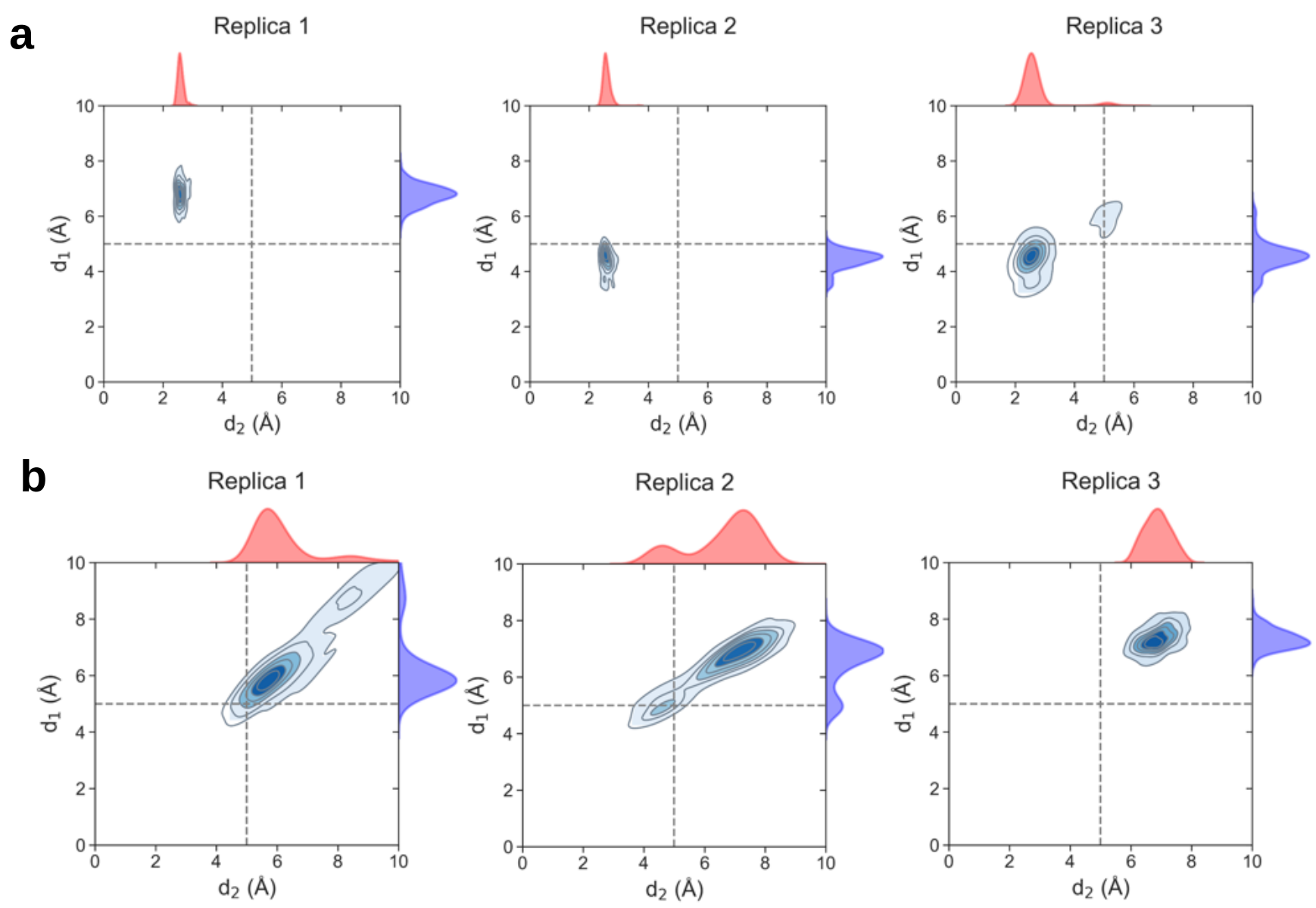

Supplementary Fig. 27. **Kernel density estimation (KDE) of the *Sr*BDH1 in complex with (+)- and (−)-borneol.** Kernel density estimation (KDE) of the catalytic distance distributions obtained from 10 ns ML/MM simulations of the *Sr*BDH1 in complex with **a** (+)- and **b** (−)-borneol. Distance d_1 (blue) corresponds to the hydride-transfer distance, while d_2 (red) represents the interaction between the catalytic tyrosine and the substrate. Three independent replicas were used for sampling.

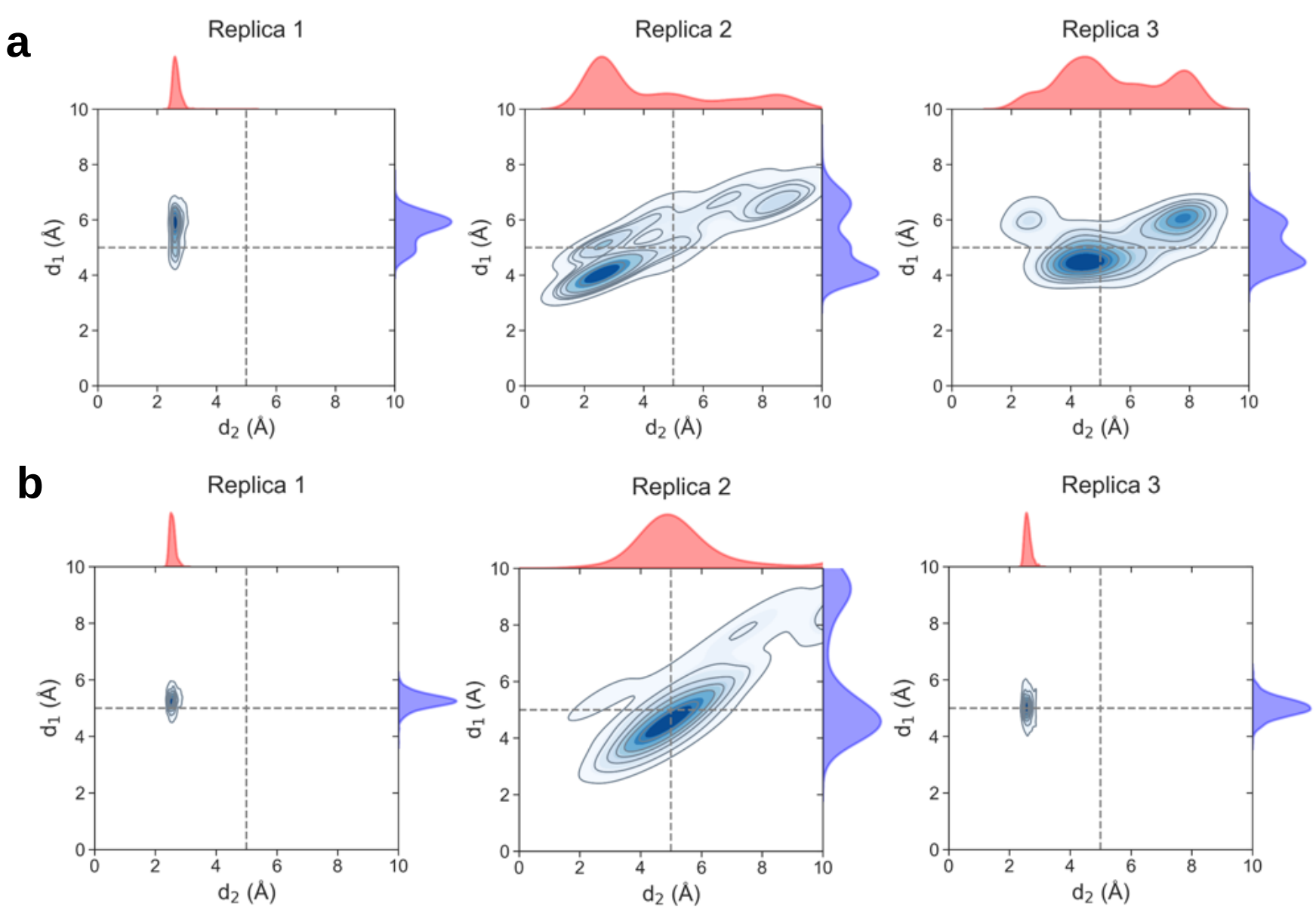

Supplementary Fig. 28. **Kernel density estimation (KDE) of the *Sr*BDH1 N32_I111L variant in complex with (+)- and (−)-borneol.** Kernel density estimation (KDE) of the catalytic distance distributions obtained from 10 ns ML/MM simulations of the *Sr*BDH1 N32_I111L variant in complex with **a** (+)- and **b** (−)-borneol. Distance d_1 (blue) corresponds to the hydride-transfer distance, while d_2 (red) represents the interaction between the catalytic tyrosine and the substrate. Three independent replicas were used for sampling.

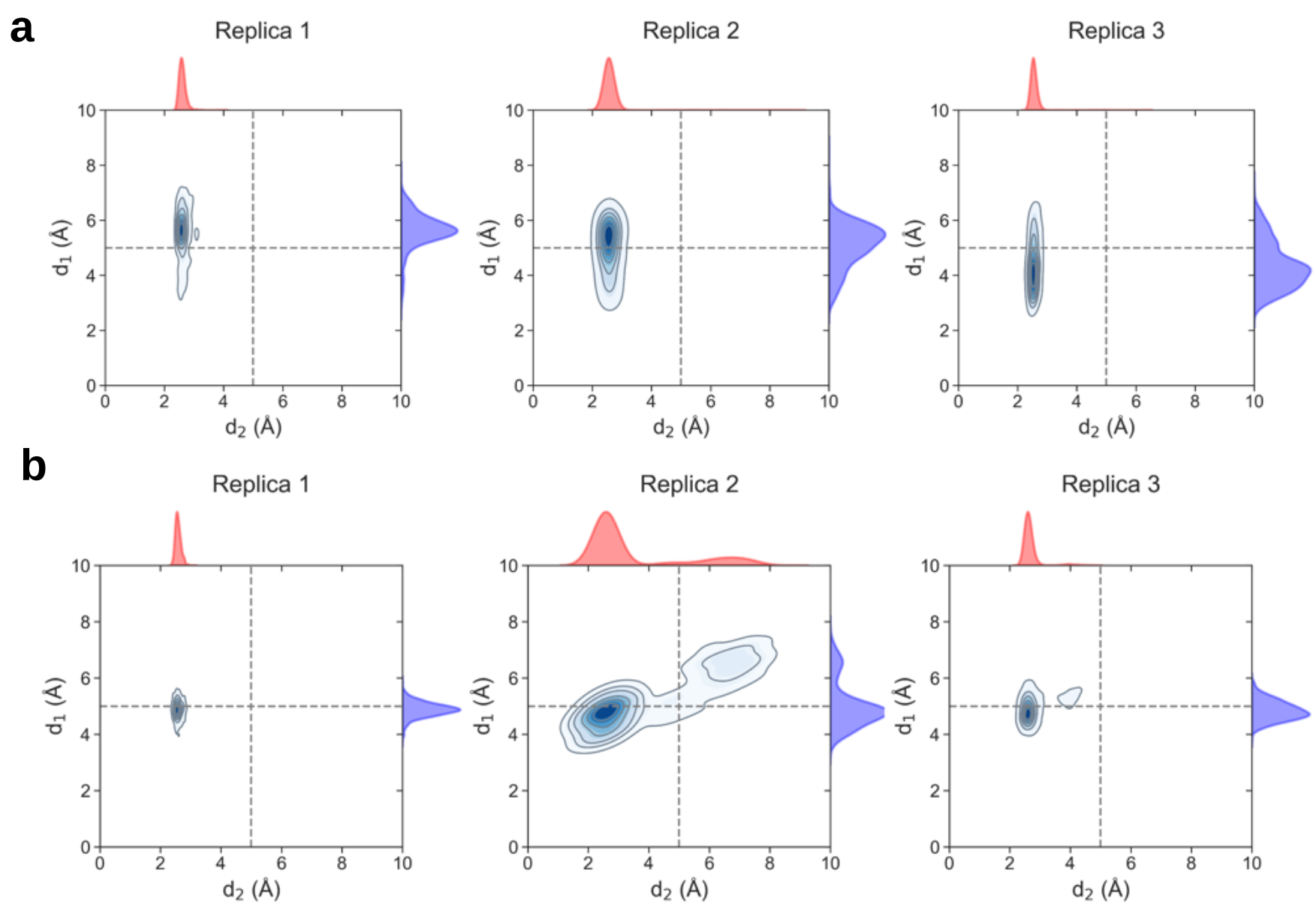

Supplementary Fig. 29. Kernel density estimation (KDE) of the catalytic distance distributions obtained from 10 ns ML/MM simulations of the *Sr*BDH1 N30_L111I variant in complex with **a** (+)- and **b** (−)-borneol. Distance d_1 (blue) corresponds to the hydride-transfer distance, while d_2 (red) represents the interaction between the catalytic tyrosine and the substrate. Three independent replicas were used for sampling.

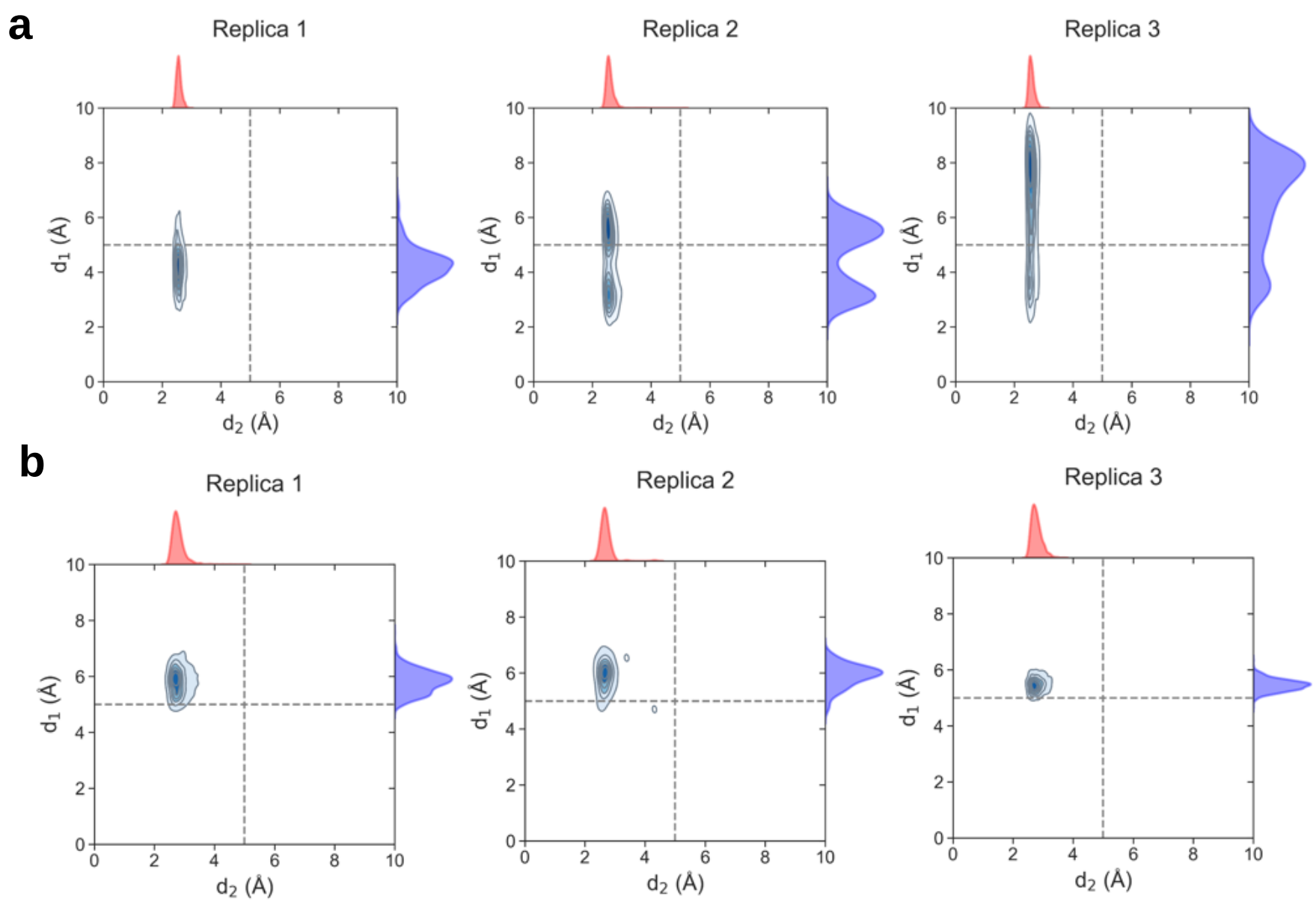

Supplementary Fig. 30. Kernel density estimation (KDE) of the catalytic distance distributions obtained from 10 ns ML/MM simulations of the *Sr*BDH1 N30_L111I/G169A/V136L/V183I variant in complex with **a** (+)- and **b** (−)-borneol. Distance d_1 (blue) corresponds to the hydride-transfer distance, while d_2 (red) represents the interaction between the catalytic tyrosine and the substrate. Three independent replicas were used for sampling.

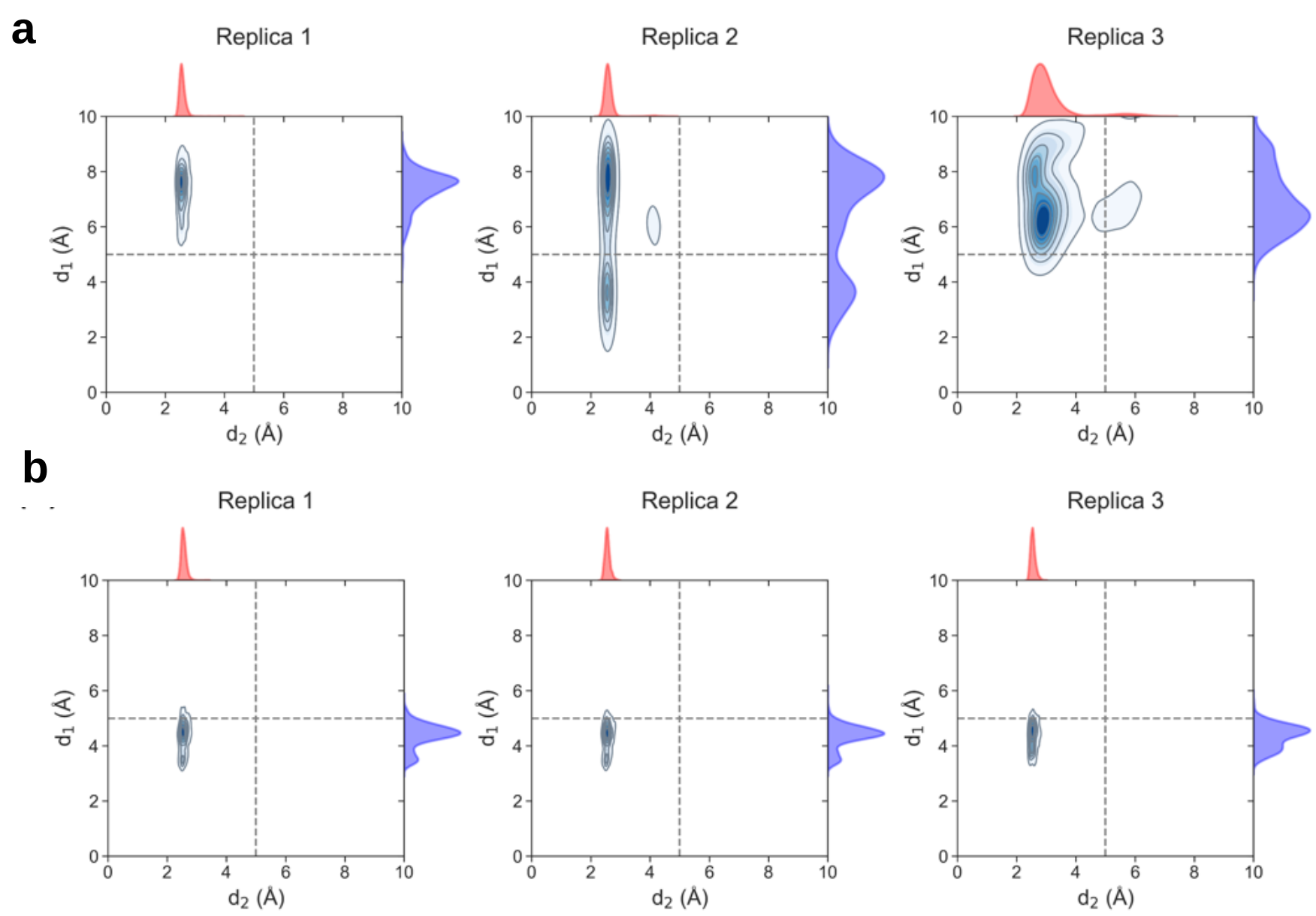

Supplementary Fig. 31. Kernel density estimation (KDE) of the catalytic distance distributions obtained from 10 ns ML/MM simulations of the *Sr*BDH1 N6 variant in complex with **a** (+)- and **b** (−)-borneol. Distance d_1 (blue) corresponds to the hydride-transfer distance, while d_2 (red) represents the interaction between the catalytic tyrosine and the substrate. Three independent replicas were used for sampling.

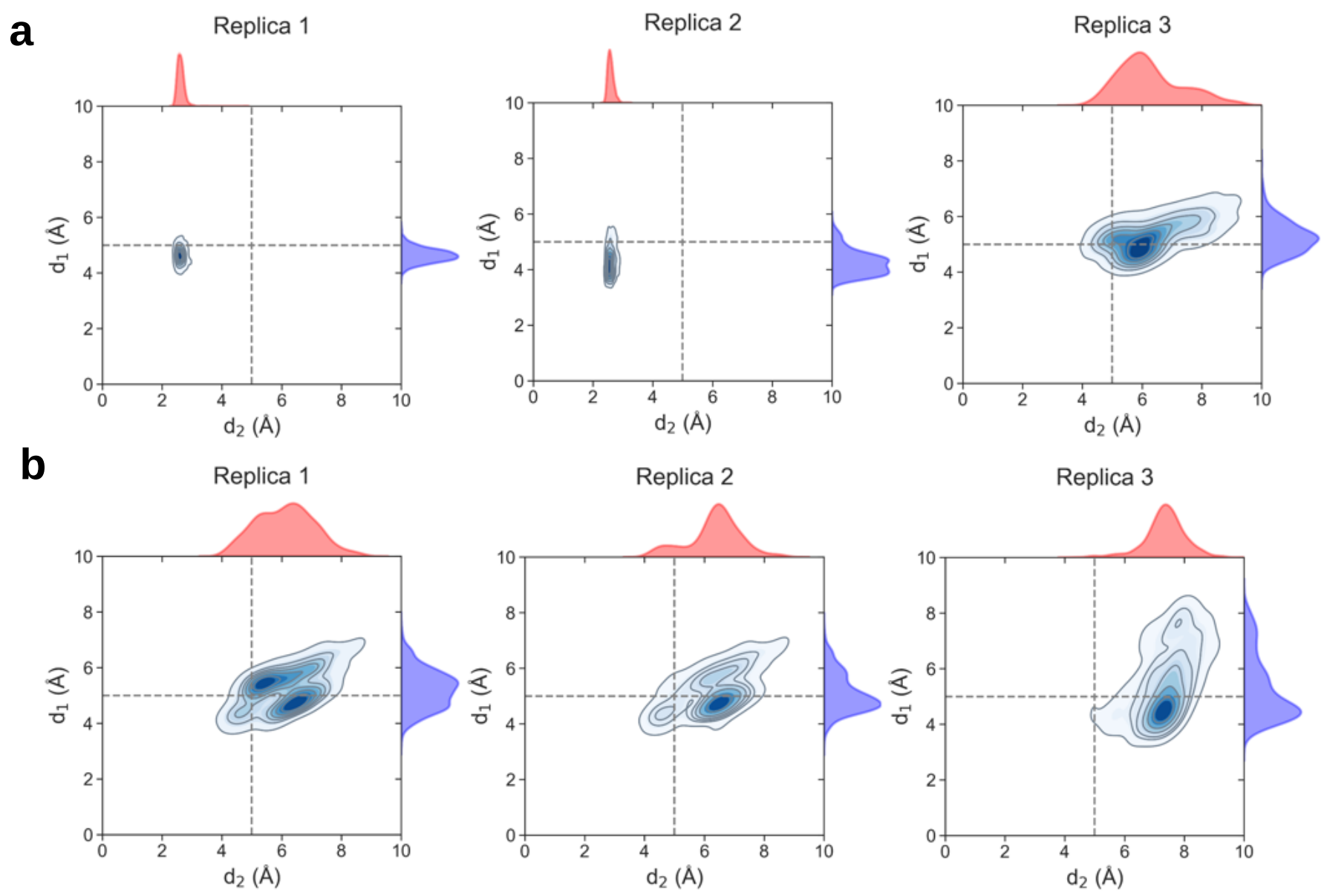

Supplementary Fig. 32. Kernel density estimation (KDE) of the catalytic distance distributions obtained from 10 ns ML/MM simulations of the *Sr*BDH1 N32 variant in complex with **a** (+)- and **b** (−)-borneol. Distance d_1 (blue) corresponds to the hydride-transfer distance, while d_2 (red) represents the interaction between the catalytic tyrosine and the substrate. Three independent replicas were used for sampling.

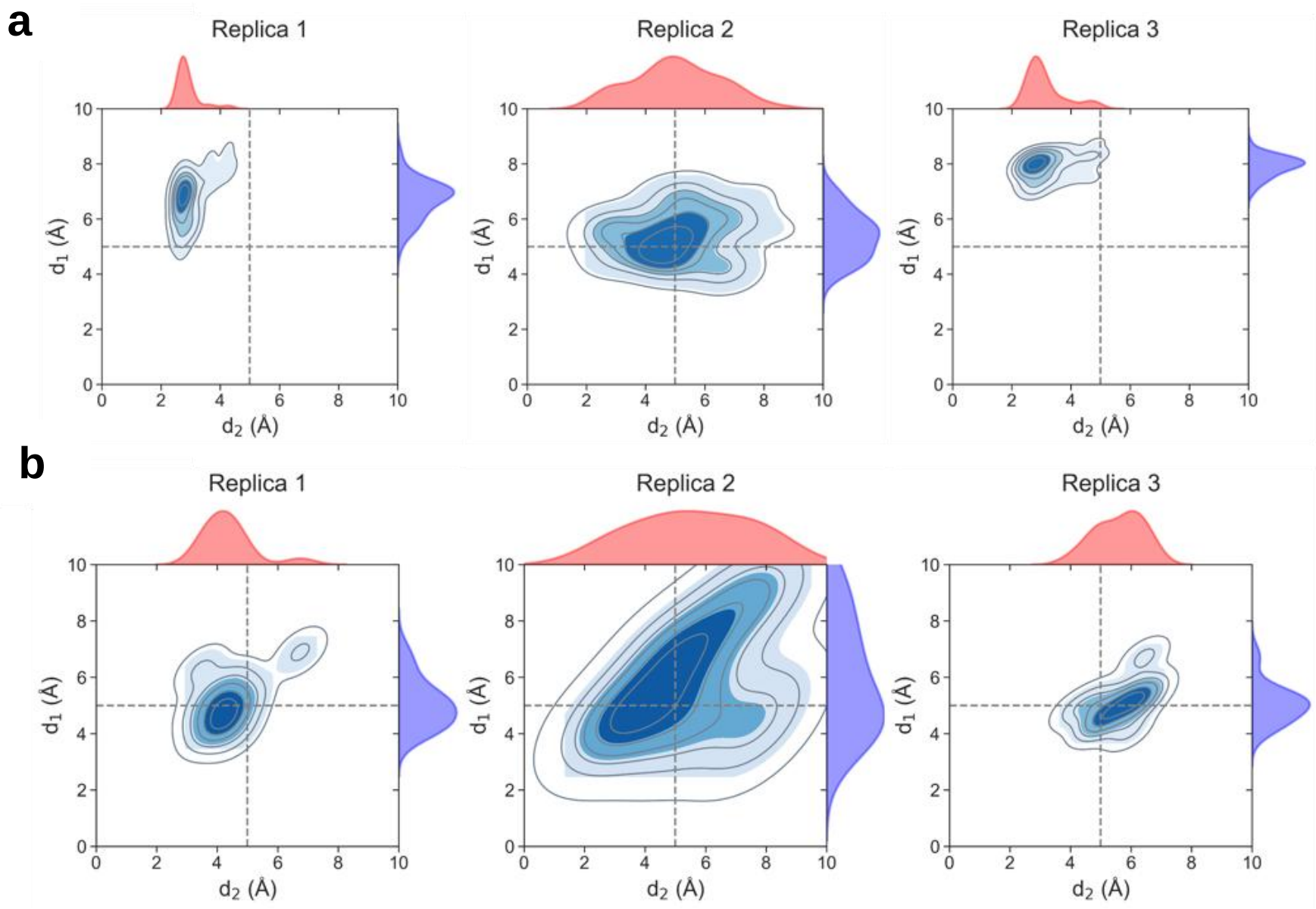

Supplementary Fig. 33. Kernel density estimation (KDE) of the catalytic distance distributions obtained from 10 ns conventional MD simulations of the *Sr*BDH1 wild-type in complex with **a** (+)- and **b** (−)-borneol. Distance d_1 (blue) corresponds to the hydride-transfer distance, while d_2 (red) represents the interaction between the catalytic tyrosine and the substrate. Three independent replicas were used for sampling.

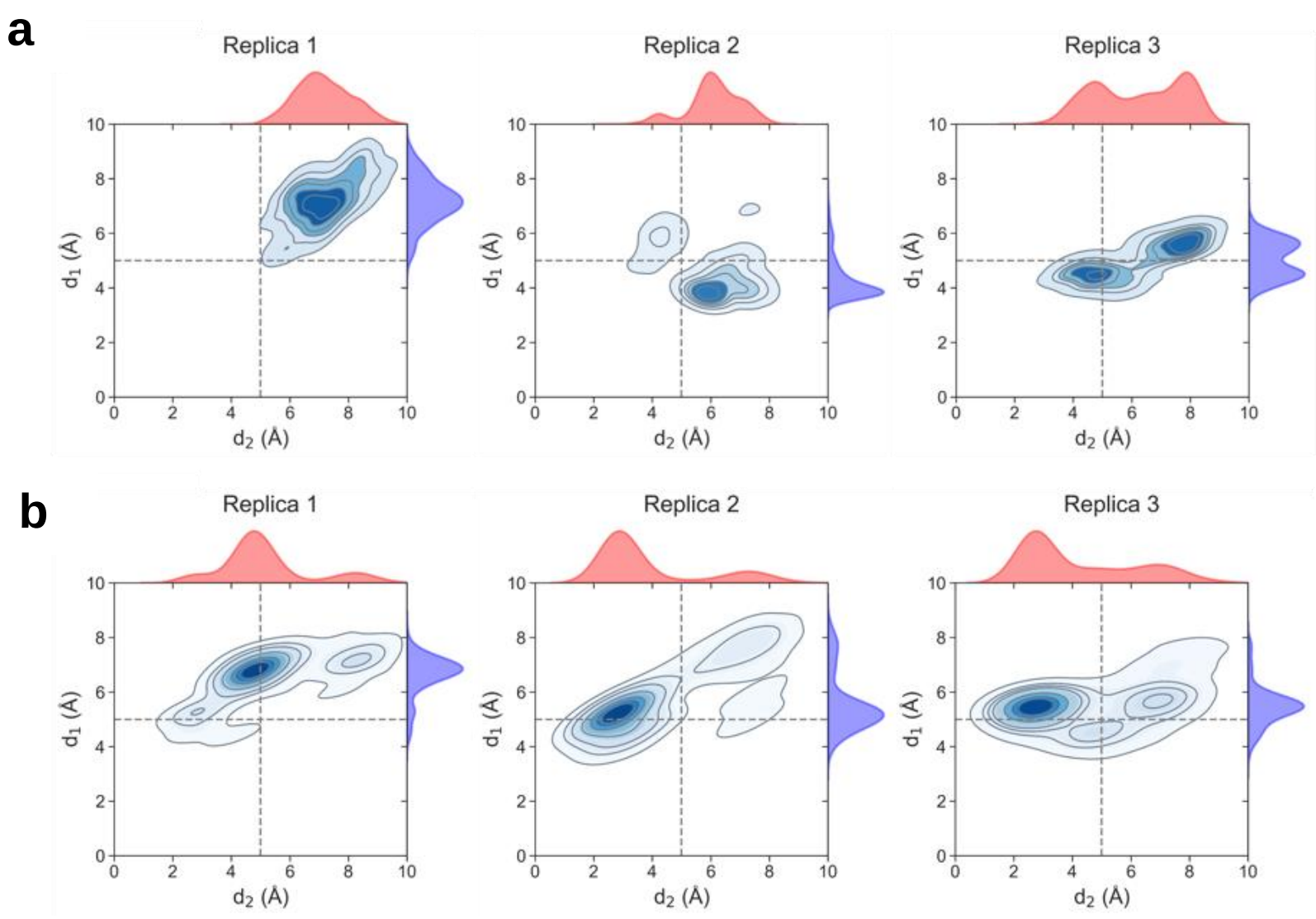

Supplementary Fig. 34. Kernel density estimation (KDE) of the catalytic distance distributions obtained from 10 ns conventional MD simulations of the *Sr*BDH1 N32_I111L variant in complex with **a** (+)- and **b** (−)-borneol. Distance d_1 (blue) corresponds to the hydride-transfer distance, while d_2 (red) represents the interaction between the catalytic tyrosine and the substrate. Three independent replicas were used for sampling.

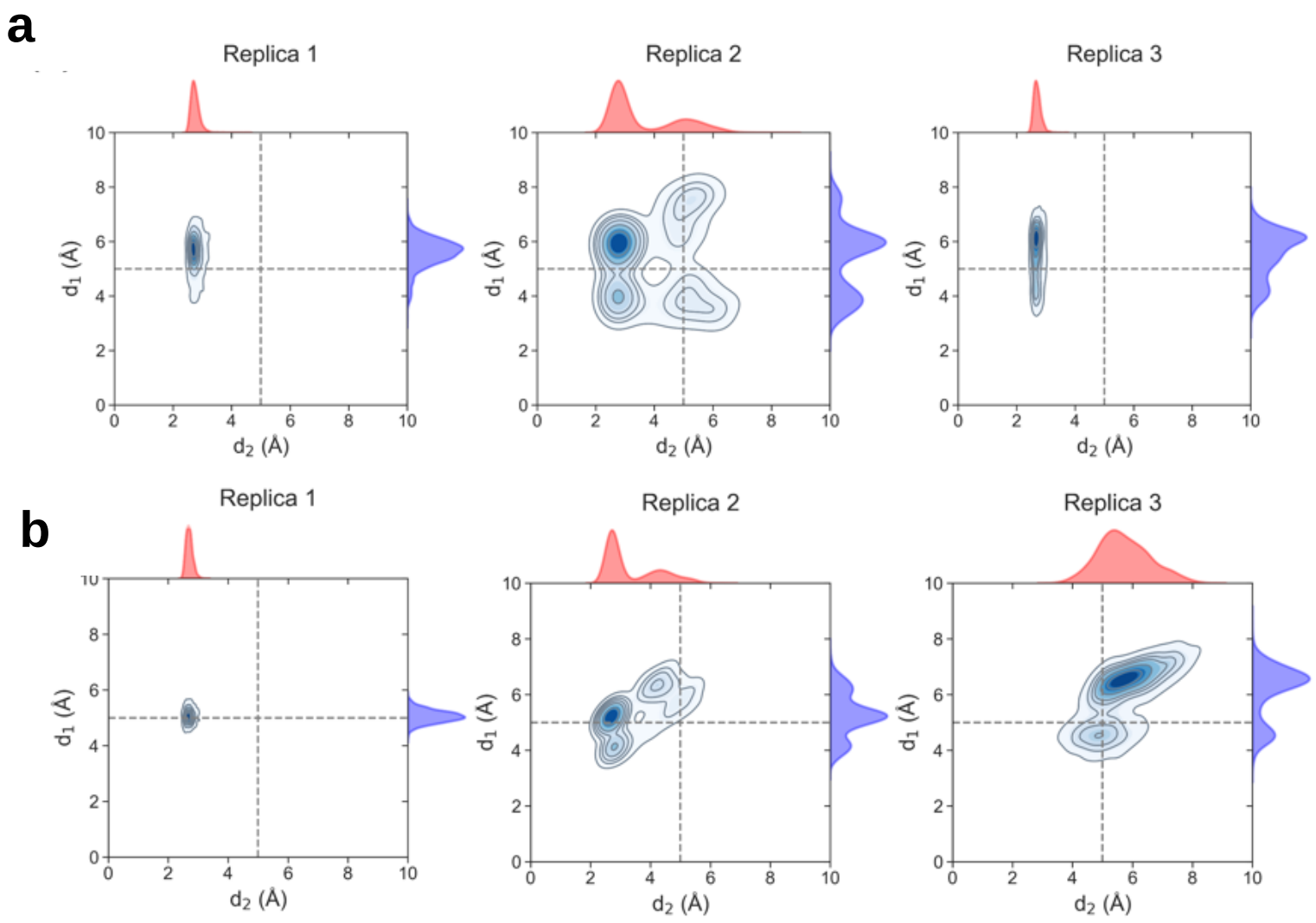

Supplementary Fig. 35. Kernel density estimation (KDE) of the catalytic distance distributions obtained from 10 ns conventional MD simulations of the *Sr*BDH1 N30_L111I variant in complex with **a** (+)- and **b** (−)-borneol. Distance d_1 (blue) corresponds to the hydride-transfer distance, while d_2 (red) represents the interaction between the catalytic tyrosine and the substrate. Three independent replicas were used for sampling.

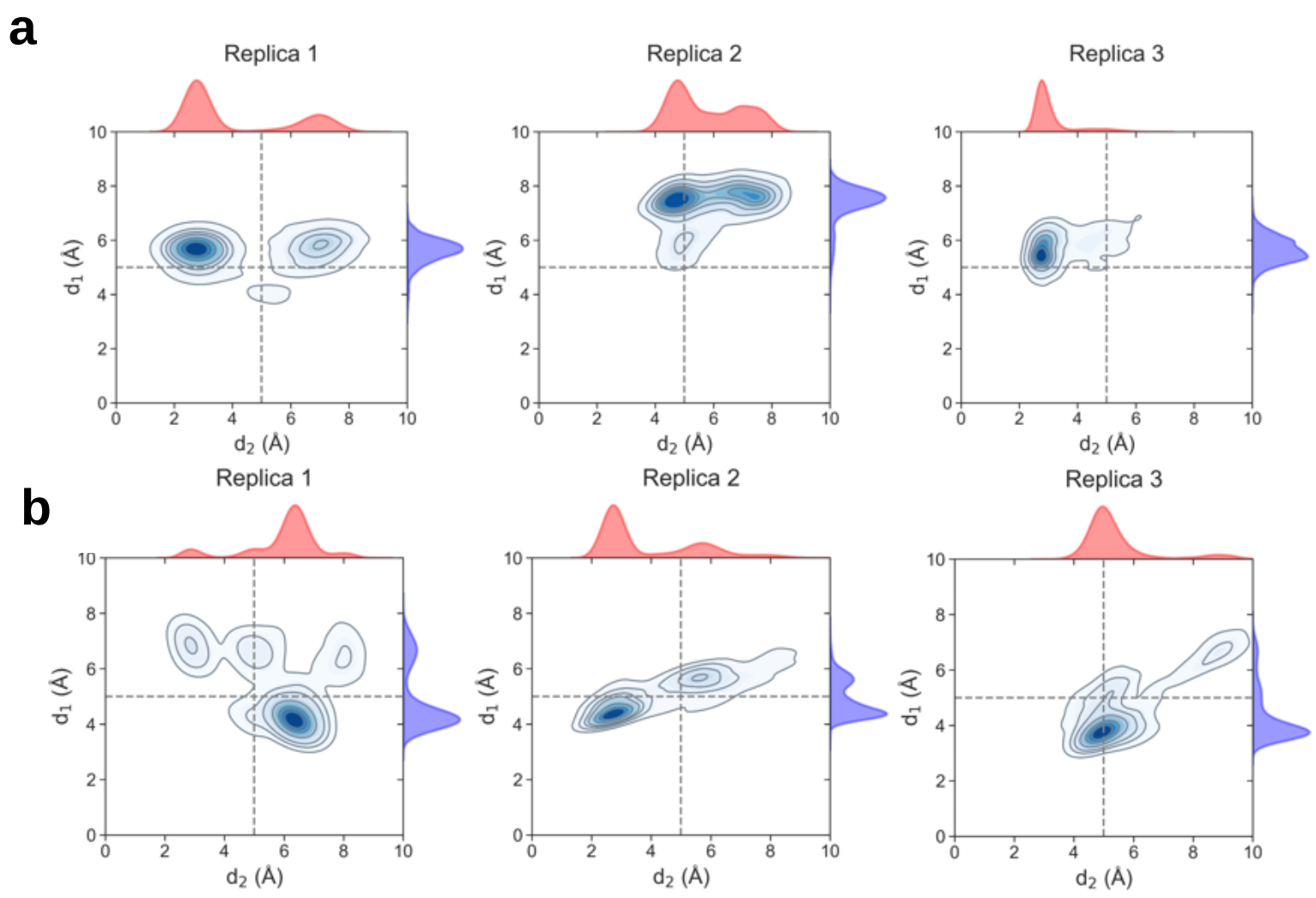

Supplementary Fig. 36. Kernel density estimation (KDE) of the catalytic distance distributions obtained from 10 ns conventional MD simulations of the *Sr*BDH1 N30_L111I/G169A/V136L/V183I variant in complex with **a** (+)- and **b** (−)-borneol. Distance d_1 (blue) corresponds to the hydride-transfer distance, while d_2 (red) represents the interaction between the catalytic tyrosine and the substrate. Three independent replicas were used for sampling.

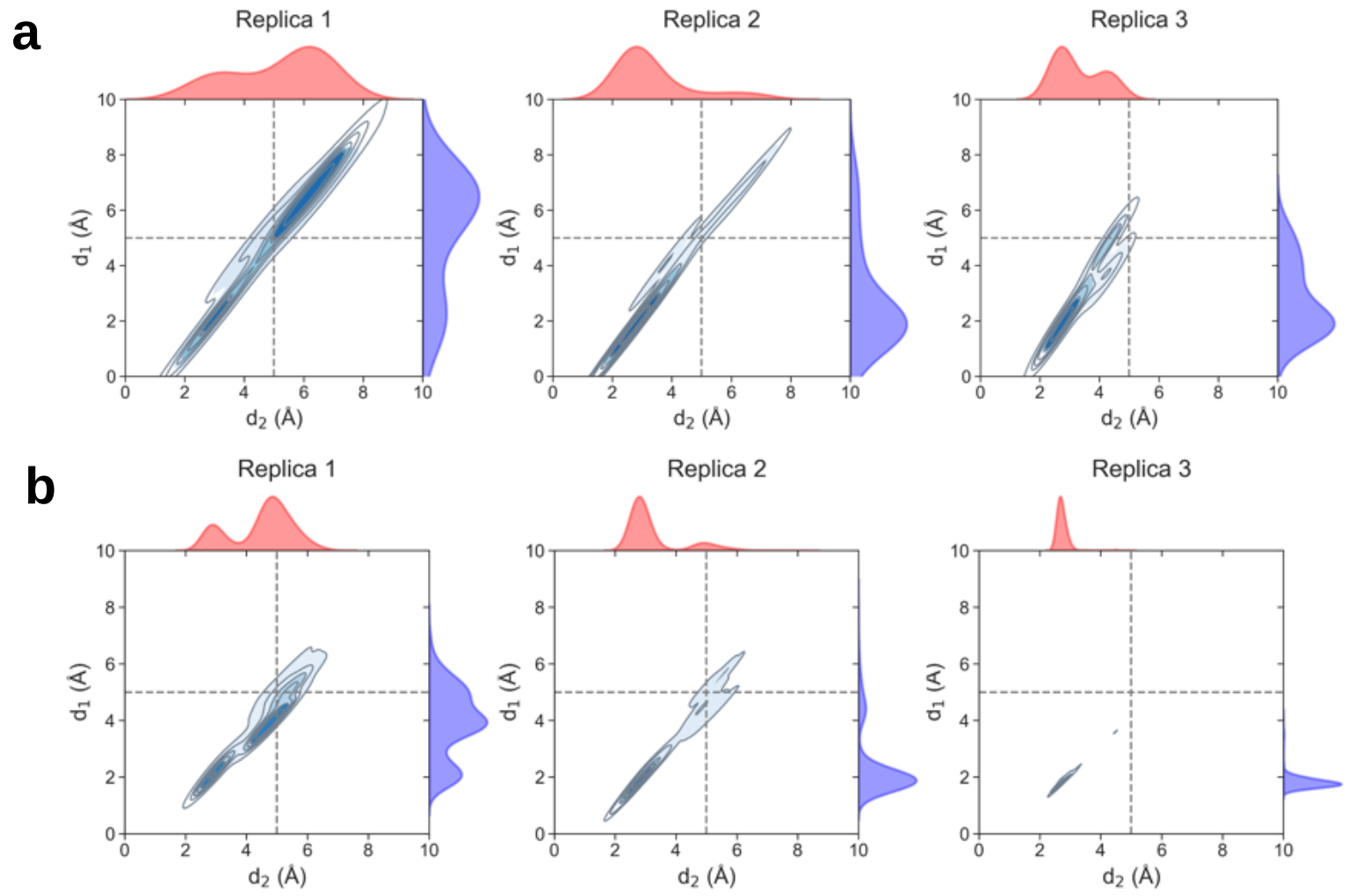

Supplementary Fig. 37. Kernel density estimation (KDE) of the catalytic distance distributions obtained from 10 ns conventional MD simulations of the *Sr*BDH1 N6 variant in complex with **a** (+)- and **b** (−)-borneol. Distance d_1 (blue) corresponds to the hydride-transfer distance, while d_2 (red) represents the interaction between the catalytic tyrosine and the substrate. Three independent replicas were used for sampling.

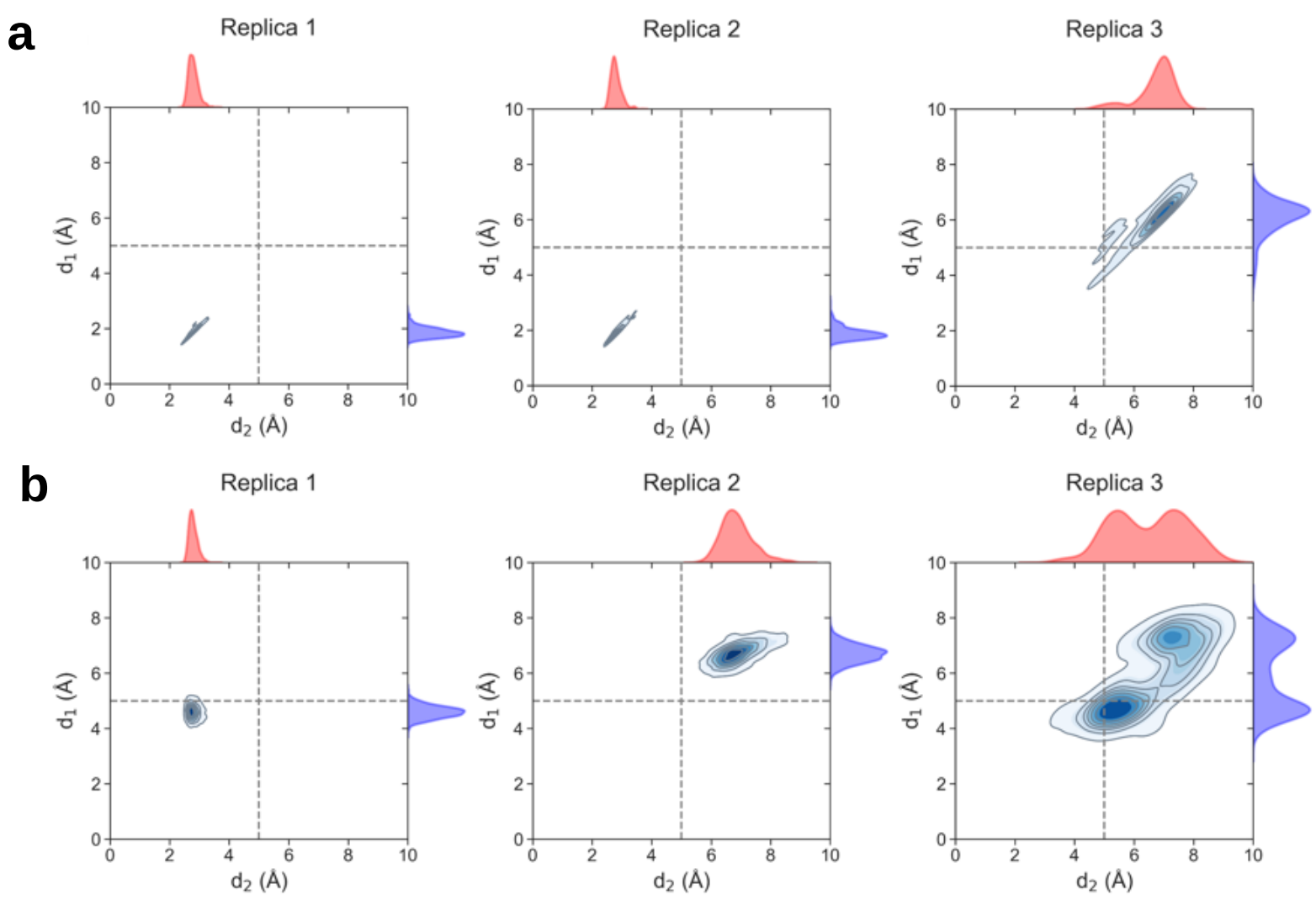

Supplementary Fig. 38. Kernel density estimation (KDE) of the catalytic distance distributions obtained from 10 ns conventional MD simulations of the *Sr*BDH1 N32 variant in complex with **a** (+)- and **b** (−)-borneol. Distance d_1 (blue) corresponds to the hydride-transfer distance, while d_2 (red) represents the interaction between the catalytic tyrosine and the substrate. Three independent replicas were used for sampling.

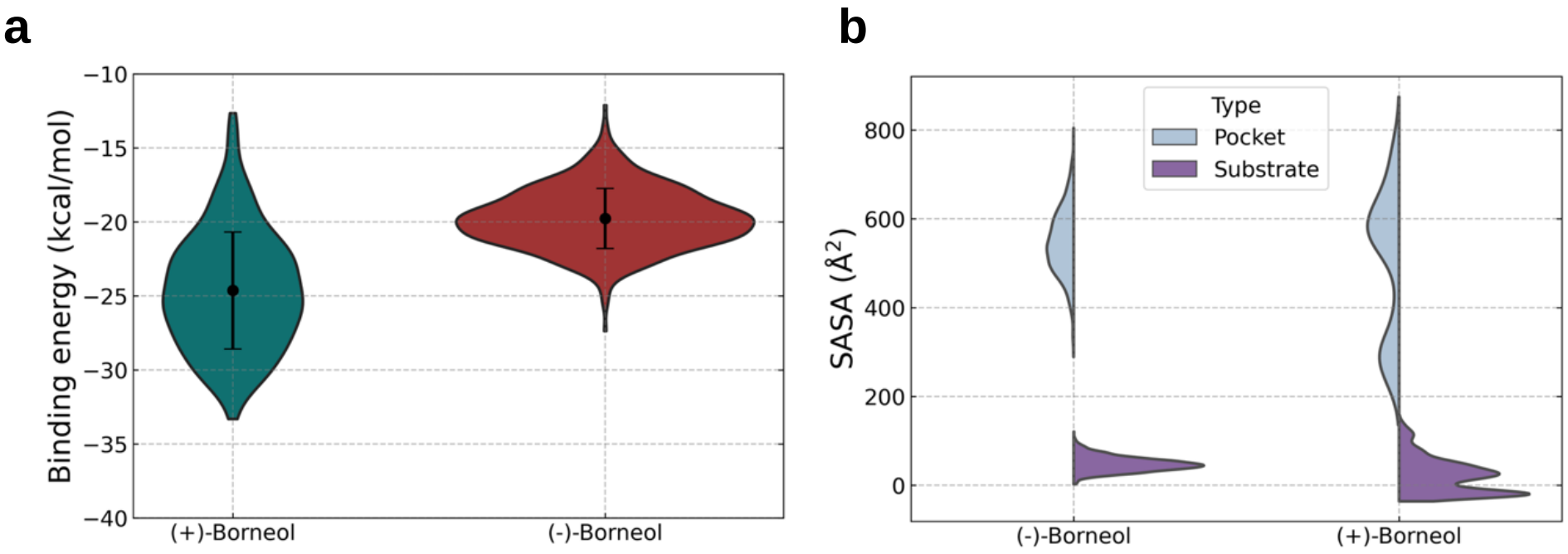

System	Mean ± SD
(+)-Borneol	-24.6 ± 4.0
(-)-Borneol	-19.8 ± 2.0

System	Pocket	Substrate
(-)-Borneol	546.5 ± 74.4	47.9 ± 16.6
(+)-Borneol	503.9 ± 166.0	21.0 ± 39.4

Supplementary Fig. 39. Violin plots of **a** total binding-energy from MM-ISMSA11 analysis for (+)-borneol as well as (−)-borneol and **b** of solvent-accessible surface area (SASA, Å²) for the binding pocket (grey blue) and the substrate (purple) obtained from 10 ns ML/MM 2–52–5 simulations within 3 replicas. Mean values and standard deviations are summarized in the accompanying tables.

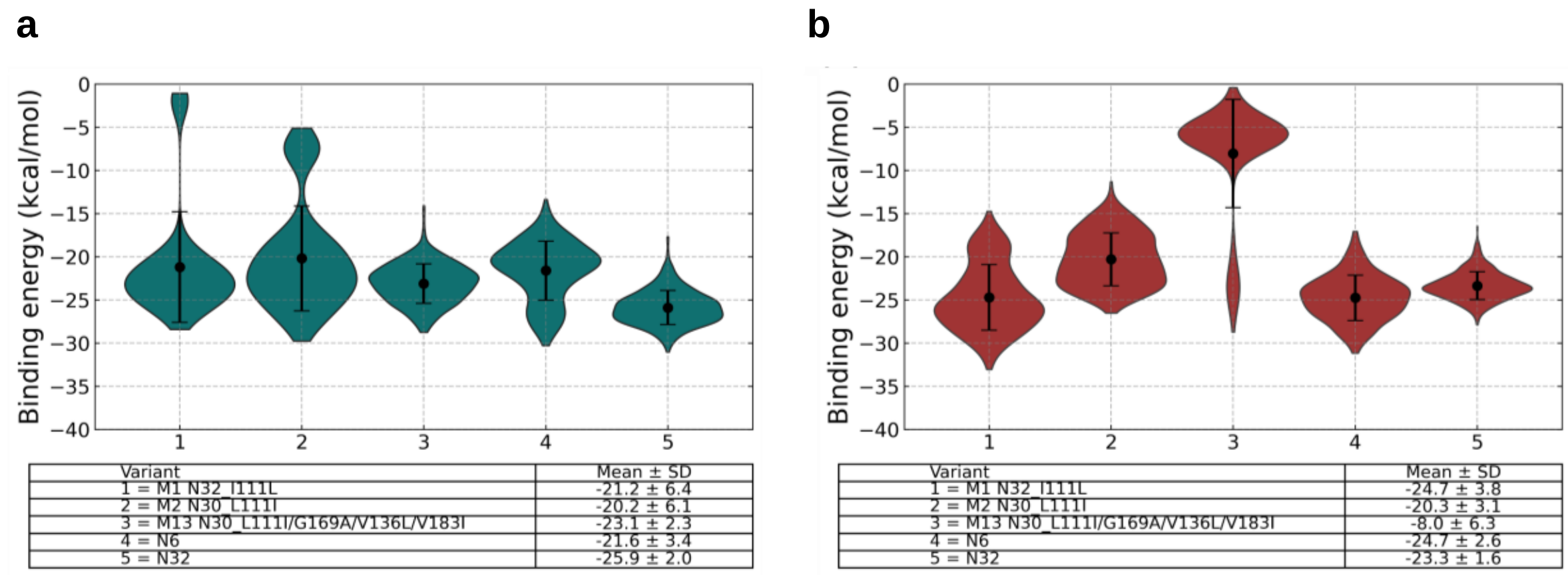

Supplementary Fig. 40. Mean MM-ISMSA11 binding energies in *Sr*BDH1 variants (N32_I111L, N30_L111I, N30_L111I/G169A/V136L/V183I, N6, N32) in complex with **a** (+)- and **b** (−)-borneol from 3x10 ns Torch-ANI AMBER ML/MM simulations2–52–5.

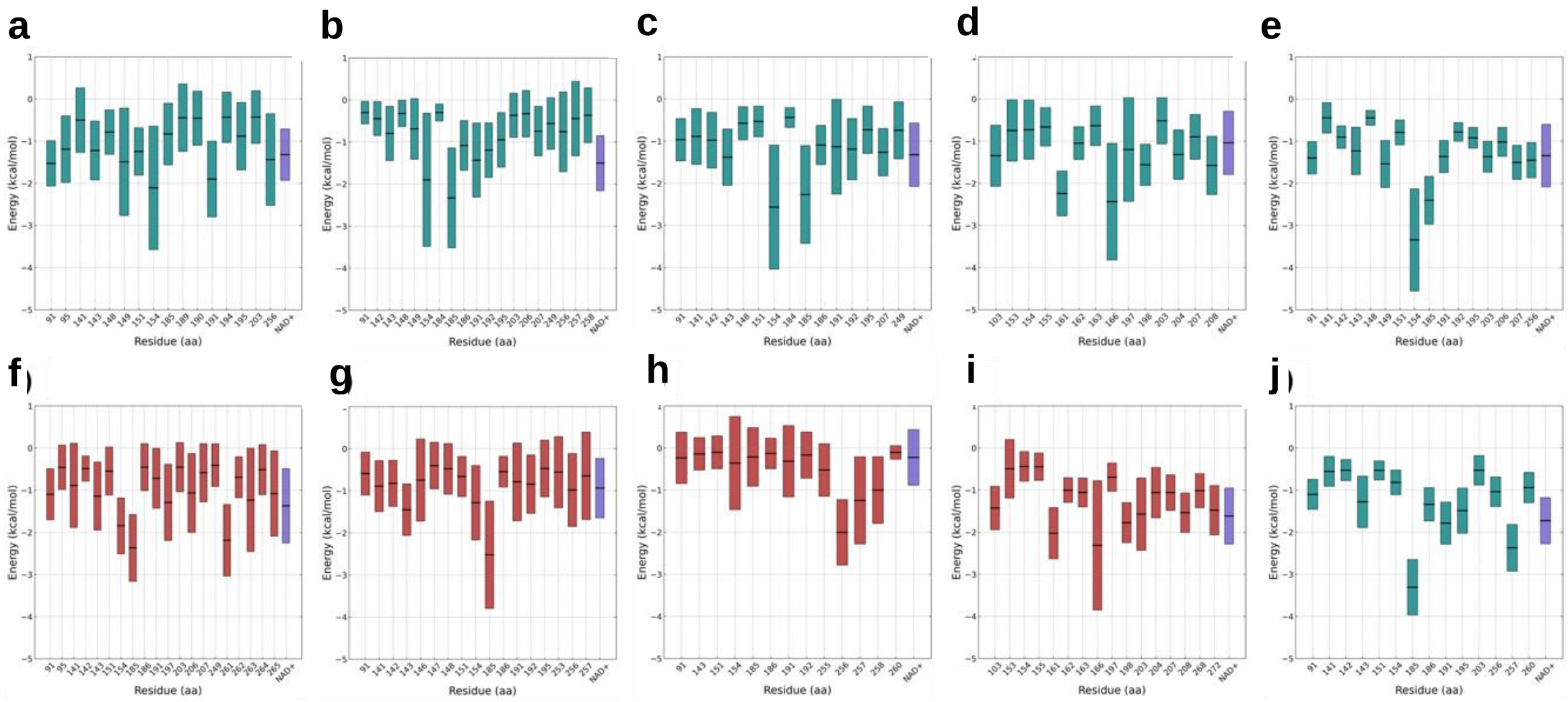

Supplementary Fig. 41. **Per-residue energy decomposition from MM-ISMSA analysis for (+)-borneol (a** to **e) (−)-borneol (f** to **j) from 3x10 ns Torch-ANI AMBER ML/MM simulations**2–52–5. Each plot displays the interaction energies for key residues within (**a**, **f**) N32_I111L, (**b**, **g**) N30_L111I, (**c**, **h**) N30_L111I/G169A/V136L/V183I, (**d**, **i**) N6, (e, j) N32 BDH. Shown here are energies for (+)-borneol (teal color) and (−)-borneol (firebrick) and NAD$^+$ (slate blue).

a

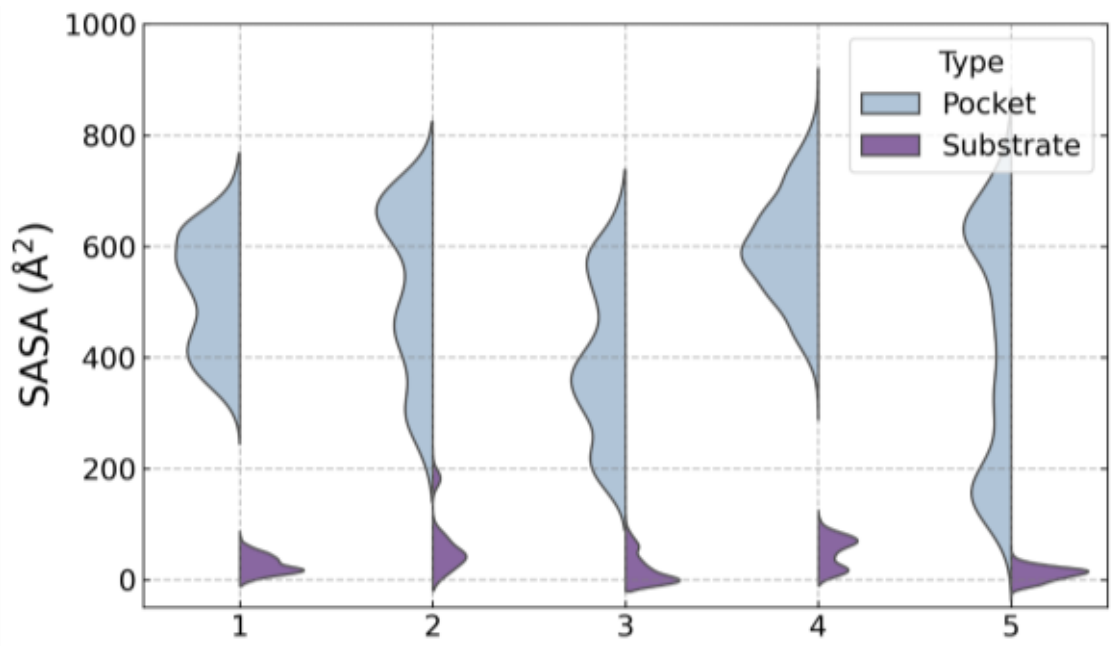

Node	Pocket	Substrate
1=M1 N32_I111L	517.6 ± 104.5	26.8 ± 16.6
2=M2 N30_L111I	520.7 ± 152.1	60.6 ± 50.7
3=M13 N30_L111I/G169A/V136L/V183I	397.1 ± 140.6	19.2 ± 27.2
4=N6	598.1 ± 98.2	50.2 ± 27.5
5=N32	424.8 ± 219.5	10.6 ± 12.8

b

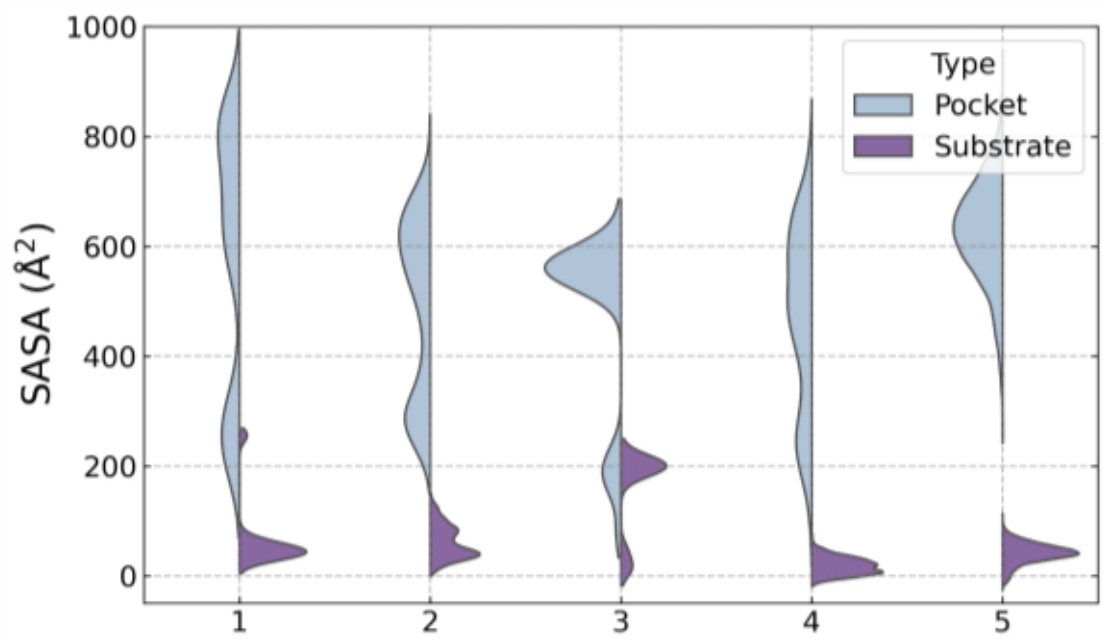

Node	Pocket	Substrate
1=M1 N32_I111L	572.6 ± 241.4	62.9 ± 57.9
2=M2 N30_L111I	489.8 ± 161.1	61.4 ± 30.1
3=M13 N30_L111I/G169A/V136L/V183I	472.2 ± 169.4	160.4 ± 75.5
4=N6	468.2 ± 166.7	18.3 ± 15.5
5=N32	624.9 ± 91.2	41.6 ± 18.6

Supplementary Fig. 42. Mean solvent accessible surface area (SASA, Å^2) distributions for pocket and substrate in *Sr*BDH1 variants (N32_I111L, N30_L111I, N30_L111I/G169A/V136L/V183I, N6, N32) in complex with **a** (+)- and **b** (−)-borneol from 3x10 ns Torch-ANI AMBER ML/MM simulations2–52–5.

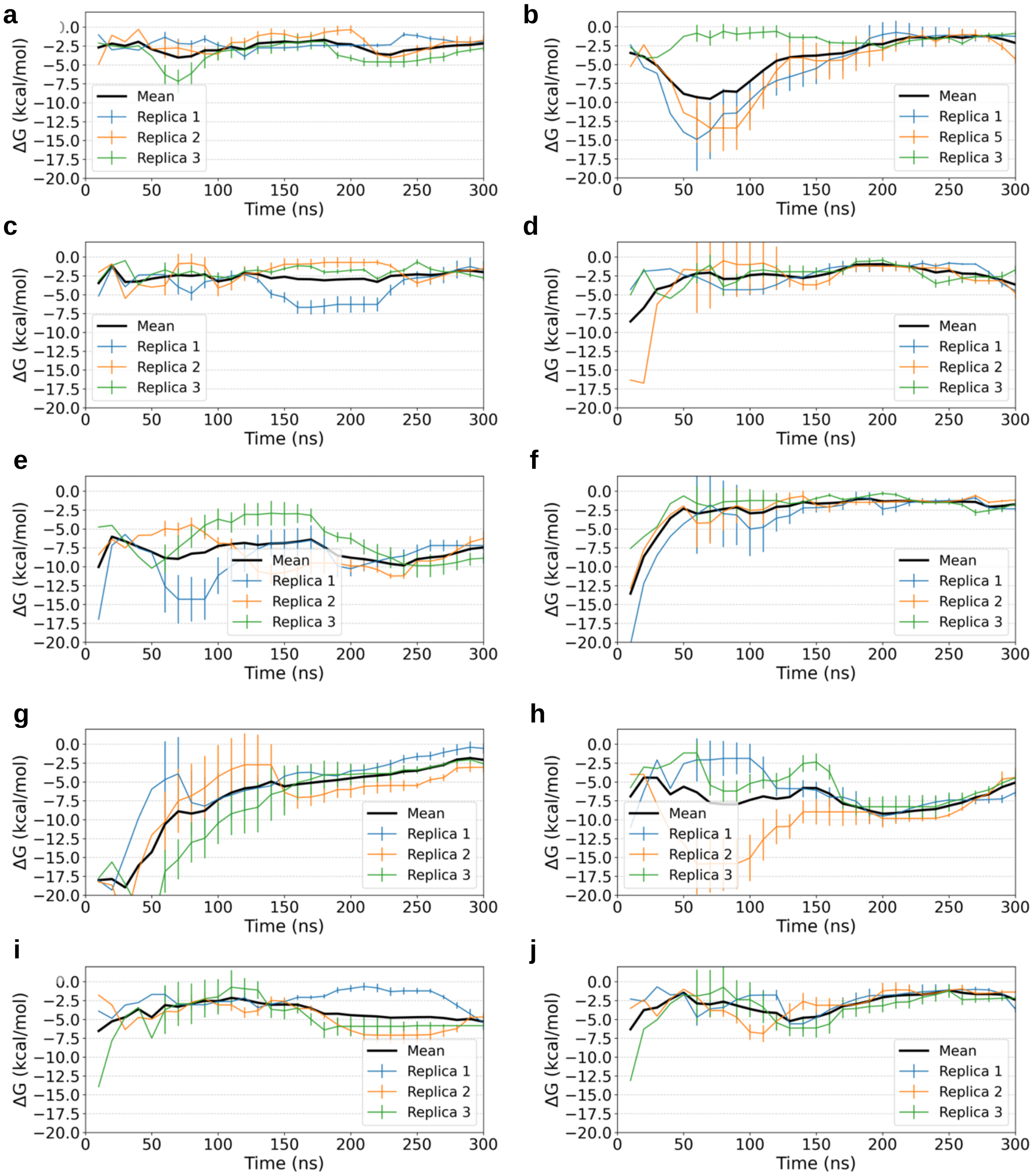

Supplementary Fig. 43. Binding free-energy profiles (ΔG) as a function of simulation time obtained from funnel metadynamics simulations6–106–10 of *Sr*BDH1 variants in complex with (+) and (−)-borneol. Panels show variants (**a**, **b**) N32_I111L, (**c**, **d**) N30_L111I, (**e**, **f**) N30_L111I/G169A/V136L/V183I, (**g**, **h**) N6, and (**i**, **j**) N32, with (+)-borneol on the left panels and (−)-borneol in the right panels. Three independent 300 ns replicas are shown as colored lines, while the corresponding mean ΔG profile is shown in black. Error bars indicate the standard deviation across replicas.

a

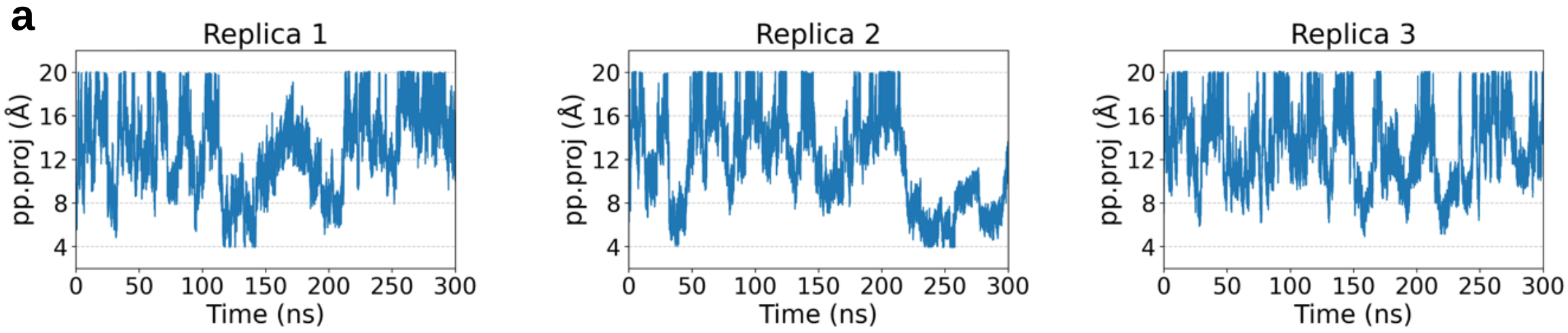

b

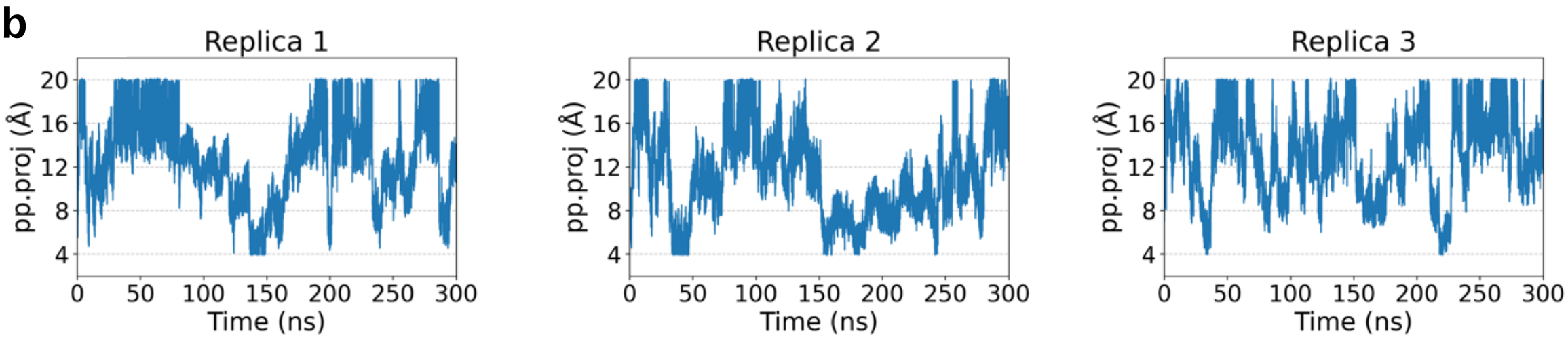

Supplementary Fig. 44. Time evolution of the funnel coordinate pp.proj along the funnel axis, during 300 ns funnel metadynamics simulations of wild-type *Sr*BDH1. Three independent replicas are shown for **a** (+)-borneol and **b** (−)-borneol, illustrating ligand movement along the binding pathway over time.

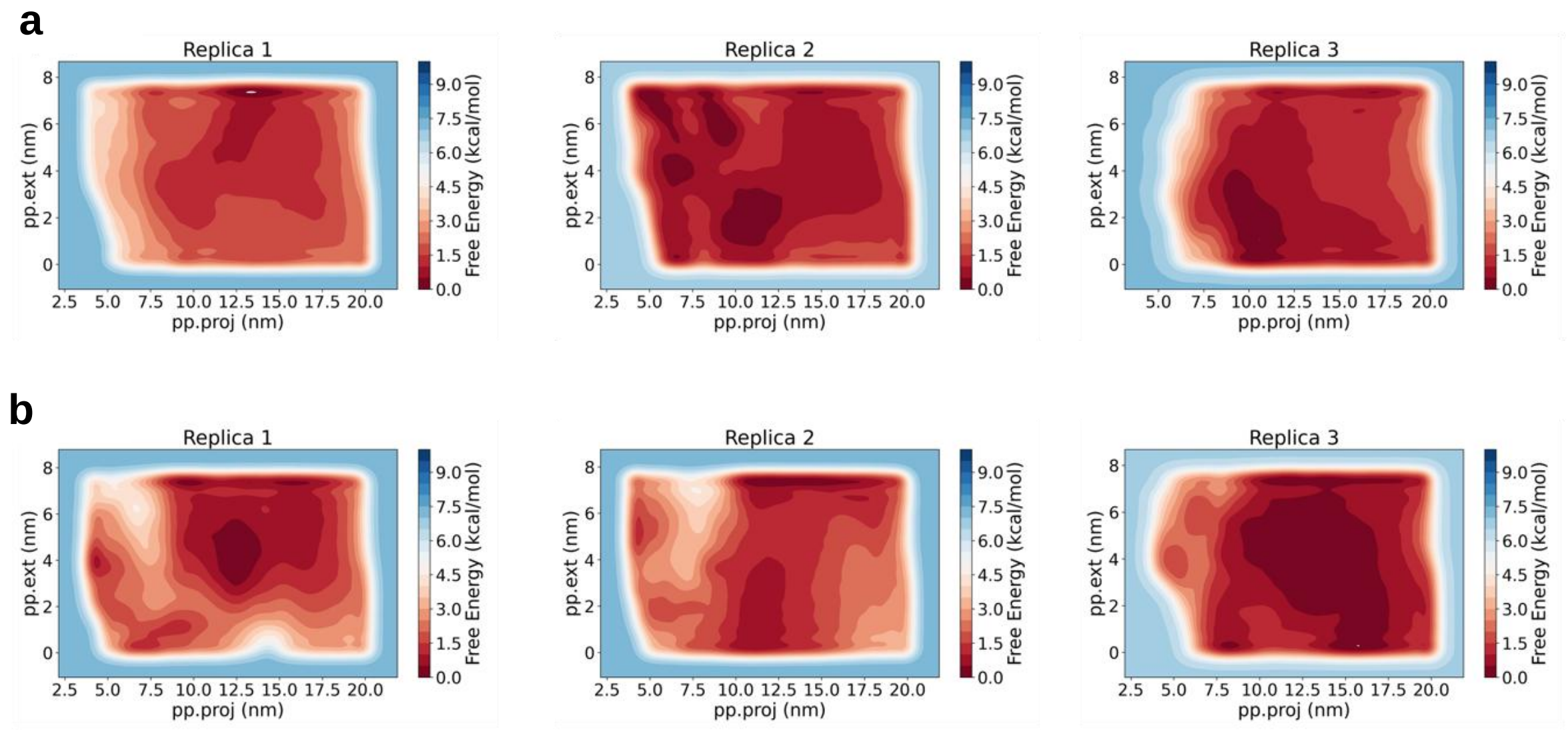

Supplementary Fig. 45. Two-dimensional free-energy surfaces projected onto the funnel collective variables pp.proj and pp.ext, obtained from funnel metadynamics simulations of wild-type *Sr*BDH1. Three independent replicas are shown for **a** (+)-borneol and **b** (−)-borneol.

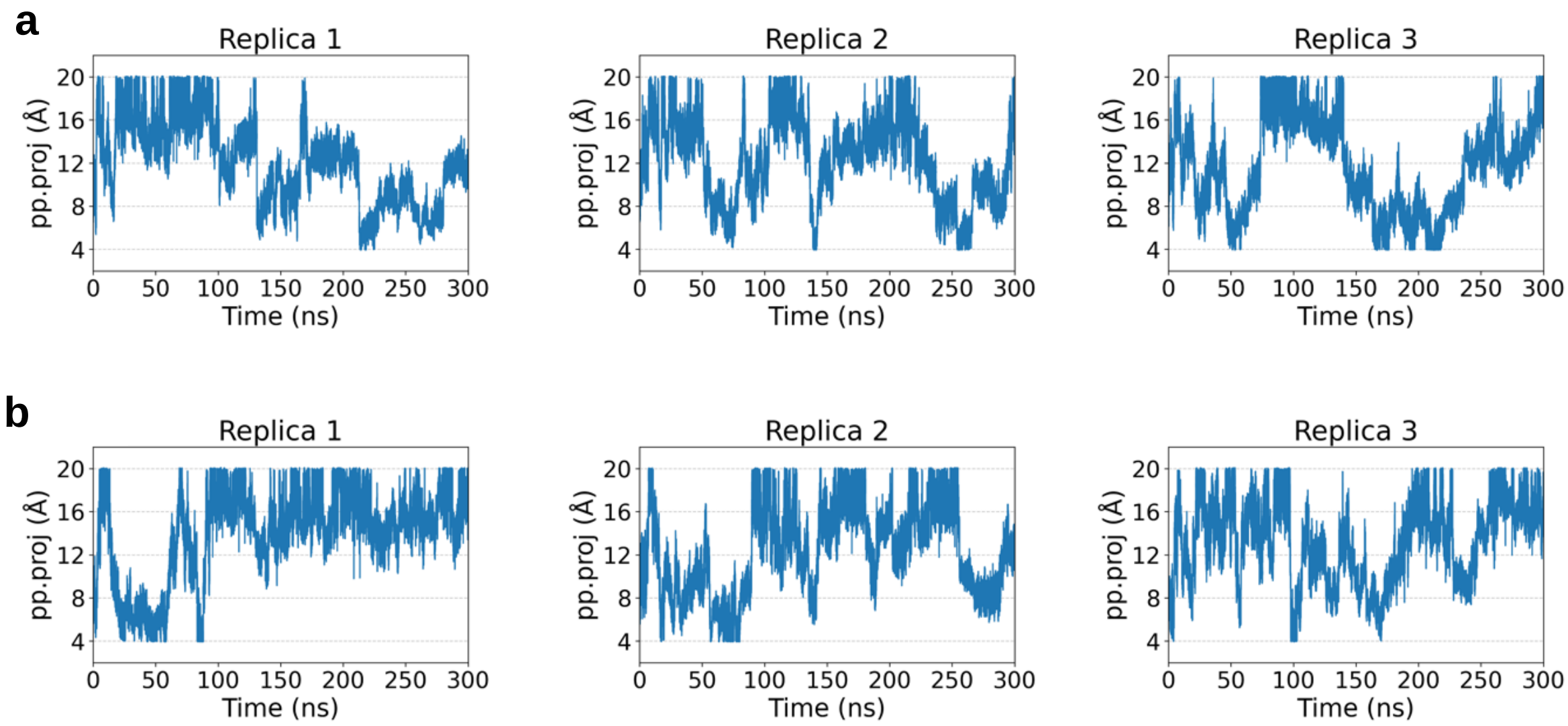

Supplementary Fig. 46. Time evolution of the funnel coordinate pp.proj along the funnel axis, during 300 ns funnel metadynamics simulations of the N32_I111L BDH variant. Three independent replicas are shown for **a** (+)-borneol and **b** (−)-borneol, illustrating ligand movement along the binding pathway over time.

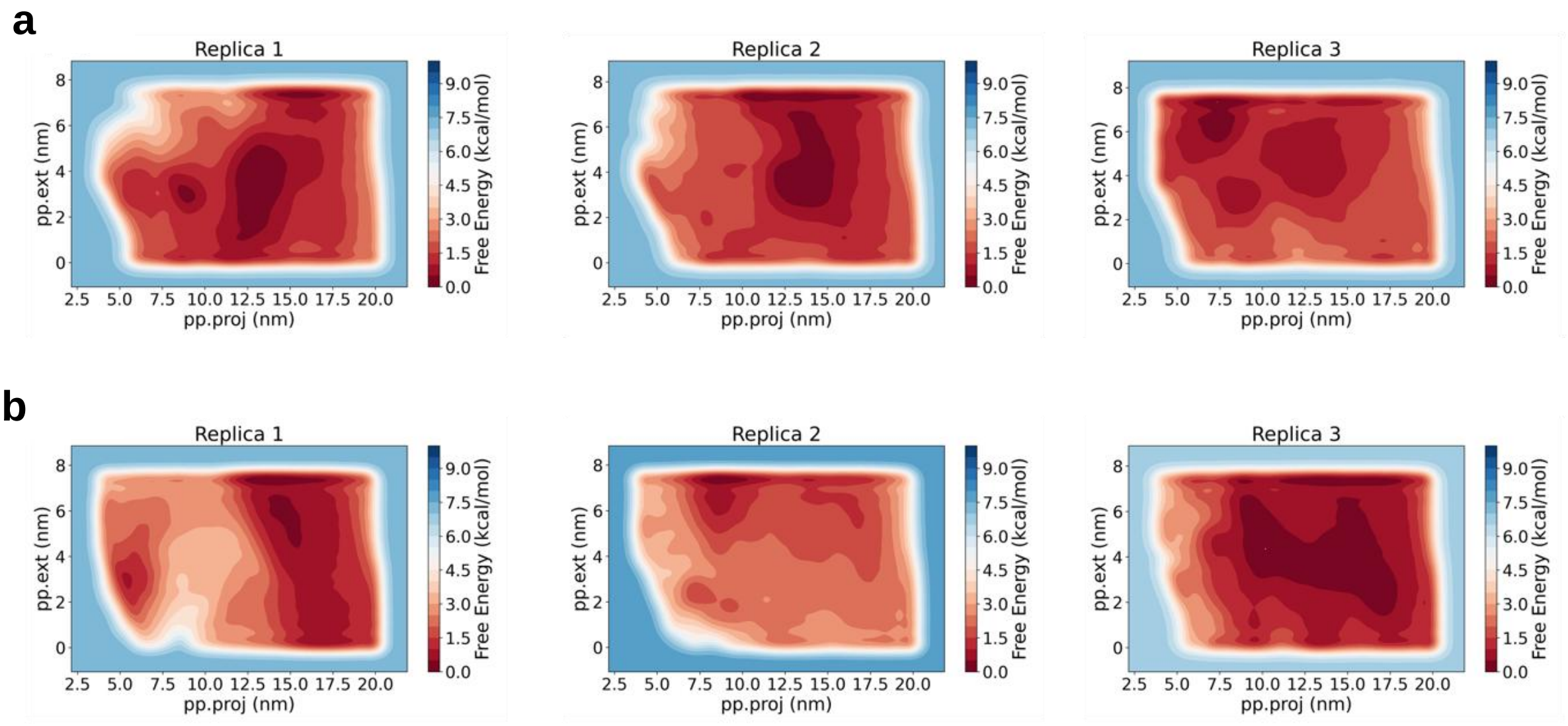

Supplementary Fig. 47. Two-dimensional free-energy surfaces projected onto the funnel collective variables pp.proj and pp.ext, obtained from funnel metadynamics simulations of the N32_I111L variant. Three independent replicas are shown for **a** (+)-borneol and **b** (−)-borneol.

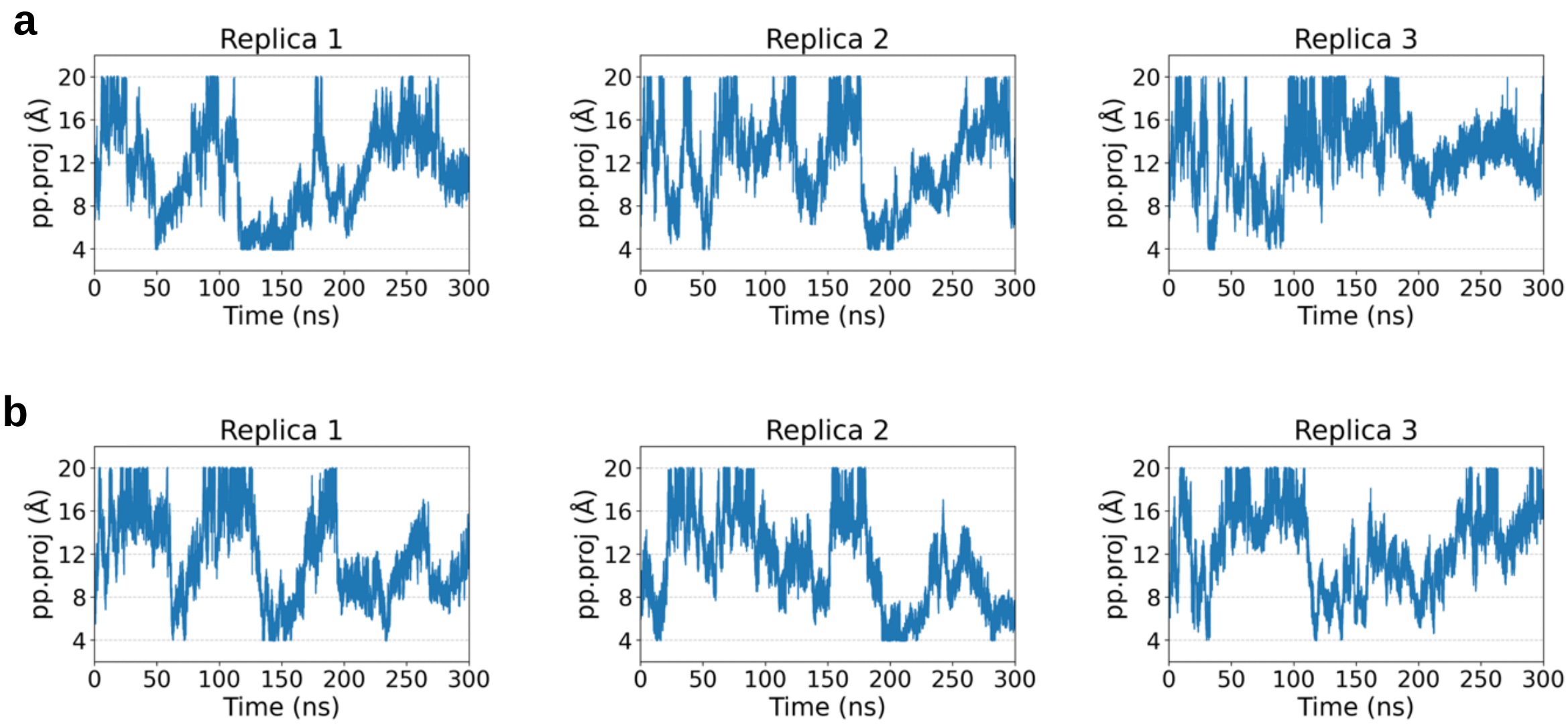

Supplementary Fig. 48. Time evolution of the funnel coordinate pp.proj along the funnel axis, during 300 ns funnel metadynamics simulations of the N30_L111I variant. Three independent replicas are shown for **a** (+)-borneol and **b** (−)-borneol, illustrating ligand movement along the binding pathway over time.

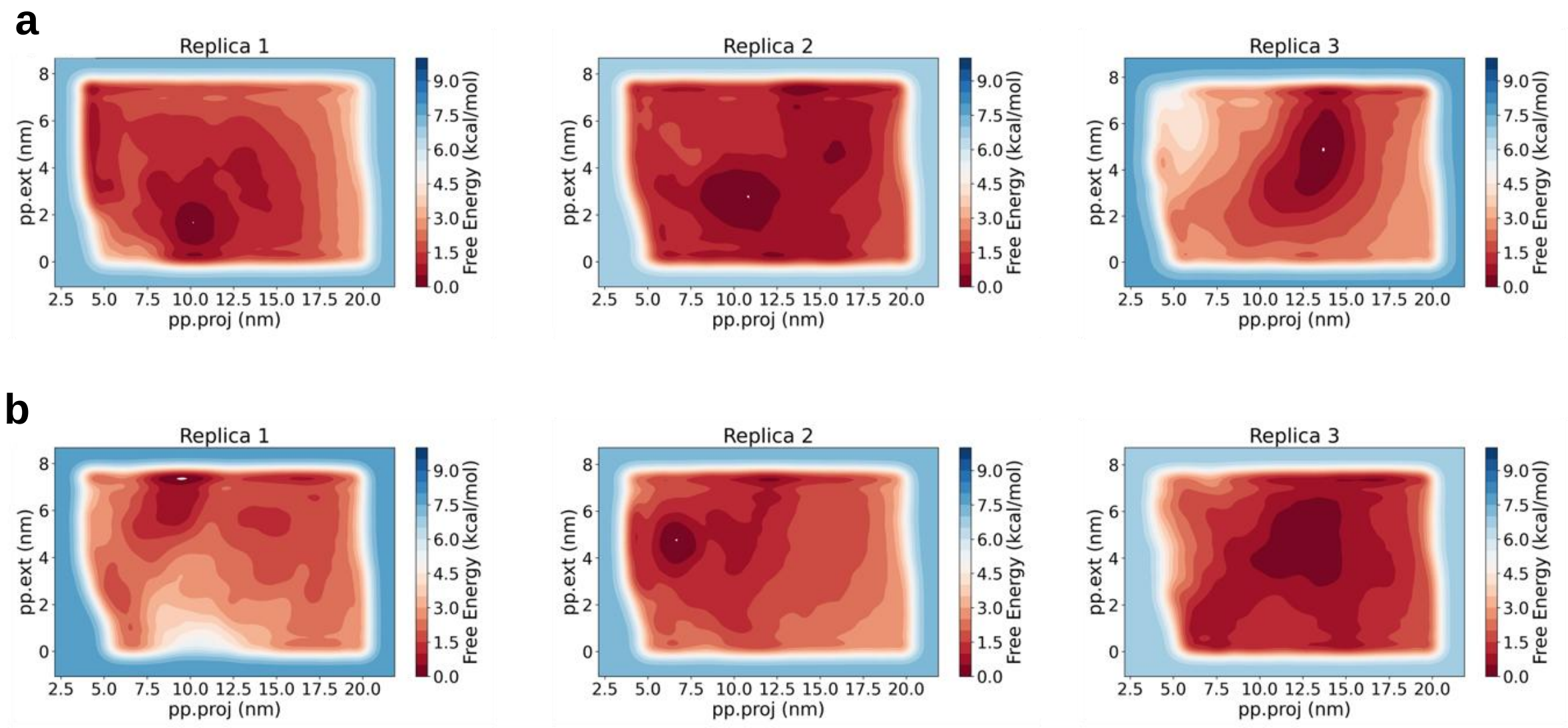

Supplementary Fig. 49. Two-dimensional free-energy surfaces projected onto the funnel collective variables pp.proj and pp.ext, obtained from funnel metadynamics simulations of the N30_L111I variant. Three independent replicas are shown for **a** (+)-borneol and **b** (−)-borneol.

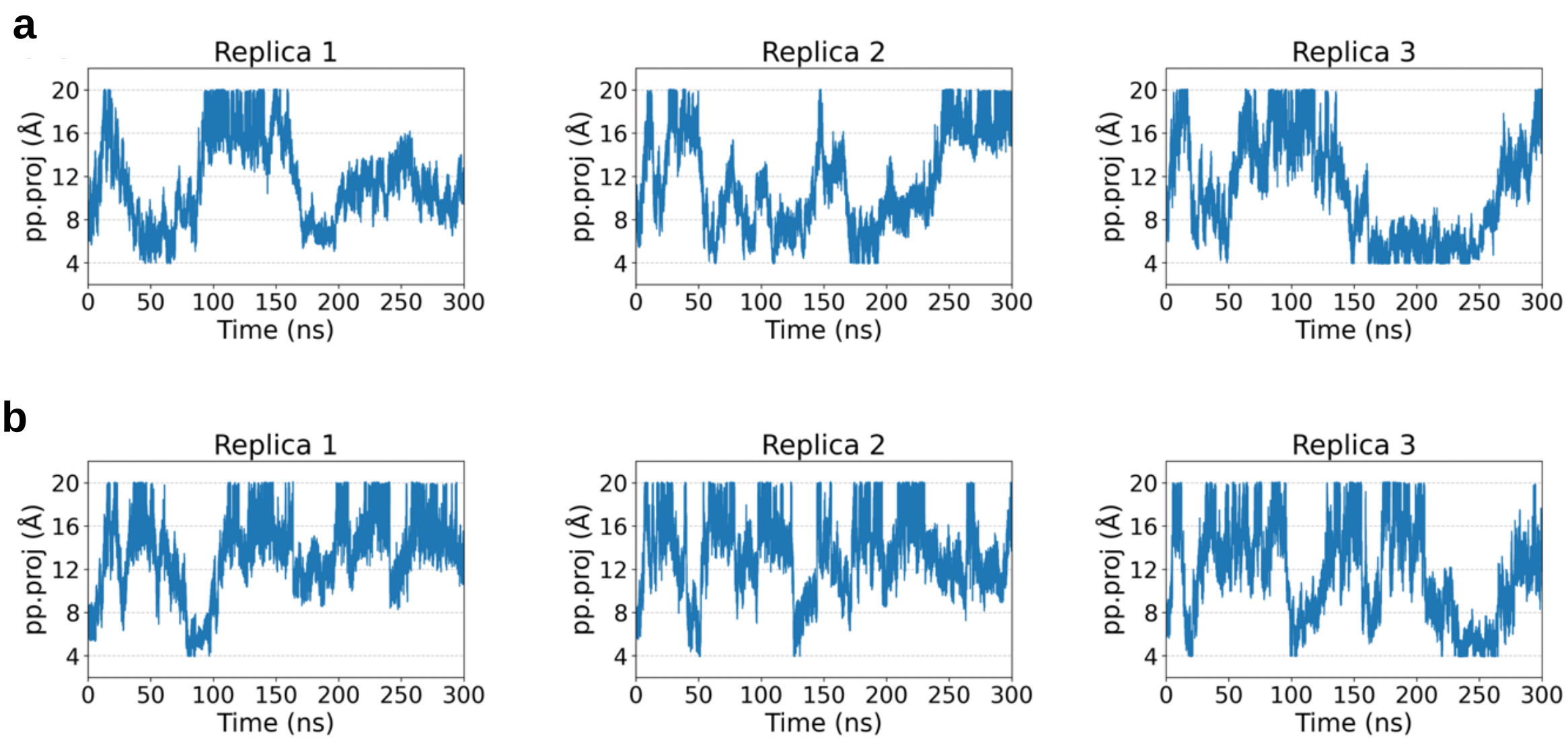

Supplementary Fig. 50. Time evolution of the funnel coordinate pp.proj along the funnel axis, during 300 ns funnel metadynamics simulations of the N30_L111I/G169A/V136L/V183I variant. Three independent replicas are shown for **a** (+)-borneol and **b** (−)-borneol, illustrating ligand movement along the binding pathway over time.

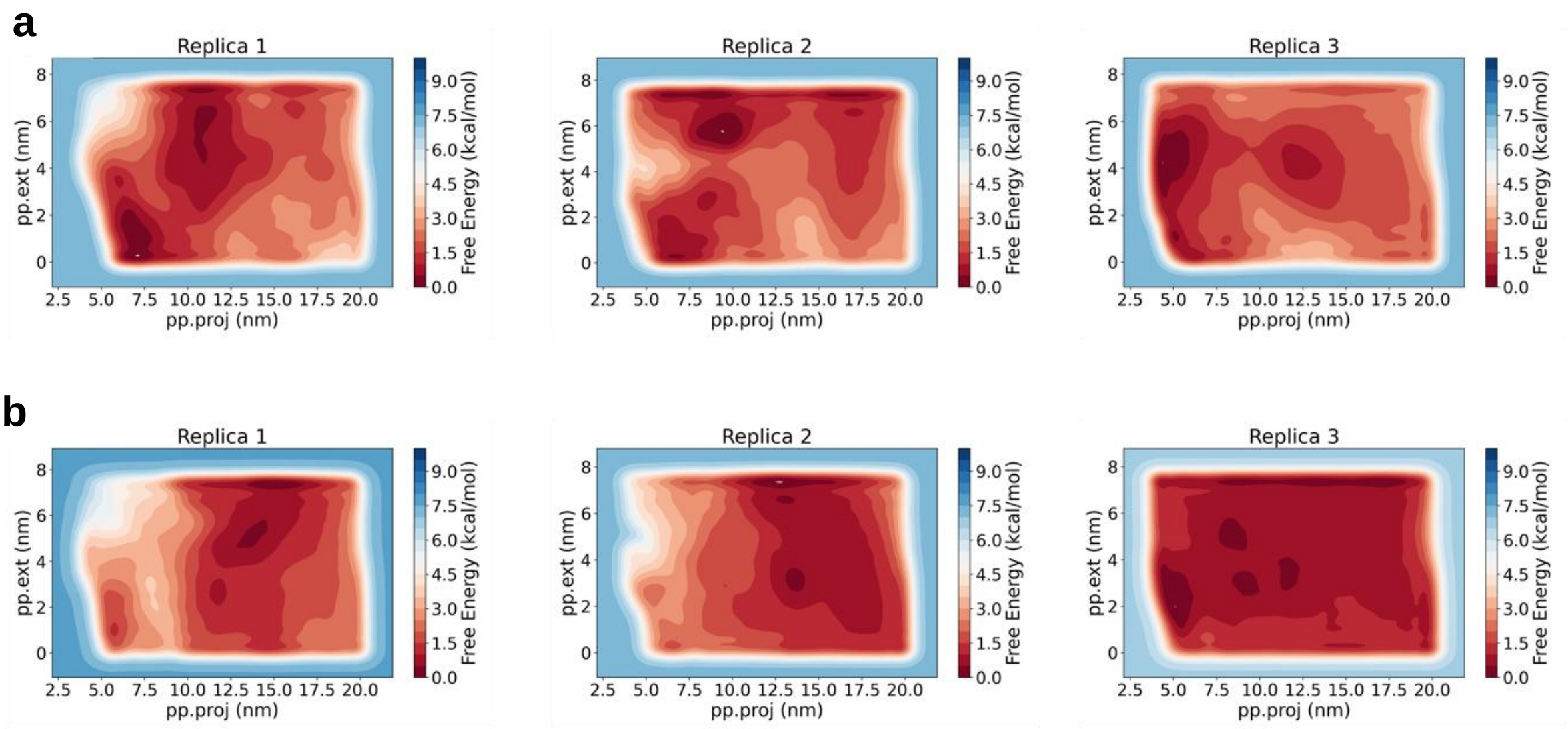

Supplementary Fig. 51. Two-dimensional free-energy surfaces projected onto the funnel collective variables pp.proj and pp.ext, obtained from funnel metadynamics simulations of the N30_L111I/G169A/V136L/V183I variant. Three independent replicas are shown for **a** (+)-borneol and **b** (−)-borneol.

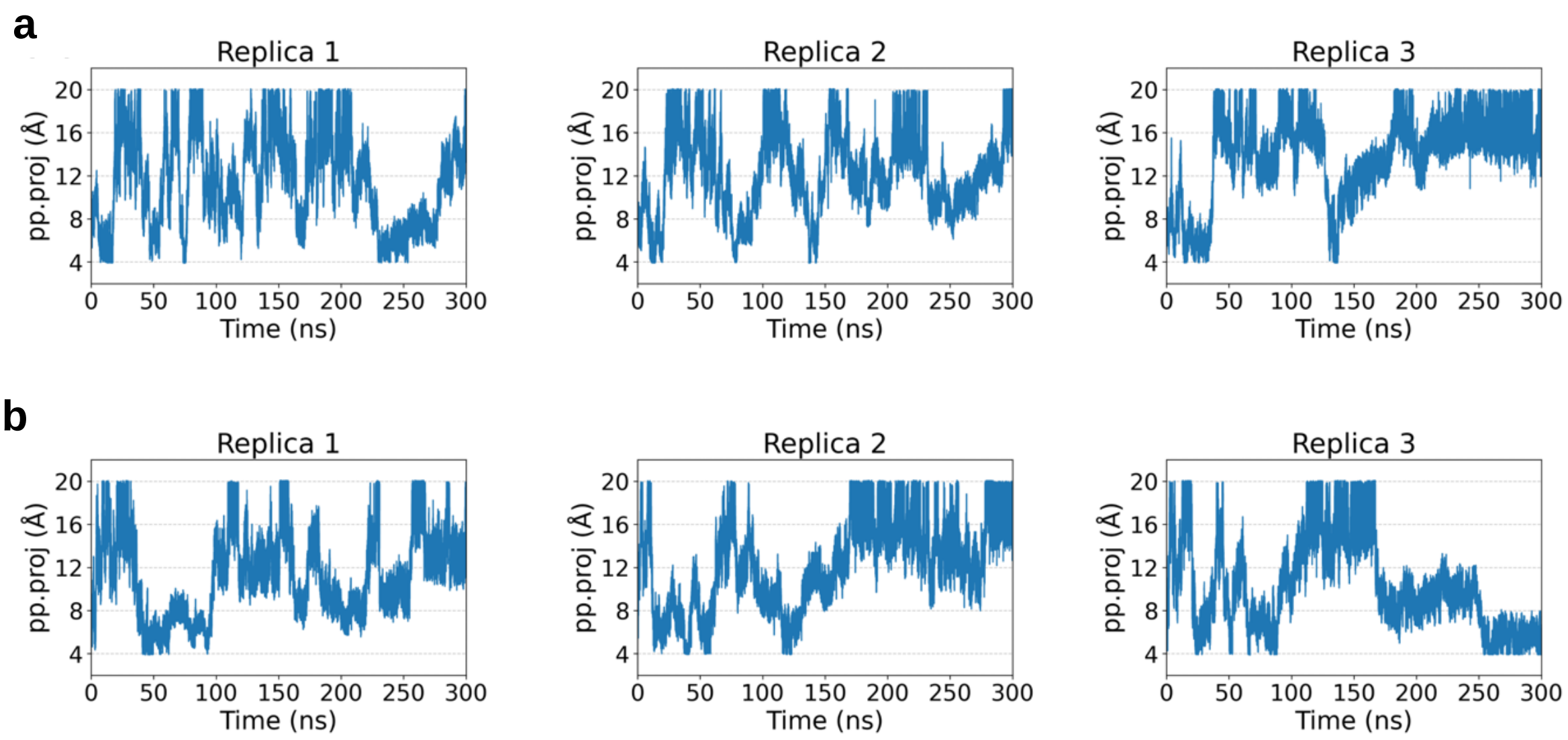

Supplementary Fig. 52. Time evolution of the funnel coordinate pp.proj along the funnel axis, during 300 ns funnel metadynamics simulations of the N6 variant. Three independent replicas are shown for **a** (+)-borneol and **b** (−)-borneol, illustrating ligand movement along the binding pathway over time.

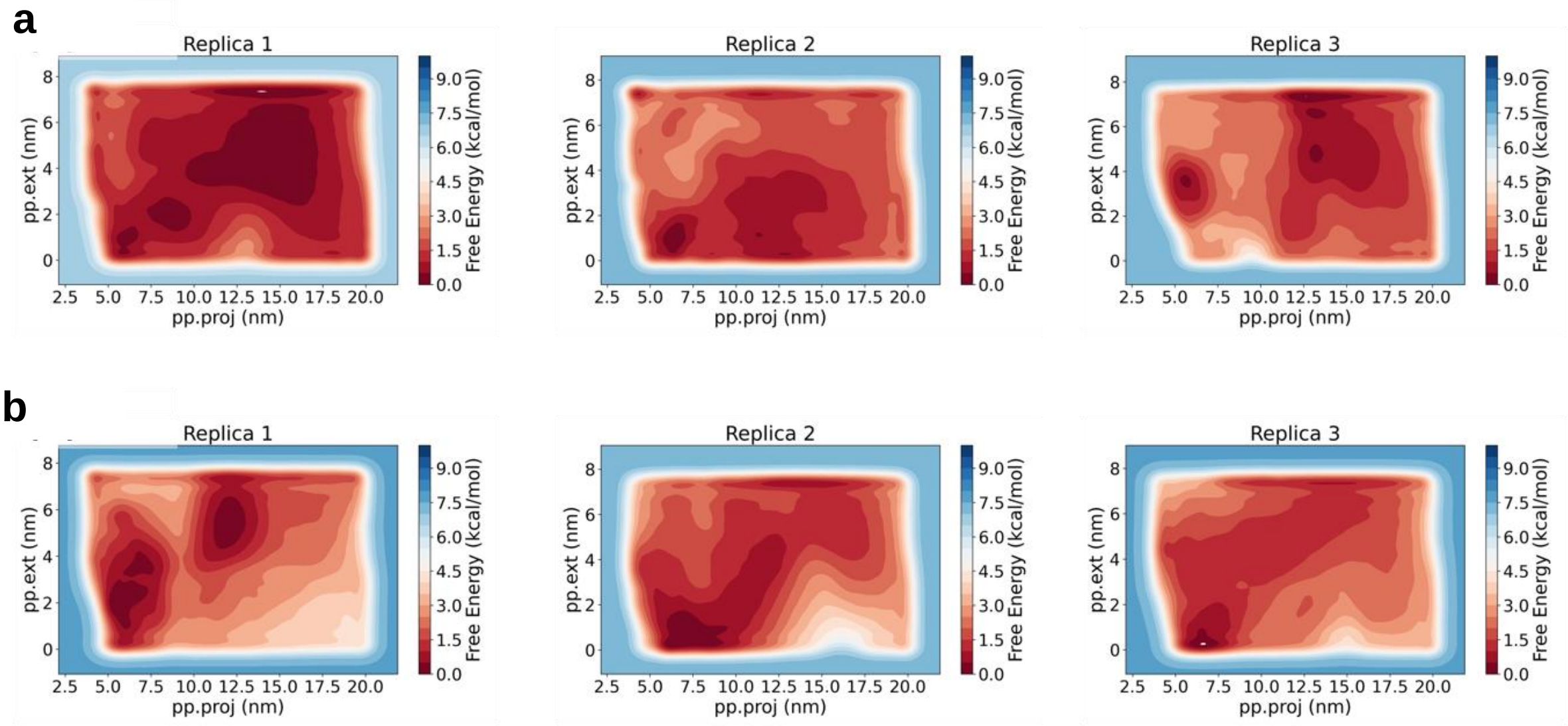

Supplementary Figure 53. Two-dimensional free-energy surfaces projected onto the funnel collective variables pp.proj and pp.ext, obtained from funnel metadynamics simulations of the N6 variant. Three independent replicas are shown for **a** (+)-borneol and **b** (−)-borneol.

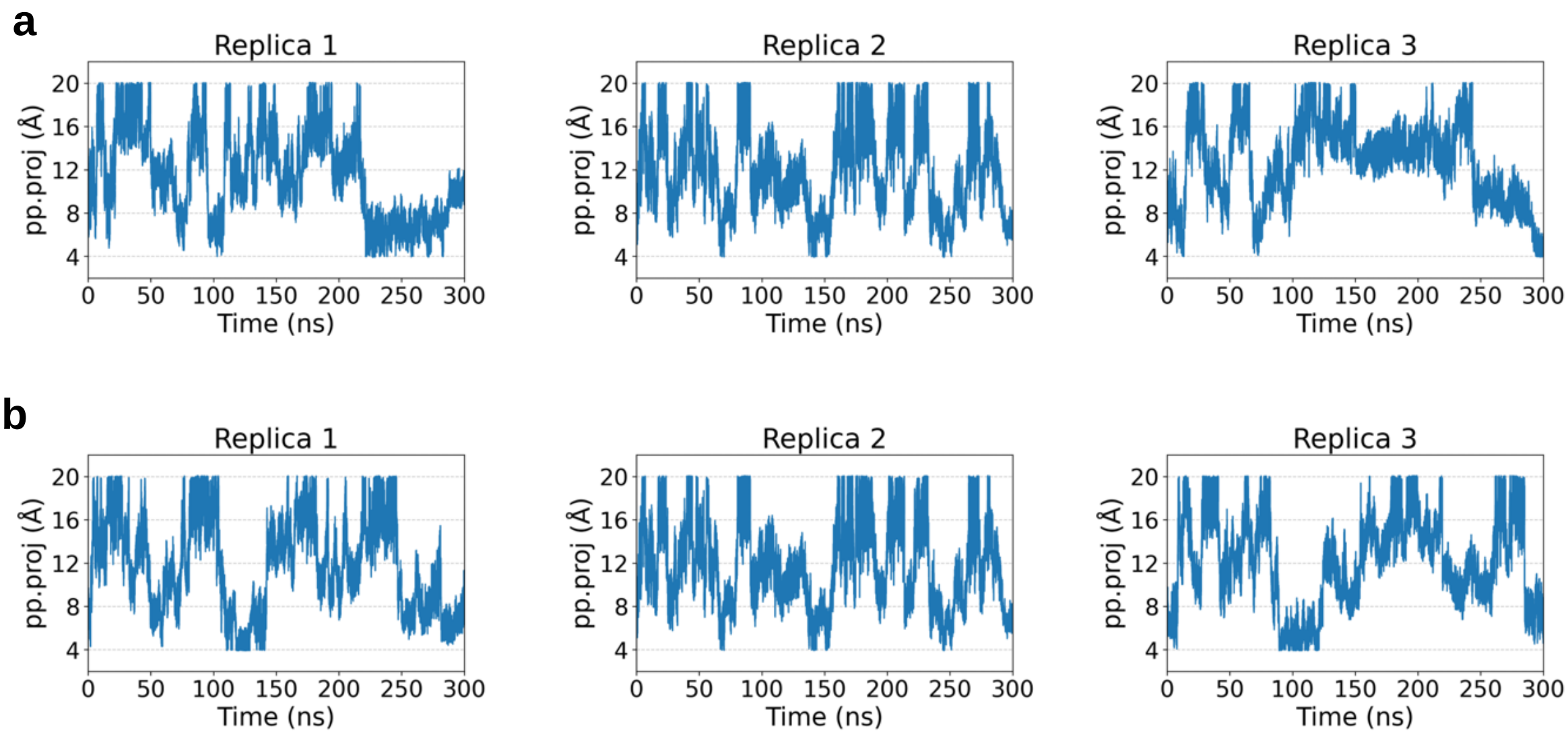

Supplementary Fig. 54. Time evolution of the funnel coordinate pp.proj along the funnel axis, during 300 ns funnel metadynamics simulations of the N32 variant. Three independent replicas are shown for **a** (+)-borneol and **b** (−)-borneol, illustrating ligand movement along the binding pathway over time.

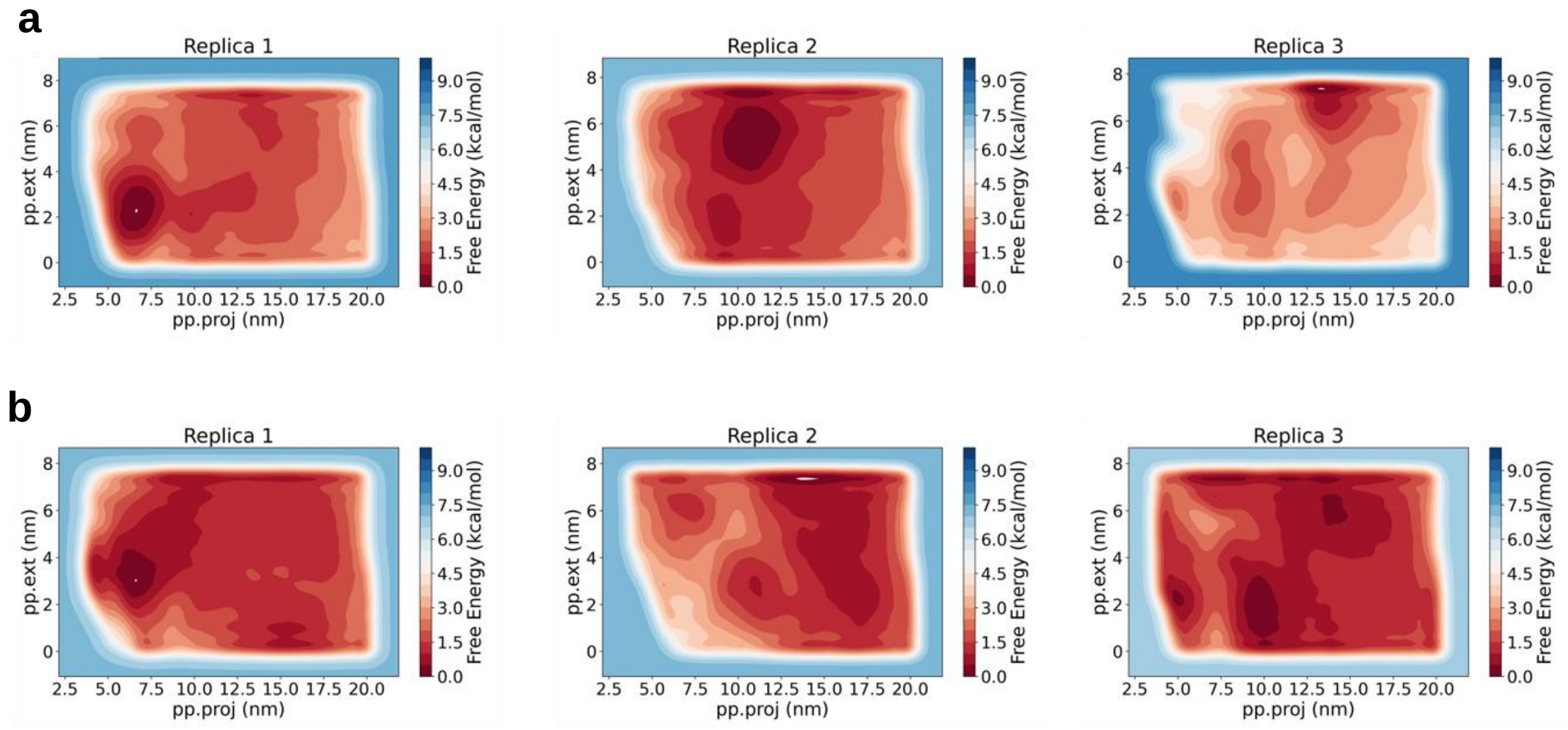

Supplementary Fig. 55. Two-dimensional free-energy surfaces projected onto the funnel collective variables pp.proj and pp.ext, obtained from funnel metadynamics simulations of the N32 variant. Three independent replicas are shown for **a** (+)-borneol and **b** (−)-borneol.

SDS-PAGE

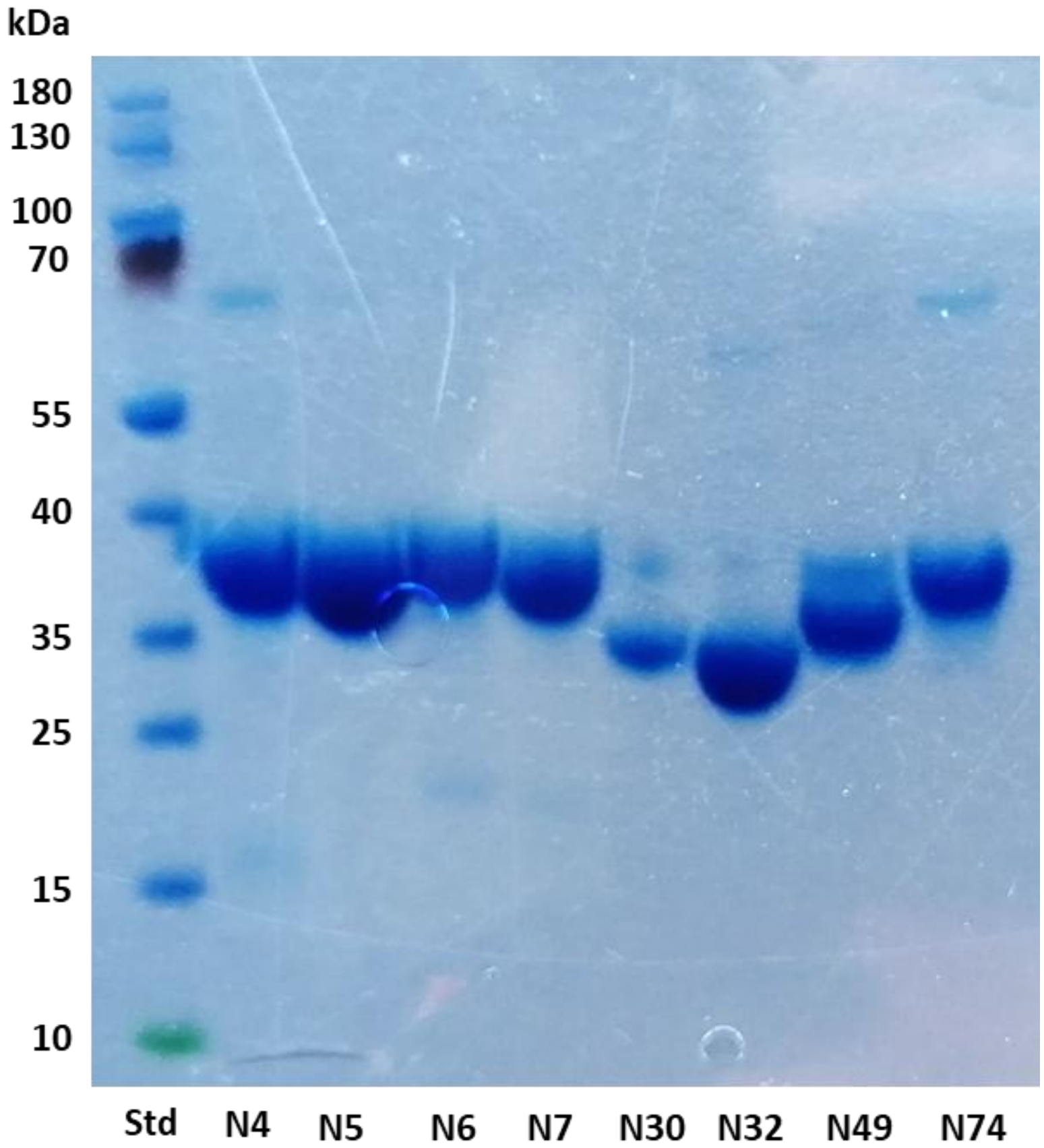

Supplementary Fig. 56. SDS-PAGE of BDH ancestors. Expression from pET28a(+) in *E. coli* BL21(DE3) at 28°C with 1 mM IPTG, overnight. Purified fractions after His-tag affinity chromatography are shown. 1 mg/mL protein applied to the SDS-PAGE. BDH ancestors are found between 25-40 kDa. PageRuler Prestained (ThermoScientific) was applied as standard (Std). The pictures of the uncropped gels are shown on page S77-78.

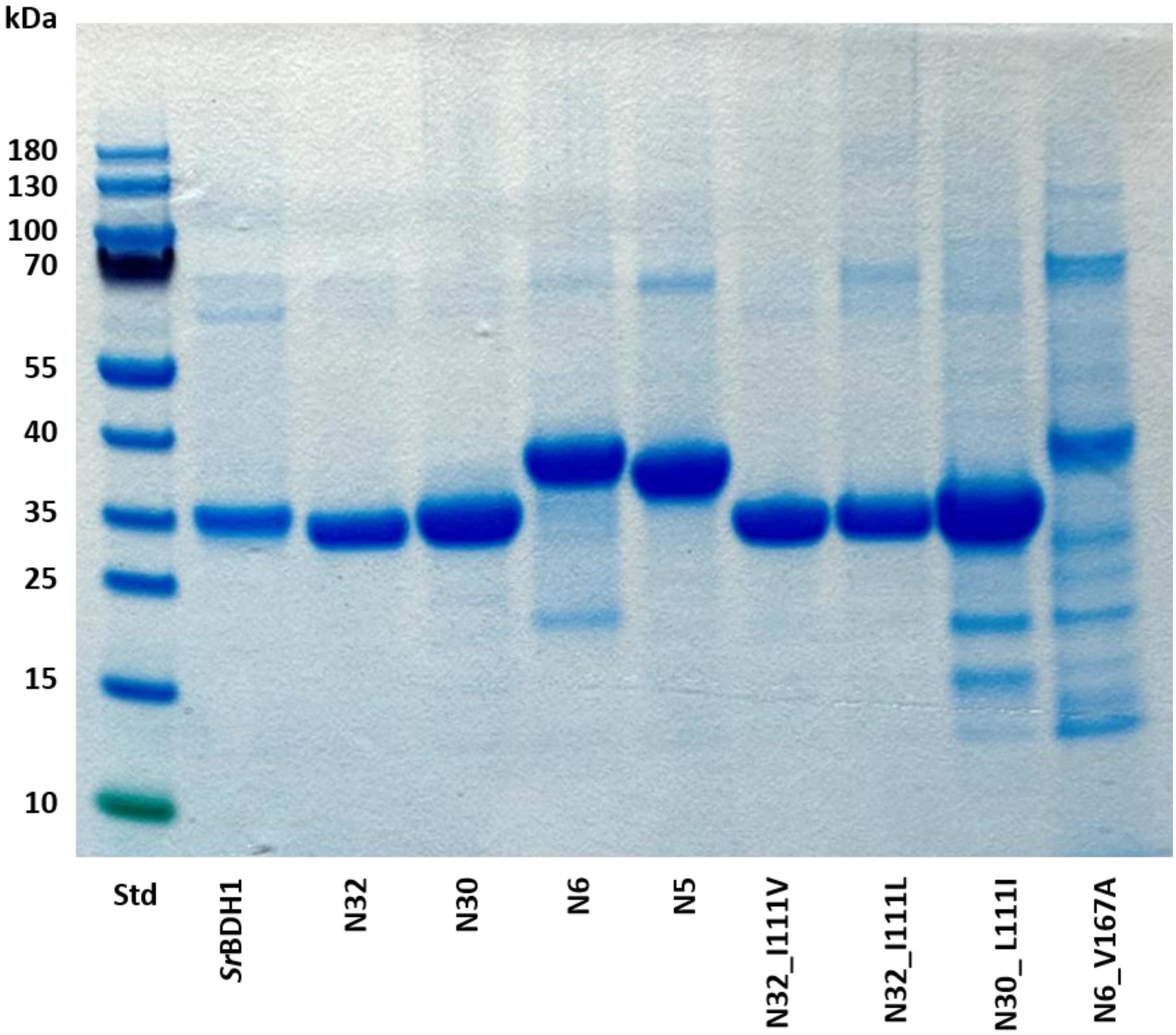

Supplementary Fig. 57. SDS-PAGE of BDH ancestors. Expression from pET28a(+) in *E. coli* BL21(DE3) at 28°C with 1 mM IPTG, overnight. Purified fractions after His-tag affinity chromatography are shown. 1mg/mL protein applied to the SDS-PAGE. BDH ancestors are found between 25-40 kDa. PageRuler Prestained (ThermoScientific) was applied as standard (Std). The pictures of the uncropped gels are shown on page S77-78.

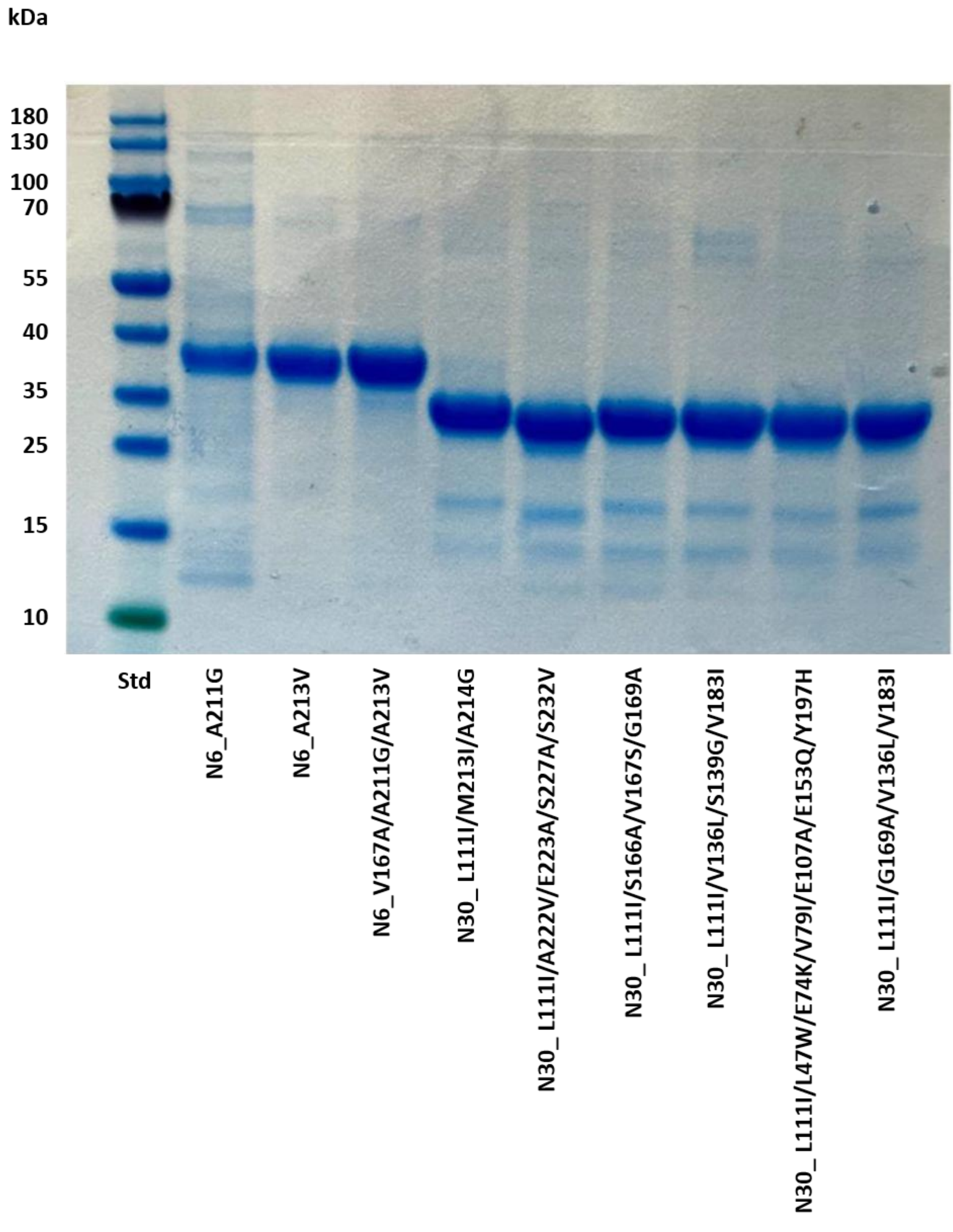

Supplementary Fig. 58. SDS-PAGE of BDH variants. Expression from pET28a(+) in *E. coli* BL21(DE3) at 28°C with 1 mM IPTG, overnight. Purified fractions after His-tag affinity chromatography are shown. 1mg/mL protein applied to the SDS-PAGE. BDH ancestors are found between 25-40 kDa. PageRuler Prestained (ThermoScientific) was applied as standard (Std). The pictures of the uncropped gels are shown on page S77-78.

Structure determination

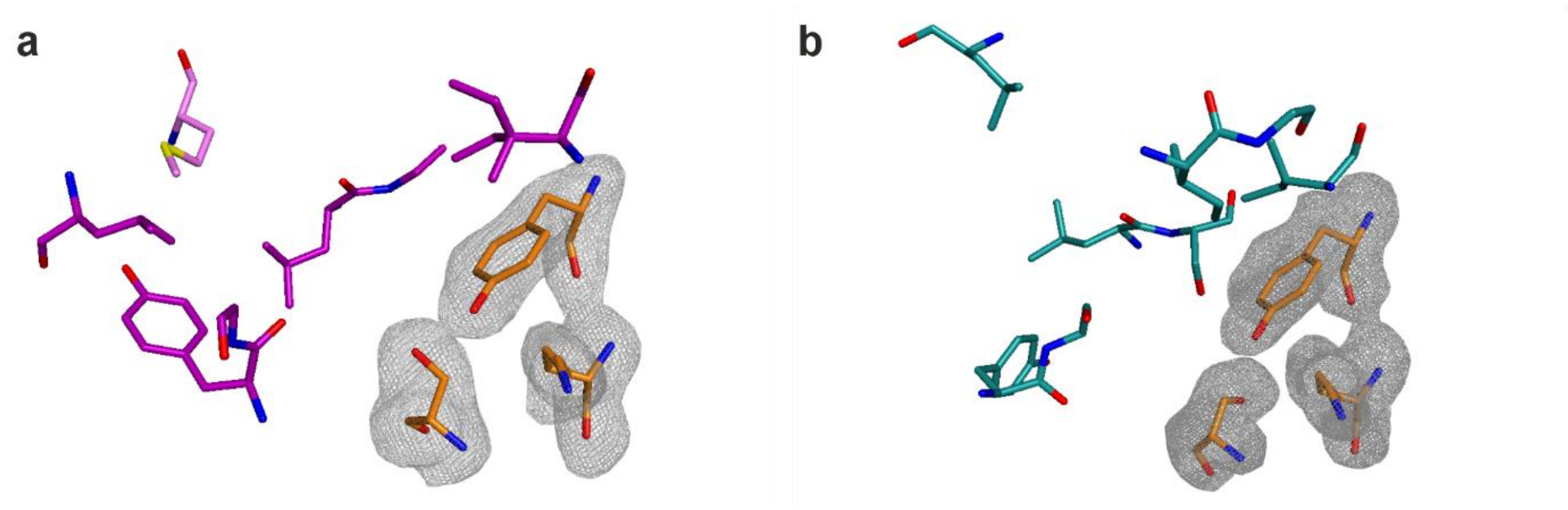

Supplementary Fig. 59. Polder maps for *Sr*BDH N32 and *Sr*BDH N39. a Polder map contoured at 3σ, showing electron density of the catalytic residues of N32. **b** Polder map contoured at 3σ of the catalytic residues of N39.

Supplementary Tables

Biochemical data ancestors

Supplementary Table 1. Enzyme yield mg protein per litre cultivation media. Expression from pET28a(+) in *E. coli* BL21(DE3) at 28 °C with 1 mM IPTG, overnight. Purified fractions after His-tag affinity chromatography.

**Variant**	**Yield [mg L^{-1} of culture]**	**E-value**
N4	21.3	50
N5	17.8	13
N6	45.9	10
N7	34.9	14
N30	60.4	12
N32	92.0	>200
N39	70.0	170
N48	n.d.	n.d.
N49	30.2	20
N74	77.8	69

Apparent K_M-values

Supplementary Table 2. Kinetic parameters of the ancestors as determined by applying Michaelis Menten kinetics calculated in Sigma Plot.

**variant**	**K_{app} [mM]**	**v_{max} [mU/mg]**	**K_i [mM]**
**N6**	0.01±0.01	49.81±4.40	2.45±0.90
**N30**	20.27±7.21	29.95±8.02	n.d.
**N32**	17.36±5.40	62.61±14.05	n.d.

Kinetic values of ancestral mutant variants

Supplementary Table 3. Specific activity and K_{app}-values determined by gas chromatography for BDH variants by oxidation of (+)-1-borneol enantiomers.

**Enzyme**	**Specific activity[mU/mg]** (+)-**1-borneol**	K_{app} **[mM]**
N32_I111L	36.6 ± 2.3	5.80 ± 2.5
N30_L111I	33.2 ± 1.4	18.11 ± 5.0
N30_L111I/G169A/V136L/V183I	15.3 ± 1.3	11.86 ± 1.4

Crystallographic data

Supplementary Table 4. Crystallographic data collection, refinement, and validation statistics.

	***Sr*BDH N32**	***Sr*BDH N39**
PDB entry	8R0C	8R0D
**Data collection**		
Wavelength [Å]	0.9184	0.9184
Temperature [K]	100	100
Space group	$P2_1$	$I222$
Unit Cell Parameters		
a, b, c [Å]	71.6 93.5 78.8	62.9 85.5 109.7
α, β, γ [°]	90.0 105.6 90.0	90.0 90.0 90.0
Resolution [Å][a]	50.00 - 1.99 (2.11 - 1.99)	50.00 - 1.85 (1.96 - 1.85)
Reflections [a]	470,800 (73,733)	138,314 (21,199)
Unique [a]	67,766 (10,487)	25,625 (4,084)
Completeness [%][a]	99.1 (95.3)	99.7 (99.1)
Multiplicity [a]	6.9 (7.0)	5.4 (5.2)
Data quality		
Intensity [I/σ(I)] [a]	10.30 (1.00)	16.8 (1.13)
R_{meas} [%][a,b]	0.150 (1.873)	0.168 (1.446)
$CC_{1/2}$ [a,c]	99.8 (46.8)	99.6 (49.7)
Wilson B value [Å^2]	40.0	29.3
**Refinement**		
Resolution [Å][a]	50.00 - 1.99 (2.04 - 1.99)	50.00 - 1.85 (1.92 - 1.85)
Reflections [a]		
Number	67,722 (4,039)	25,614 (2,788)
Test Set [%]	3.1	5.0
R_{work} [%][a]	0.174 (0.330)	0.175 (0.329)
R_{free} [%][a]	0.219 (0.375)	0.210 (0.404)
Asymmetric Unit	4	1
Protein residues chain A	247	251
Protein residues chain B	251	-
Protein residues chain C	260	-
Protein residues chain D	253	-

glycerol molecules	4	-
ethylene glycol molecules	-	7
formate molecules	-	1
water molecules	329	154
Temperature factors [Å^2]		
All Atoms	43.6	32.0
Chain A	45.9	31.2
Chain B	45.2	-
Chain C	42.0	-
Chain D	41.3	-
glycerol	52.2	-
ethylene glycol	-	40.9
formate	-	32.5
water molecules	42.6	40.6
RMSD from Target Geometry [d]		
Bond Lengths [Å]	0.007	0.010
Bond Angles [°]	0.888	1.015
**Validation Statistics**		
Ramachandran Plot [f]		
Residues in Allowed Regions [%]	2.6	3.2
Residues in Favoured Regions [%]	97.3	96.8
Ramachandran plot Z-score [f] (RMSD)		
whole	-0.90 (0.24)	-0.75 (0.45)
helix	-0.38 (0.20)	0.35 (0.39)
sheet	-0.74 (0.38)	-1.54 (0.68)
loop	-0.54 (0.31)	-1.11 (0.59)
MOLPROBITY Clashscore [f]	4.11	1.99
MOLPROBITY score [g]	1.50	1.29
Poor rotamers [%]	1.47	1.46
Cβ deviations [%]	0	0

[a] data for the highest resolution shell in parenthesis

[b] $R_{meas}(I) = \sum_h [N/(N-1)]^{1/2} \sum_i |I_{ih} - \langle I_h \rangle| / \sum_h \sum_i I_{ih}$, in which $\langle I_h \rangle$ is the mean intensity of symmetry-equivalent reflections h, I_{ih} is the intensity of a particular observation of h and N is the number of redundant observations of reflection h .[12]

[c] $CC_{1/2} = (\langle I^2 \rangle - \langle I \rangle^2) / (\langle I^2 \rangle - \langle I \rangle^2) + \sigma^2_\varepsilon$, in which σ^2_ε is the mean error within a half-dataset .[13]

[d] RMSD – root mean square deviation.

[e] calculated with PHENIX.[14]

[f] Clashscore is the number of serious steric overlaps (>0.4 Å) per 1,000 atoms

[g] calculated with MOLPROBITY .[15]

Supplementary Table 5. Structural comparison of the crystal structures of N32, N39, *So*BDH1, *Sr*BDH2. R.m.s.d. [Å] calculated with SSM[16]as implemented in COOT.

	**N32**	**N39**	***So*BDH1**	***Sr*BDH2**
**N32**				
**N39**	1.19			
***So*BDH1**	1.35	1.22		
***Sr*BDH2**	0.97	0.82	1.34	

Supplementary Table 6. R.m.s.d. [Å] for AlphaFold3[17] models.

	**N6**	**N30**	**N32**	***Sr*BDH1**
**N6**	x	0.246	0.249	0.308
**N30**		x	0.15	0.322
**N32**			x	0.284
***Sr*BDH1**				x

Docking and ML/MM data

Supplementary Table 7. Relevant catalytic distances d_1 and d_2 [Å], obtained from Chai-1[121] generated models of different *Sr*BDH1 variants in complex with (+)- and (−)-borneol.

**system**	**(+)-borneol**		**(−)-borneol**	
	d_1	d_2	d_1	d_2
**wild-type**	2.1	3.3	2.8	3.2
**N32_I111L**	2.8	4.3	3.0	5.3
**N30_L111I**	5.4	4.1	2.6	4.3
**N30_L111I/G169A/V136L/V183I**	4.5	4.2	3.1	4.5
**N6**	2.8	3.4	2.7	3.2
**N32**	2.6	4.5	3.5	2.7

Supplementary Table 8. Summary of funnel metadynamics binding free energies for wild-type and engineered *Sr*BDH1 variants in complex with (+) and (−)-borneol.

System	ΔG (+) borneol	ΔG (−) borneol	ΔΔG	ΔΔG / $\sigma_{\Delta\Delta G}$	Assessment[a]
**Wildtype**	-5.1 ± 0.5	-3.6 ± 1.0	-1.5 ± 1.1	1.4	Not significant
**N32_I111L**	-2.2 ± 0.6	-2.1 ± 1.8	-0.1 ± 1.9	0.1	Not significant
**N30_L111I**	-2.0 ± 0.7	-3.7 ±1.7	+1.7 ± 1.8	0.9	Weak trend
**N30_L111I/G169A/ V136L/V183I**	-4.9 ± 1.3	-1.7 ± 0.6	-3.2 ± 1.4	2.3	Meaningful
**N6**	-2.1 ± 1.3	-5.1 ± 1.1	+3.0 ± 1.7	1.8	Meaningful
**N32**	-5.3 ± 0.6	-2.4 ± 1.1	-2.9 ± 1.3	2.2	Meaningful

[a] Reported values correspond to the calculated standard binding free energies (ΔG_{calc}), obtained by averaging the final 10 ns of the reconstructed free-energy profiles from each simulation. Values are reported as mean ± standard deviation across three independent 300 ns replicas, with uncertainties estimated using block analysis to account for time correlation in the metadynamics trajectories. Enantioselectivity was quantified as the free-energy difference between enantiomers (ΔΔG = ΔG(+)-borneol - ΔG(−)-borneol), and the associated uncertainty was obtained by propagation of errors assuming independent uncertainties for each enantiomer. The ratio $\Delta\Delta G/\sigma_{\Delta\Delta G}$ is reported as a dimensionless signal-to-noise metric to assess whether the observed enantioselective differences exceed statistical uncertainty. Systems with $\Delta\Delta G/\sigma_{\Delta\Delta G}$ values approaching or exceeding 2 were classified as statistically meaningful, while lower values indicate weak or statistically unresolved trends.

Structural comparisons

Supplementary Table 9. Structural comparison of the crystal structures of N32, N39, *So*BDH1, *Sr*BDH2. Sequence identity (%) calculated with SSM as implemented in COOT.

**Sequence identity (%)**	**N32**	**N39**	***So*BDH1**	***Sr*BDH2**
**N32**				
**N39**	86.87			
***So*BDH1**	54.10	53.73		
***Sr*BDH2**	67.67	68.37	50.94	

Isoborneol conversion

Supplementary Table 10. Overview of biochemical data on 1-isoborneol oxidation of the second mutant variant set.[a]

**construct**	***r*-1-isobor conv. [mU/mg]**
N30_L111I/N30_L111I13I/A214G	40.57 ± 22.60
N30_L111I/A222V/E223A/S227A/S232V	13.20 ± 0.68
N30_L111I/S166A/V167S/G169A	9.56 ± 2.99
N30_L111I/V136L/S139G/V183I	8.95 ± 1.63
N30_L111I/L47W/E74K/V79I/E107A/E153Q/Y197H	18.48 ± 4.03
N30_L111I/V136L/G169A/V183I	43.74 ± 2.80
SrBDH1 V111L	2.73 ± 0.34
*Sr*BDH1	68.29 ± 16.40
N32 L111V	40.38 ± 12.65
N30	n.d.
N32	n.d.
N32_I111L	109.52 ± 38.5
N30_L111I	23.65 ± 8.58

[a] Since the mutants displayed a higher selectivity toward the 1-borneol enantiomers, selectivity toward the racemic diastereomer 1-isoborneol was investigated. All variants just as the wildtype *Sr*BDH1 only convert (+)-1-isoborneol and do not accept the enantiomer (−)-1-isoborneol as substrate. Conversion rates toward the substrate, however, vary between the mutants. N32_I111L shows 1.6-fold increased activity, while other variants show up to 25-fold decreased activity compared to *Sr*BDH1. Here *SrBDH1* V97L nearly loses the capacity to convert the diastereomer, thus establishing it as the most stereospecific variant found in this study.

Supplementary Data

Sequences of ancestral proteins

>N4_0.25

MASSSSLSSSAKRLEGKVALITGGASGIGECTARLFAKHGAKVVIADIQDDLGQAVCEDLGSESASYVHCDVTIESDVENAVDFA
VSKYGKLDIMFNNAGILDPPKPSILDNEKSDFERVLSVNVTGVFLGMKHAARVMIPARSGSIISTASVASVIGGVASHAYTCSKHA
VVGLTKNVAVELGQYGIRVNCVSPYAVATPMARNFLKLDEEAVENMVSVYANLKGVVLKAEDVAEAALYLASDEAKYVSGHNLV
VDGGFTIVNPSFGMFKQPPNS

>N5_0.85

RLEGKVALITGGASGIGESTVRLFVKHGAKVVIADIQDDLGQAVCEDLGSTESVSYVHCDVTIESDVQNAVDFTVSKYGKLDIMFN
NAGILGPPNPSILDNDKSDFERVLSVNVTGVFLGMKHAARVMIPAKKGSIISTASVASVIGGLGPHAYTASKHAVVGLTKNVAVEL
GQYGIRVNCVSPYAVATPMARNALKVDEEAVENMVSASANLKGVVLKAEDVAEAALYLASDEAKYVSGHNLVVDGGFTSVNPS
LGMFR

>N6_0.95

MSSSSSSSPPAKRLEGKVAIITGGASGIGESTVRLFVQHGAKVVIADIQDDLGQAICEDLGSTENVSYVHCDVTNESDVQNLVDT
TVSKYGKLDIMFNNAGILGRPNSSILDTDKSDFERVLGVNVTGAFLGAKHAARVMIPAKKGCILFTASVASVIGGLGPHAYTASKH
AVVGLTKNLAVELGQYGIRVNCVSPYAVATPMARNALKVDEEAVENMISESANLKGVVLKAEDVAEAALYLASDEAKYVSGLNLV
VDGGFSTVNPSLTMAMKPPNS

>N7_0.84

MSGSSSRAPIAKRLEGKVAIITGGASGIGESTVRLFVQHGAKVVIADIQDDLGQSICKDLGSDENVSYVHCDVTNDSDVQNLVDTT
VSKYGKLDIMFNNAGISGNLNSSILDTDNEDFKRVFDVNVYGAFLGAKHAARVMIPAKKGCILFTSSVASVISGLGPHAYTASKHA
VVGLTKNLCVELGQYGIRVNCISPYAVATPMLRNAMKVDESAVENMISESANLKGVVLKAEDVAEAALYLASDESKYVSGLNLVV
DGGYSTINQSLTMAMKSLSS

>N30_0.94

KRLEGKVAIITGGASGIGASAVRLFLENGAKVVIADIQDDLGQAICDKLGENVSYVHCDVSNEDDIRNLVDTTVAKYGKLDIMFNNA
GILDRPYGSILDTEKSDLERVLGVNVVGSFLGAKHAARVMVPERKGCILFTASACSVIGGLGTHAYTASKHAVVGLMKNLAAELG
QYGIRVNCVSPYGVVTGMARGVSEVDAEQVESMLSESGNLKGAVLKVEDVAQAALYLASDEANYVSGLNLVVDGGFSVVNPSL
MMAL

>N32_0.88

KRLEGKVAIITGGASGIGASAVRLFWENGAKVVIADIQDDLGQAICDKLGKNVSYIHCDVSNEDDIRNLVDTTVAKYGKLDIMFNNA
GIIDRPYGSILDTEKSDLERVLGVNLVGGFLGAKHAARVMVPQRKGCILFTASACASIAGLGTHAYTASKHAIVGLMKNLAAELGQ
HGIRVNCVSPYGVVTGIGRGVSEVDVAQVEAMLSEVGNLKGAVLKVEDVAQAALYLASDEANYVSGLNLVVDGGFSVVNPSMM
MAL

>N39_0.75

SKRLEGKVAIITGGASGIGASTVQLFHENGAKVVIADIQDDLGQAIANKLGKNVCYIHCDVSNEDDIINLVDTTVAKYGKLDIMYNN
AGIIDRPFGSILDTTKSDLERVLGVNLVGAFLGAKHAARVMVPQKKGCILFTASACTAIAGLSTHAYAVSKYGIVGLAKNLAAELGQ
HGIRVNCVSPYGVVTGIGGVSEVDVAVVEAMLSEVGNLKGQILKAEGVAKAALYLASDEANYVSGLNLVVDGGFSVVNPTMMKA
LNPPES

>N48_0.57RLEGKVALITGGASGIGESTVRLFVKHGAKVVIADIQDDLGQALCETLGSTSSVSYVHCDVTIESDVQNAVDFTVSKY
GKLDIMFNNAGILGPPNPSILDFDLSDFERVLSVNVKGVFLGMKHAARVMIPAKKGSIISTASVASVIGGLGPHAYTASKHAVVGLT
KNVAVELGQYGIRVNCVSPYAVATPMALNALKVDEEAVENFVGASANLKGVVLKAEDVAEAALYLASDEAKYVSGHNLVVDGGF
TSSNHSLGMFR

>N49_0.88

MAATSSSRSSLPAQRLLGKVALVTGGASGIGESIVRLFYKHGAKVCIADIQDDLGQRLCETLGGNSSVSFCHCDVTIEADVQRAV
DFTVDKFGSLDIMVNNAGISGPPCPDIRDFDLSVFERVFDVNVKGVFLGMKHAARVMIPAKKGSIISTCSVASVIGGIGPHAYTGS
KHAVLGLTKNVAAELGKHGIRVNCVSPYAVATSLALAHLPEDERTEDALVGFRNFVGANANLQGVELTAEDVANAVVFLASDEA
RYVSGANLMVDGGFTSSNHSLRVFR

>N74_0.93

MASSSLLSAVAKRLEGKVALITGGASGIGECTARLFSKHGAKVVIADIQDDLGQSVCEDLGSESASFVHCDVTKESDVENAVDFA
VSKYGKLDIMFNNAGIVDEPKPSILDNEKSDFERVLSVNLTGVFLGTKHAARVMIPARSGSIISTASIASVIGGVASHAYTCSKHGV
VGLTKNAAVELGQYGIRVNCVSPYAVATPMARNFLKLDEEAVEGVVSVYANLKGVVLKAEDVAEAALYLASDEAKYVSGHNLVV
DGGFTIVNPSFGMF

Equations

Supplementary Equation S1. The ratio of the activity ratios of the singular enantiomers over the competitive substrates in relation to each other, mirror the selectivity of the enantiomers over each other. E = enantiomeric ratio. (S) or (R) = activity of enantiomers. reference = activity of competitive substrate.

$$E = \frac{(S)}{(R)} selectivity = \frac{\frac{(S)}{reference} selectivity}{\frac{(R)}{reference} selectivity}$$

Supplementary Equation S2 Michaelis Menten equation

$$v = \frac{v_{max} * [S]}{K_m + [S]}$$

Supplementary Equation S3 Michalis Menten Inhibition Model competitive inhibition

$$v = \frac{v_{max} * [S]}{K_m + [S] * (1 + \frac{[S]}{K_i})}$$

Supplementary Equation S4. Enantiomeric excess (ee) of most abundant substrate (S).

$$ee_S = |\frac{[S^R] - [S^S]}{[S^R] + [S^S]}|$$

Supplementary Equation S5. Enantiomeric excess (ee) of most abundant product (P).

$$ee_P = |\frac{[P^R] - [P^S]}{[P^R] + [P^S]}|$$

Supplementary Equation S6. E-value formular for irreversible reactions.

$$E = \frac{\ln[\frac{1 - ee_S}{1 + \frac{ee_S}{ee_P}}]}{\ln[\frac{1 + ee_S}{1 + \frac{ee_S}{ee_P}}]}$$

Supplementary Equation S7. Apparent E-value E_{app} given as the ratio of specific activities of the preferred enantiomer over the other.

$$E_{app} = \frac{v_R}{v_S}$$

Supplementary References

1. Chánique, A. M. *et al.* A Structural View on the Stereospecificity of Plant Borneol-Type Dehydrogenases. *ChemCatChem* **13**, 2262–2277 (2021).

2. Klett, J. *et al.* MM-ISMSA: An Ultrafast and Accurate Scoring Function for Protein–Protein Docking. *J. Chem. Theory Comput.* **8**, 3395–3408 (2012).

3. Semelak, J. A. *et al.* Advancing Multiscale Molecular Modeling with Machine Learning-Derived Electrostatics. *J. Chem. Theory Comput.* **21**, 5194–5207 (2025).

4. Gao, X., Ramezanghorbani, F., Isayev, O., Smith, J. S. & Roitberg, A. E. TorchANI: A Free and Open Source PyTorch-Based Deep Learning Implementation of the ANI Neural Network Potentials. *J. Chem. Inf. Model.* **60**, 3408–3415 (2020).

5. Pickering, I., Xue, J., Huddleston, K., Terrel, N. & Roitberg, A. E. TorchANI 2.0: An Extensible, High-Performance Library for the Design, Training, and Use of NN-IPs. *J. Chem. Inf. Model.* **65**, 11656–11671 (2025).

6. Pickering, I., Semelak, J. A., Xue, J. & Roitberg, A. E. TorchANI-Amber: Bridging Neural Network Potentials and Classical Biomolecular Simulations. *J. Phys. Chem. B* **129**, 11927–11938 (2025).

7. Karrenbrock, M. *et al.* Absolute Binding Free Energies with OneOPES. *J. Phys. Chem. Lett.* **15**, 9871–9880 (2024).

8. Barducci, A., Bonomi, M. & Parrinello, M. Metadynamics. *WIREs Computational Molecular Science* **1**, 826–843 (2011).

9. Barducci, A., Bussi, G. & Parrinello, M. Well-Tempered Metadynamics: A Smoothly Converging and Tunable Free-Energy Method. *Phys. Rev. Lett.* **100**, 20603 (2008).

10. Limongelli, V., Bonomi, M. & Parrinello, M. Funnel metadynamics as accurate binding free-energy method. *Proceedings of the National Academy of Sciences* **110**, 6358–6363 (2013).

11. Raniolo, S. & Limongelli, V. Ligand binding free-energy calculations with funnel metadynamics. *Nat. Protoc.* **15**, 2837–2866 (2020).

12. Diederichs, K. & Karplus, P. A. Improved R-factors for diffraction data analysis in macromolecular crystallography. *Nature Structural Biology 1997 4:4* **4**, 269–275 (1997).

13. Karplus, P. A. & Diederichs, K. Linking crystallographic model and data quality. *Science (1979).* **336**, 1030–1033 (2012).

14. Adams, P. D. *et al.* PHENIX: a comprehensive Python-based system for macromolecular structure solution. *Acta Crystallogr. D Biol. Crystallogr.* **66**, 213–221 (2010).

15. Williams, C. J. *et al.* MolProbity: More and better reference data for improved all-atom structure validation. *Protein Science* **27**, 293–315 (2018).

16. Krissinel, E. & Henrick, K. Secondary-structure matching (SSM), a new tool for fast protein structure alignment in three dimensions. *Acta Crystallogr. D Biol. Crystallogr.* **60**, 2256–2268 (2004).

17. Abramson, J. *et al.* Accurate structure prediction of biomolecular interactions with AlphaFold 3. *Nature 2024 630:8016* **630**, 493–500 (2024).

18. Boitreaud, J. *et al.* Chai-1: Decoding the molecular interactions of life. Preprint at https://doi.org/10.1101/2024.10.10.615955 (2024).

Source Data

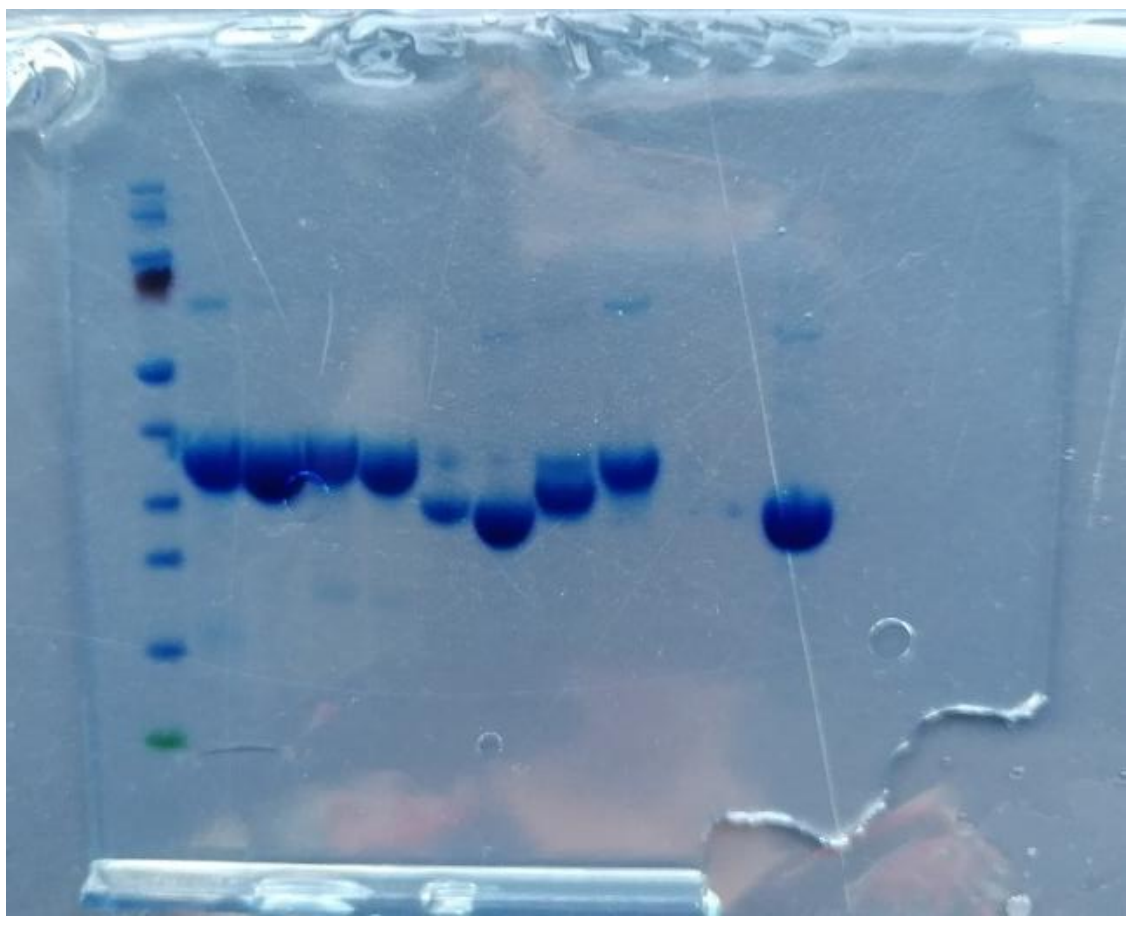

Source Data 1. Source data of Supplementary Figure S56.

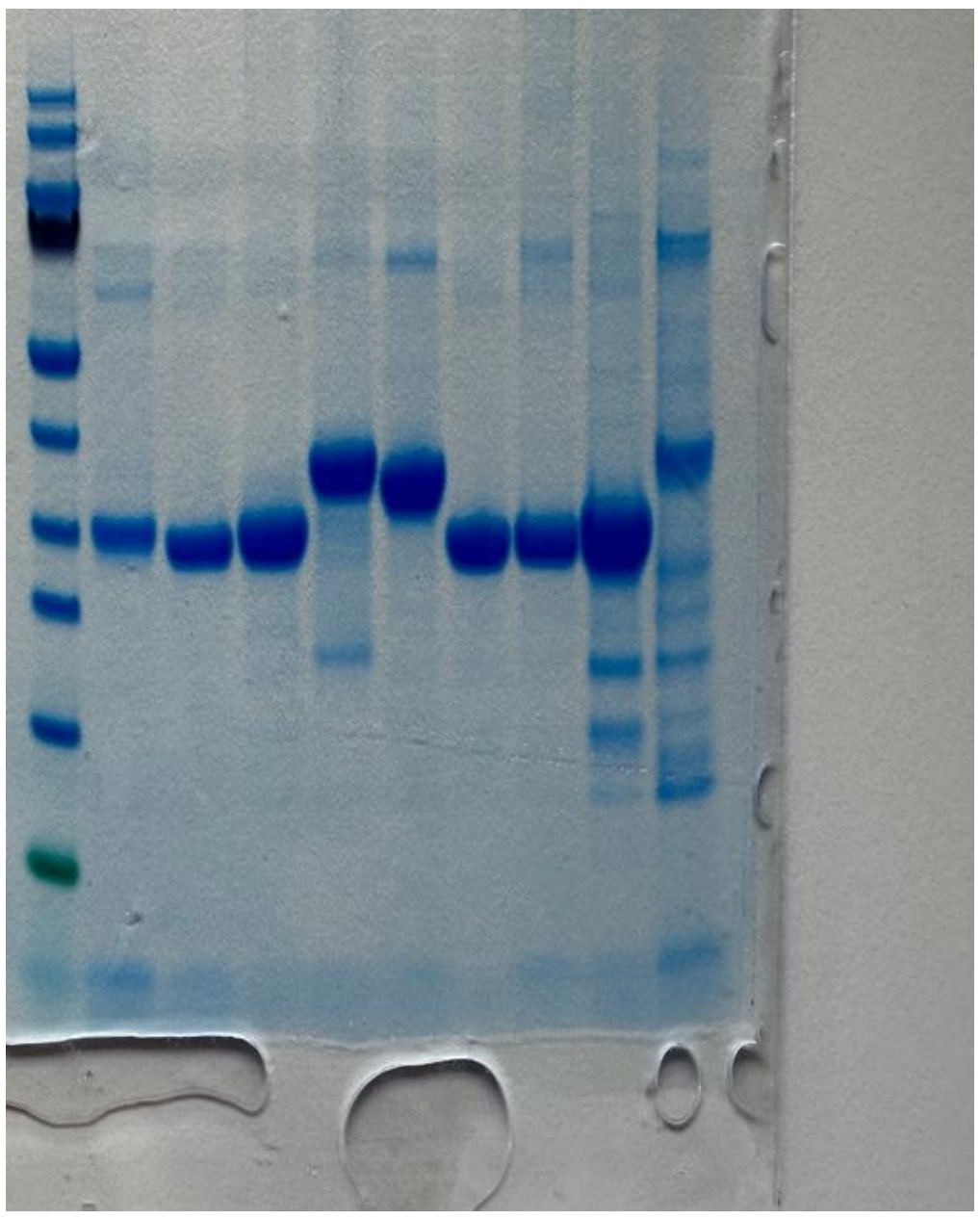

Source Data 2. Source data of Supplementary Figure S57.

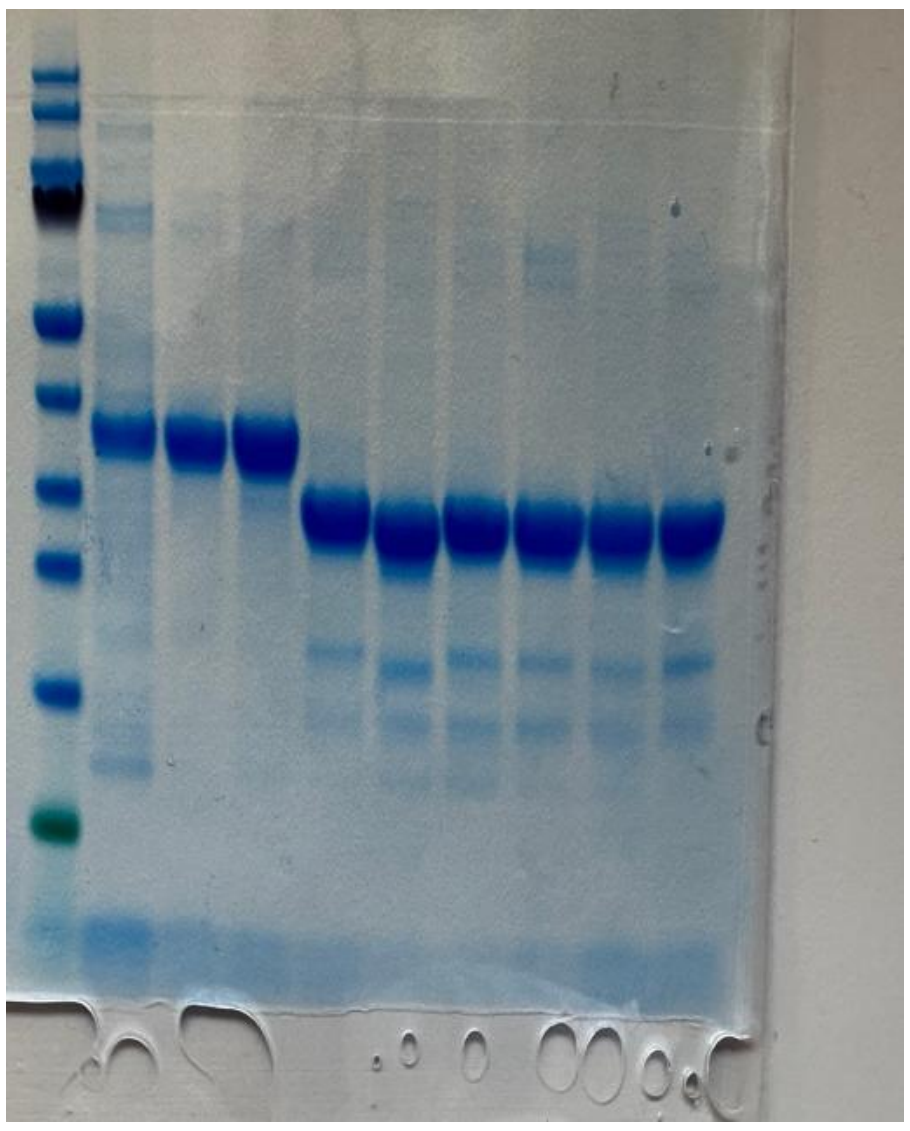

Source Data 3. Source data of Supplementary Figure S58.
