# Supplementary material for: Deciphering the evolutionary origin of the enantioselectivity of short-chain dehydrogenases from plants toward 1-borneol
Source: Nat Commun. 2026 Aug 14;17:9858. doi: 10.1038/s41467-026-76435-1 (PMC13578766; doi:10.1038/s41467-026-76435-1)
Supplement: Supplementary file 2 [file 41467_2026_76435_MOESM2_ESM.pdf]

nature portfolio

Corresponding author(s): Shina Caroline Lynn Kamerlin, Bernhard Loll, Robert Kourist

Last updated by author(s): Jul 1, 2026

Reporting Summary

Nature Portfolio wishes to improve the reproducibility of the work that we publish. This form provides structure for consistency and transparency in reporting. For further information on Nature Portfolio policies, see our Editorial Policies and the Editorial Policy Checklist.

Statistics

For all statistical analyses, confirm that the following items are present in the figure legend, table legend, main text, or Methods section.

n/a	Confirmed	
☐	☒	The exact sample size (*n*) for each experimental group/condition, given as a discrete number and unit of measurement
☐	☒	A statement on whether measurements were taken from distinct samples or whether the same sample was measured repeatedly
☒	☐	The statistical test(s) used AND whether they are one- or two-sided<br>*Only common tests should be described solely by name; describe more complex techniques in the Methods section.*
☒	☐	A description of all covariates tested
☒	☐	A description of any assumptions or corrections, such as tests of normality and adjustment for multiple comparisons
☐	☒	A full description of the statistical parameters including central tendency (e.g. means) or other basic estimates (e.g. regression coefficient) AND variation (e.g. standard deviation) or associated estimates of uncertainty (e.g. confidence intervals)
☒	☐	For null hypothesis testing, the test statistic (e.g. *F*, *t*, *r*) with confidence intervals, effect sizes, degrees of freedom and *P* value noted<br>*Give P values as exact values whenever suitable.*
☒	☐	For Bayesian analysis, information on the choice of priors and Markov chain Monte Carlo settings
☒	☐	For hierarchical and complex designs, identification of the appropriate level for tests and full reporting of outcomes
☒	☐	Estimates of effect sizes (e.g. Cohen's *d*, Pearson's *r*), indicating how they were calculated

Our web collection on statistics for biologists contains articles on many of the points above.

Software and code

Policy information about availability of computer code

Data collection	No additional software was used to collect data.
Data analysis	BLAST (https://blast.ncbi.nlm.nih.gov/), NEBBaseChanger (https://nebasechanger.neb.com/), Benchling (https://benchling.com/), ProbCons (http://probcons.stanford.edu/), Gblocks, PhyML, EVCouplings (https://v2.evcouplings.org/), GRASP (https://grasp.scmb.uq.edu.au/), SigmaPlot 14.0, PyMol 2.4.0 and 3.1.2, CHIMERAX (1.09.1), AlphaFold3, XDS, PHASER, PHENIX (1.19_4092), COOT (0.9.4), MolProbity (4.5.1), JCSG validation server (JCSG Quality Control Check v3.1), DSSP, Alscript, SWISS-MODEL webserver, Open Babel, AutoDock VINA, AmberTools, Amber 24, SHAKE., PLUMED 2.9

For manuscripts utilizing custom algorithms or software that are central to the research but not yet described in published literature, software must be made available to editors and reviewers. We strongly encourage code deposition in a community repository (e.g. GitHub). See the Nature Portfolio guidelines for submitting code & software for further information.

Data

Policy information about availability of data

All manuscripts must include a data availability statement. This statement should provide the following information, where applicable:

- Accession codes, unique identifiers, or web links for publicly available datasets
- A description of any restrictions on data availability
- For clinical datasets or third party data, please ensure that the statement adheres to our policy

The atomic coordinates have been deposited in the Protein Data Bank with the accession code 8R0C (SrBDH N32) (https://doi.org/10.2210/pdb8R0C/pdb) and 8R0D (SrBDH N39) (https://doi.org/10.2210/pdb8R0D/pdb). Diffraction raw images have been deposited at proteindiffraction.org: https://doi.org/10.18430/M38R0C (SrBDH N32) and https://doi.org/10.18430/m38r0d (SrBDH N39). Raw data of the biochemical analysis, structure predictions and simulation data have been deposited at Zenodo. The package contains chromatograms of three kinetic measurements, the extracted peak areas and calculations. Additional information such as nucleotide sequences are deposited at the same location. The data is located atThe atomic coordinates have been deposited in the Protein Data Bank with the accession code 8R0C (N32) and 8R0D (N39). Diffraction raw images have been deposited at proteindiffraction.org: 10.18430/M38R0C (N32) and 10.18430/M38R0D (N39). Raw data of the biochemical analysis, structure predictions and simulation data have been deposited at Zenodo. The package containis chromatograms of three kinetic measurements, the extracted peak areas and calculations. The data is located at DOI: 10.5281/zenodo.18925996 .
Previously published structural and sequence data used in this study are available under the following accession codes:
6ZYZ [http://doi.org/10.2210/pdb6ZYZ/pdb] (crystal structure of SrBDH1)
7O6P [http://doi.org/10.2210/pdb7O6P/pdb] (crystal structure of SoBDH2)
8R0D [http://doi.org/10.2210/pdb8R0D/pdb] (crystal structure of N39)
8R0C [http://doi.org/10.2210/pdb8R0C/pdb] (crystal structure of N32)
AFV30207.1 [https://www.ncbi.nlm.nih.gov/protein/AFV30207.1] (Lavandula intermedia borneol dehydrogenase, LiBDH)
ADK56099.1 [https://www.ncbi.nlm.nih.gov/protein/ADK56099.1] (Artemisia annua alcohol dehydrogenase 2, AaADH2)
ANJ65952.1 [https://www.ncbi.nlm.nih.gov/protein/ANJ65952.1] (Artemisia annua borneol dehydrogenase, AaBDH)
WP_032492645.1 [https://www.ncbi.nlm.nih.gov/protein/WP_032492645.1] (Pseudomonas sp. TCU-HL1 borneol dehydrogenase, PsBDH)
MT525100 [https://www.ncbi.nlm.nih.gov/nuccore/MT525100] (Salvia officinalis borneol-type dehydrogenase 1, SoBDH1)
MT525099 [https://www.ncbi.nlm.nih.gov/nuccore/MT525099] (Salvia officinalis borneol-type dehydrogenase 2, SoBDH2)
MT857224 [https://www.ncbi.nlm.nih.gov/nuccore/MT857224] (Salvia rosmarinus borneol-type dehydrogenase 1, SrBDH1)
1KP database [https://db.cngb.org/onekp/] (plant transcriptome database)

Research involving human participants, their data, or biological material

Policy information about studies with human participants or human data. See also policy information about sex, gender (identity/presentation), and sexual orientation and race, ethnicity and racism.

Reporting on sex and gender	No human data was included in this study.
Reporting on race, ethnicity, or other socially relevant groupings	No human data was included in this study.
Population characteristics	No human data was included in this study.
Recruitment	No human data was included in this study.
Ethics oversight	No human data was included in this study.

Note that full information on the approval of the study protocol must also be provided in the manuscript.

Field-specific reporting

Please select the one below that is the best fit for your research. If you are not sure, read the appropriate sections before making your selection.

☒ Life sciences ☐ Behavioural & social sciences ☐ Ecological, evolutionary & environmental sciences

For a reference copy of the document with all sections, see nature.com/documents/nr-reporting-summary-flat.pdf

Life sciences study design

All studies must disclose on these points even when the disclosure is negative.

Sample size	For activity assays three replicates were produced (n=3).
Data exclusions	No data was excluded.
Replication	Sample sizes were chosen based on standard practice in protein biochemistry and engineering studies. For most assays, measurements were performed in technical replicates (typically n = 3) to ensure assay precision and reproducibility for each protein sample.In some cases, experiments were repeated using independently prepared protein samples; however, independent biological replicates were not

systematically annotated across all experiments during the study, and therefore technical replicates are conservatively reported as the primary unit of replication where applicable.No formal statistical power calculations were performed prior to experimentation. The overall sample sizes are consistent with established practice in in vitro protein characterization studies, where technical triplicates are commonly used to assess measurement variability and support quantitative comparisons between conditions.

Randomization	Samples were not allocated into experimental groups using randomization, as this study involved in vitro characterization of purified proteins and protein variants that were deliberately designed and assigned to specific experimental conditions (e.g., mutants, constructs, or assay conditions) based on the aims of each experiment.
Blinding	Blinding was not relevant to this study. All experiments were based on biochemical assays, structural analyses, and computational simulations using predefined quantitative readouts and automated or instrument-based data acquisition and analysis. No subjective outcome assessment or investigator-dependent group allocation was involved.

Reporting for specific materials, systems and methods

We require information from authors about some types of materials, experimental systems and methods used in many studies. Here, indicate whether each material, system or method listed is relevant to your study. If you are not sure if a list item applies to your research, read the appropriate section before selecting a response.

Materials & experimental systems

n/a	Involved in the study
☒	☐ Antibodies
☒	☐ Eukaryotic cell lines
☒	☐ Palaeontology and archaeology
☒	☐ Animals and other organisms
☒	☐ Clinical data
☒	☐ Dual use research of concern
☒	☐ Plants

Methods

n/a	Involved in the study
☒	☐ ChIP-seq
☒	☐ Flow cytometry
☒	☐ MRI-based neuroimaging

Plants

Seed stocks	No plants were used in this study.
Novel plant genotypes	No plants were used in this study.
Authentication	No plants were used in this study.
