# Supplementary material for: Deciphering the evolutionary origin of the enantioselectivity of short-chain dehydrogenases from plants toward 1-borneol
Source: Nat Commun. 2026 Aug 14;17:9858. doi: 10.1038/s41467-026-76435-1 (PMC13578766; doi:10.1038/s41467-026-76435-1)
Supplement: Supplementary file 3 [file 41467_2026_76435_MOESM3_ESM.pdf]

nature portfolio

Peer Review File

Deciphering the evolutionary origin of the enantioselectivity of short- chain dehydrogenases from plants toward 1-borneol

Corresponding Author: Professor Robert Kourist

This file contains all reviewer reports in order by version, followed by all author rebuttals in order by version.

Attachments originally included by the reviewers as part of their assessment can be found at the end of this file.

Version 0:

Reviewer comments:

Reviewer #1

(Remarks to the Author)
How enantioselective enzymes have evolved from nonspecific precursors is an important question that has never been directly addressed by ancestral reconstruction. The authors have developed an ideal model system and sophisticated biochemical and structural methods for this goal. To bring to them bear on an evolutionary question, however, a robust phylogenetic framework is equally important. The authors provide only a scant description of phylogenetics, making it impossible for me to evaluate the plausibility of their evolutionary inferences. To justify their reconstructed evolutionary history, the following questions must be answered at minimum:

1. What exact model of sequence evolution was used, and how was it chosen?

The method section only says that the software PhyML was used for phylogenetics and the model JTT for ancestral reconstruction. I am sure there were more model features involved, such as the gamma-distributed among-site rate variation model and the invariant sites model; leaving these out typically results in a substantial model misfit. What was the exact model used? Why was JTT chosen among the many available substitution matrices? What statistical tests were performed to choose the best overall model of evolution?

2. How well were the phylogeny and ancestral sequences reconstructed?

The confidence of neither phylogenetic inference nor ancestral reconstruction is described. What are the statistical supports for the branching orders? Are they congruent with the established taxonomies? What are the posterior probabilities for the reconstructed ancestral sequences? Are the paper's major conclusions robust to alternate possible reconstructions?

3. How was the phylogeny rooted?

Incorrect rooting can lead to false inferences reversing the order of actual events. In the authors' phylogeny, for example, placing the root within the clade originating from N32 would flip the order of evolution—nonselective descendants evolving from selective ancestors. Rooting is not trivial for multicellular organisms when there are many paralogs as in the authors' case. The choice of the root must therefore be explained.

I regret that the excellent biochemical and structural data that the authors have collected cannot be used to make an evolutionary inference without first confirming that they are based on a robust phylogenetic inference.

On a matter of presentation, a circular phylogeny with long taxon names (Fig. 2) is virtually unreadable. Please collapse the phylogeny into major clades and show their branch lengths and supports. The full tree can be shown in a supplementary figure along with more detailed information on ancestral reconstruction. A good example is this paper: https://www.nature.com/articles/nature10724.

Reviewer #2

(Remarks to the Author)
Zuson et al. investigated the evolutionary emergence of enantioselectivity in plant borneol dehydrogenases (BDHs) and identified that one mutation in the active site, along with three peripheral mutations, was required to achieve high selectivity, as confirmed by site-directed mutagenesis. They further employed molecular simulations for mechanistic studies. These findings advance our understanding of BDH enantioselectivity and may guide the rational design of more efficient biocatalysts.
I have a few comments and questions:
1. Back-mutation of the peripheral residues on N32 is suggested to further validate the contributions of these peripheral mutations.
2. Kinetic data on all the mutants are needed to better elucidate how each mutation affects enantioselectivity.
3. The fonts in Figure 2A and 2B are too small to read.
4. In Table 1, the footnote cites [2], but this reference does not appear in the table.
5. In the supplementary dataset, what do the numbers following the "_" in the protein names represent (e.g., what does the "0" in N2_0 indicate)?
6. In Supplementary Figure 4, what do panels A, B, and C specifically represent?

Reviewer #3

(Remarks to the Author)
The paper by Kourist et al presents results obtained using a variety of tools with the aim to shed light on the enantioselectivity of plant enzymes toward 1-borneol. The issue is interesting and the employed approaches showed to provide synergistic contributions. My review is focused on the computational part of the paper. I have discussed this review with a PhD student in my group, Andresa Messias (andresa@qi.fcen.uba.ar)

Comments/suggestions:
I wonder if the fact that the ligand bound simulations are unstable in all cases is due to a parametrization flaw. One way to shed light on this issue could be to perform QM-MM (taking the ligand as the QM region) simulations which may allow for ligand polarization. Since this may be prohibitive computationally, a possible strategy could be to perform multi scale machine learning - molecular mechanics (ML-MM) calculations that have been shown to achieve near QM-MM quality at a low computational cost. Two possible implementations for this that are easily available are:
a) Semelak JA et al, Advancing Multiscale Molecular Modeling with Machine Learning-Derived Electrostatics. J Chem Theory Comput. 2025 May 27;21(10):5194-5207. doi: 10.1021/acs.jctc.4c01792
AmberTools25 with the interface Torchani-AMBER: https://github.com/roitberg-group/torchani-amber
b) emle-engine: A Flexible Electrostatic Machine Learning Embedding Package for Multiscale Molecular Dynamics Simulations
Zinovjev, K., et al Journal of Chemical Theory and Computation 2024 20 (11), 4514-4522 DOI: 10.1021/acs.jctc.4c00248
2) Binding free energies should be provided for the funnel meta dynamics simulations, in order to provide a more quantitative measurement of protein-ligand interactions. See for example Raniolo, S., Limongelli, V. Ligand binding free-energy calculations with funnel metadynamics. Nat Protoc 15, 2837–2866 (2020). https://doi.org/10.1038/s41596-020-0342-4.

Reviewer #4

(Remarks to the Author)
Review attached.

Version 1:

Reviewer comments:

Reviewer #1

(Remarks to the Author)
I thank the authors for addressing all of the concerns I had raised in the initial review. I offer just two suggestions regarding the phylogeny on Fig. 2; the authors are free to incorporate or ignore them, and I ask for no further responses.

First, I would explain in the main text that the phylogeny is rooted by taking the two bacterial genes as outgroups.

Second, line 145 states that ten ancestral proteins were biochemically characterized. If enantioselectivity data are available, I would display all of them in the phylogeny rather than only the three ancestors currently shown.

Reviewer #2

(Remarks to the Author)
The authors have addressed my concerns.

Reviewer #3

(Remarks to the Author)
The the paper by Zuson et al addresses the evolutionary origin of the enantioselectivity of plant dehydrogenases plants toward 1-borneol. The authors have performed an impressive work, combining ancestral sequence reconstruction, expression and characterization of relevant proteins, as well as state of the art simulations, shedding light on this interesting issue.
In the revised version, the authors have complied with the suggestions and concerns raised in my previous review related to the computational part of the paper. Moreover, the paper has been significantly improved also considering the changes made in response to the other reviewers. In my opinion, the paper is ready for publication in Nature Communications.

Reviewer #4

(Remarks to the Author)
The authors have very thoroughly and substantially responded to the majority of the points raised during review. The revised manuscript is much improved and significantly clearer. I do believe that the point raised about differences in some of the metrics for structure quality was not interpreted correctly (at no point were the structures suggested to be of insufficient quality - the suggestion was to address why these differences may have been observed). Having said that, the authors make a valid point in their rebuttal about the difference in space group and/or presence of ligands potentially causing the observed difference in some of the metrics (particularly B-factor and clashscore - there was a large difference between these metrics for the two structures, despite all of the other quality metrics being of similarly high quality). Assuming that the two structures were processed in the same way, it may be of value to briefly mention this explanation in the text, even if only in the methods section. Otherwise, the authors have done a great job at addressing all points.

Minor note - in the Supp Table 4 (page S47), the term "formiat" should be corrected to "formate"

Open Access This Peer Review File is licensed under a Creative Commons Attribution 4.0 International License, which permits use, sharing, adaptation, distribution and reproduction in any medium or format, as long as you give appropriate credit to the original author(s) and the source, provide a link to the Creative Commons license, and indicate if changes were made.
In cases where reviewers are anonymous, credit should be given to 'Anonymous Referee' and the source.
The images or other third party material in this Peer Review File are included in the article’s Creative Commons license, unless indicated otherwise in a credit line to the material. If material is not included in the article’s Creative Commons license and your intended use is not permitted by statutory regulation or exceeds the permitted use, you will need to obtain permission directly from the copyright holder.
To view a copy of this license, visit https://creativecommons.org/licenses/by/4.0/

We thank the referees for their time and effort and for their encouraging evaluation of the manuscript. Please find a point-by-point answer to the comments below.

Reviewer #1:

How enantioselective enzymes have evolved from nonspecific precursors is an important question that has never been directly addressed by ancestral reconstruction. The authors have developed an ideal model system and sophisticated biochemical and structural methods for this goal. To bring to them bear on an evolutionary question, however, a robust phylogenetic framework is equally important. The authors provide only a scant description of phylogenetics, making it impossible for me to evaluate the plausibility of their evolutionary inferences. To justify their reconstructed evolutionary history, the following questions must be answered at minimum:

Author response: Thank you for your insightful comments and feedback regarding the robustness of our phylogenetic inference. The main concerns centered around the models of sequence evolution (and how one was chosen), the confidence of our phylogeny and ASR, and the rooting of our tree. We have addressed these point-by-point below.

1. What exact model of sequence evolution was used, and how was it chosen?

The method section only says that the software PhyML was used for phylogenetics and the model JTT for ancestral reconstruction. I am sure there were more model features involved, such as the gamma-distributed among-site rate variation model and the invariant sites model; leaving these out typically results in a substantial model misfit. What was the exact model used? Why was JTT chosen among the many available substitution matrices? What statistical tests were performed to choose the best overall model of evolution?

Author response: In terms of evolutionary model analyzed, we chose WAG + G (4 gamma categories) for PhyML to conduct phylogenetic analysis to search tree space and then JTT + G using GRASP to infer ancestral sequences. PhyML tests different models and the model that fit best the data was selected. PhyML employs Smart Model Selection (SMS) to determine the most adequate model for the input data. In our experience, ancestral inferences are not overly influenced by models similar as WAG, JTT, LG, etc.

2. How well were the phylogeny and ancestral sequences reconstructed?

The confidence of neither phylogenetic inference nor ancestral reconstruction is described. What are the statistical supports for the branching orders? Are they congruent with the established taxonomies? What are the posterior probabilities for the reconstructed ancestral sequences? Are the paper's major conclusions robust to alternate possible reconstructions?

Author response: Phylogenetic nodal support is estimated by the approximate likelihood-ratio test (aLRT). The aLRTs of our nodes of interest N6, N30, and N32 is (0.95), (0.94), and (0.81) respectively. The joint posterior probabilities of the reconstructed ancestors are N6 (0.95), N30 (0.94), and N32 (0.88). The phylogeny is generally congruent with species taxonomy except for lineage specific gene duplication events that occurred during deeper evolutionary divergences.

3. How was the phylogeny rooted?

Incorrect rooting can lead to false inferences reversing the order of actual events. In the authors' phylogeny, for example, placing the root within the clade originating from N32 would flip the order of evolution—nonselective descendants evolving from selective ancestors. Rooting is not trivial for multicellular organisms when there are many paralogs as in the authors' case. The choice of the root must therefore be explained.

Author response: The phylogeny was rooted with two bacterial *Pseudomonas* sp. BDHs. As bacteria do not belong to the plant kingdom, they can be used as an outgroup to root the tree.

I regret that the excellent biochemical and structural data that the authors have collected cannot be used to make an evolutionary inference without first confirming that they are based on a robust phylogenetic inference.

Author response: We hope that our responses to the Reviewer's helpful comments have alleviated the Reviewers' concerns with regards to the robustness of our inference.

On a matter of presentation, a circular phylogeny with long taxon names (Fig. 2) is virtually unreadable. Please collapse the phylogeny into major clades and show their branch lengths and supports. The full tree can be shown in a supplementary figure along with more detailed information on ancestral reconstruction. A good example is this paper: [https://www.nature.com/articles/nature10724.](https://www.nature.com/articles/nature10724)

Author response: As requested by the Reviewer, we have changed the initial circular phylogenetic tree to a rectangular tree with collapsed clades (Figure 2 of the revised manuscript). We have further added an additional figure and included nodal support for the experimentally characterized ancestors (Supplementary Fig. 2). We anticipate that these changes increase the clarity of our phylogenetic results.

The following paragraph was revised:

"Eleven independent BLAST searches were carried out against the 1KP database[38,39], with BDH from *Lavandula intermedia* (AFV30207.1, *Li*BDH), alcohol dehydrogenase 2 from *Artemisia annua* (ADK56099.1 *Aa*ADH2), borneol dehydrogenase from *Artemisia annua* (ANJ65952.1, *Aa*BDH), borneol dehydrogenase from *Pseudomonas sp.* TCU-HL1 (WP_032492645.1, *Ps*BDH), borneol-type dehydrogenases 1 and 2 from *Salvia officinalis* (MT525100, *So*BDH1 and MT525099, *So*BDH2), borneol-type dehydrogenases 1 and 2 from *Salvia rosmarinus* (MT857224, *Sr*BDH1 and *Sr*BDH2) and borneol-type dehydrogenases 1 and 2 from *Tanacetum parthenium* (*Tp*BDH1 and *Tp*BDH2) as queries. Hits with an E-value lower than 10^{-5} were selected, and sequence duplicates were deleted and then aligned using ClustalW[36] and then modified using MUSCLE.[72] In pairs of sequences displaying a p-distance below 0.25, one sequence was randomly deleted. The remaining sequences were then realigned using ProbCons[73] and curated using Gblocks[36,37]. The phylogenetic tree was constructed using PhyML[74], and the ancestral sequence reconstruction based on the evolutionary model Jones-Taylor-Thornton (*JTT*) was carried out by GRASP[75]. PhyML uses Smart Model Selection to select the best fitting amino acid replacement model and uses the approximate Likelihood Ratio Test (aLRT) to calculate branch/node support."

The following figures were introduced to the manuscript and supplementary information:

a

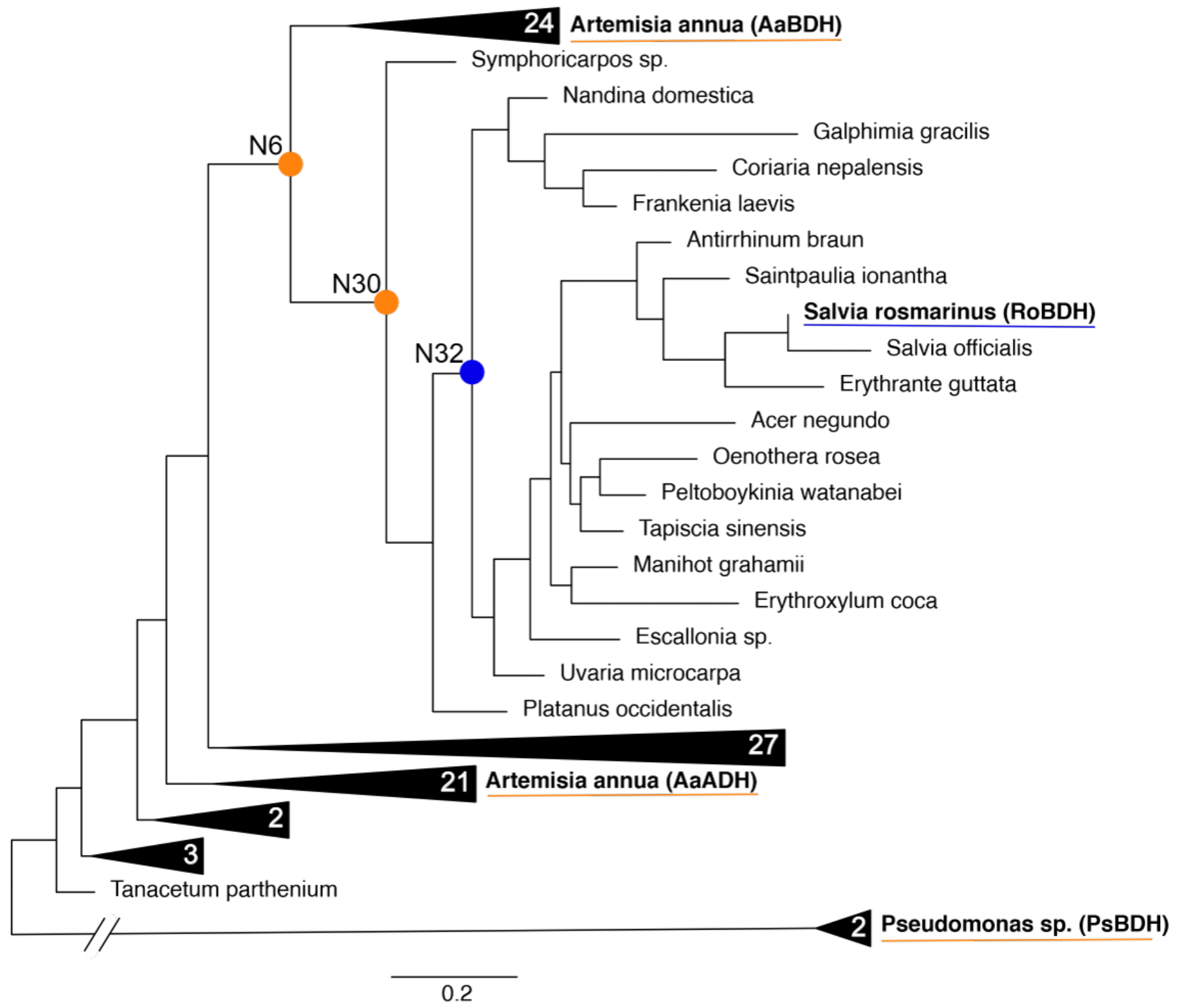

b

Enzyme	Specific activity[1] [mU/mg]		E_{comp}[2]
	(+)-1-borneol	(-)-1-borneol	
*Sr*BDH1	296.90 ± 15.30	1.30 ± 0.09	>200
N6	65.70 ± 8.54	0.74 ± 0.10	10
N30	34.10 ± 1.80	2.87 ± 0.35	12
N32	24.50 ± 1.46	n.c.[3]	>200

Fig. 1. (A) Phylogenetic tree of plant BDHs. Circles at nodes represent ancestral proteins that highlight the transition from unselective to selective enzymes. Unselective BDHs are represented in orange, while selective BDHs are dark blue. The number inside the triangles represent the total number of sequences in that specific node. The aLRT of nodes N6, N30, and N32 as calculated by PhyML was 0.95, 0.94, and 0.81, respectively. The posterior probability of nodes N6, N30, and N32 are calculated by GRASP as 0.96, 0.94, and 0.88, respectively. Scale bar represents 0.2 amino acid replacements per site per unit evolutionary time (branch leading to the outgroup is truncated). (B) Specific activity and enantioselectivity E_{comp}-values determined for *Sr*BDH1 and ancestors N6, N30 and N32 by oxidation of (+/−)-1-borneol enantiomers in comparison to the competitive oxidation between (+/−)-1-borneol and the reference compound. [1] determined by GC; [2] determined as the ratio of specific activities of an enantiomer over the rate of a competing co-substrate divided by the rate of the other enantiomer over the competing co-substrate (**Supplementary Equation 1**); [3] n.c.: no conversion.

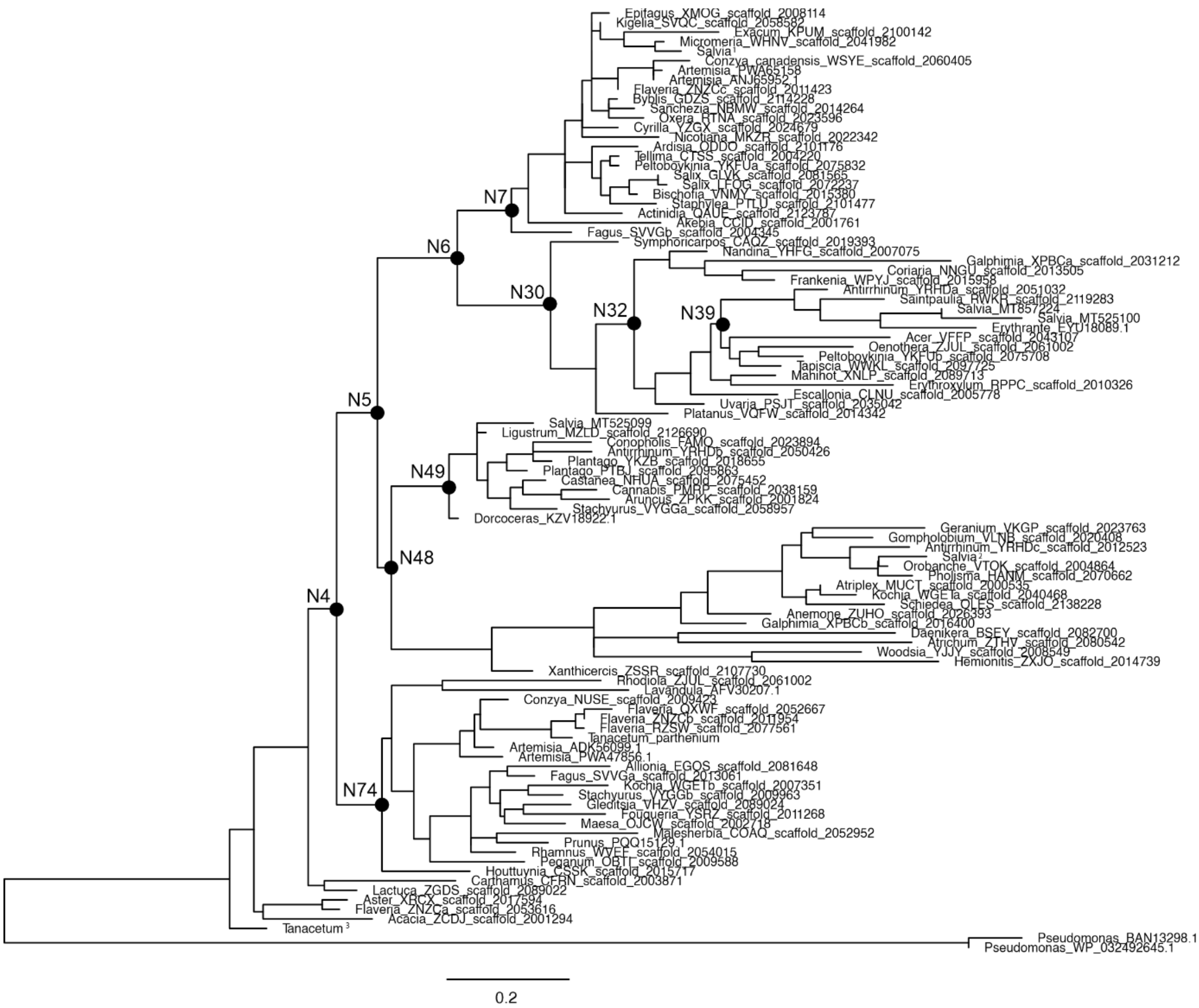

Supplementary Fig. 1. Phylogenetic tree of plant BDHs. The ten highlighted ancestors were characterized computationally or biochemically, or both. Salvia BDH sequences were reported by Chánique et. al. (2021), Tanacetum BDHs are reported under https://db.cngb.org/onekp/species/Tanacetum%20parthenium; sample code = DUQG.

Reviewer #2:

Zuson et al. investigated the evolutionary emergence of enantioselectivity in plant borneol dehydrogenases (BDHs) and identified that one mutation in the active site, along with three peripheral mutations, was required to achieve high selectivity, as confirmed by site-directed mutagenesis. They further employed molecular simulations for mechanistic studies. These findings advance our understanding of BDH enantioselectivity and may guide the rational design of more efficient biocatalysts.

I have a few comments and questions:

1. Back-mutation of the peripheral residues on N32 is suggested to further validate the contributions of these peripheral mutations.

Author response: In the manuscript, some of the mutants (N30_L111I/S166A/V167S/**G169A**, N30_L111I/**V136L**/S139G/**V183I**) combined the unselective N30 ancestor with the active site mutation (L111I) and one and two residues from the three indicated peripheral positions in N32 (L136, I183, A169); respectively, N30_L111I/V136L/G169A/V183I combined all three.

Following this suggestion, we back-mutated all three peripheral positions in the selective N32 without adjustments to the active-site and investigated the selectivity of this new variant (N32_L136V/A169G/I183V).

In the measurements, the N32_L136V/A169G/I183V variant still retains the high enantioselectivity displayed in N32. As such, no conversion of (-)-borneol was detected after 24 h at 9 mM substrate concentration and 20 µM of purified enzyme. This further confirms that I111 is essential for the enantio-discrimination in ancestor N32. This is further supported by mutant N32_I111L, as the selectivity is lost upon mutation. Seeing how in N30 the single mutation of L111I only moderately increases enantioselectivity, the results point out that N32 is further adapted to high enantioselectivity than N30. We would argue that the effect of first-shell residues is at least 1 C-atom (*i.e.*, 1.4-1.8 Å between Val and Ile); smaller changes can be achieved only by peripheral mutations that can change active site geometry to a smaller extent. The fact that backmutation of the three peripheral residues in N32, while retaining I111, did not increase activity and can be explained by the beneficial effect of the interaction of the other 15 residues that differ between N30 and N32. For instance, insertion of 6 of these residues into N30 (N30_L111I/L47W/E74K/V79I/E107A/E153Q/Y197H, table 1) increased selectivity.

We attempted to characterize the new variant N32_L136V/A169G/I183V kinetically, but determination of Michaelis-Menten kinetics proved more difficult than in the previously described variants, as the K_M is much higher, leading to a linear correlation of specific activity to molar concentration of the substrate within the boundaries of chemical solubility properties.

We have added an additional figure (Figure 6) to the manuscript, summarizing all mutations and backmutations between N30 and N32. The attempt to obtain kinetic parameters of N32_L136V/A169G/I183V was added to the supplementary information (Supplementary Figure 13 (bottom right)). To discuss the backmutation of the three peripheral residues, we added the following sentences to the manuscript:

"The peripheral mutations L136, I183, and A169 are also present in different combinations in previously described N30 variants (N30_L111I/S166A/V167S/G169A and N30_L111I/V136L/S139G/V183I). While the combined effect of these three conserved

residues results in high enantioselectivity, single or double mutations at these peripheral positions are ineffective, suggesting their synergy in N30. To investigate the role of these residues in the selective N32, the mutant N32_L136V/A169G/I183V was created. N32_L136V/A169G/I183V retained the high enantioselectivity observed in N32, further confirming that enantio-discrimination in ancestor N32 is primarily mediated by I111, which remains unchanged in this variant. The results also indicate that N32 is more strongly adapted to high enantioselectivity than N30. This property is likely stabilized by the combined effect of the remaining 15 residues that differ between N30 and N32. Consistent with this interpretation, high enantioselectivity in N30 can also be acquired by introducing a subset of these substitutions (N30_L111I/L47W/E74K/V79I/E107A/E153Q/Y197H, Table 1). Figure 6 summarizes the mutational strategies described above."

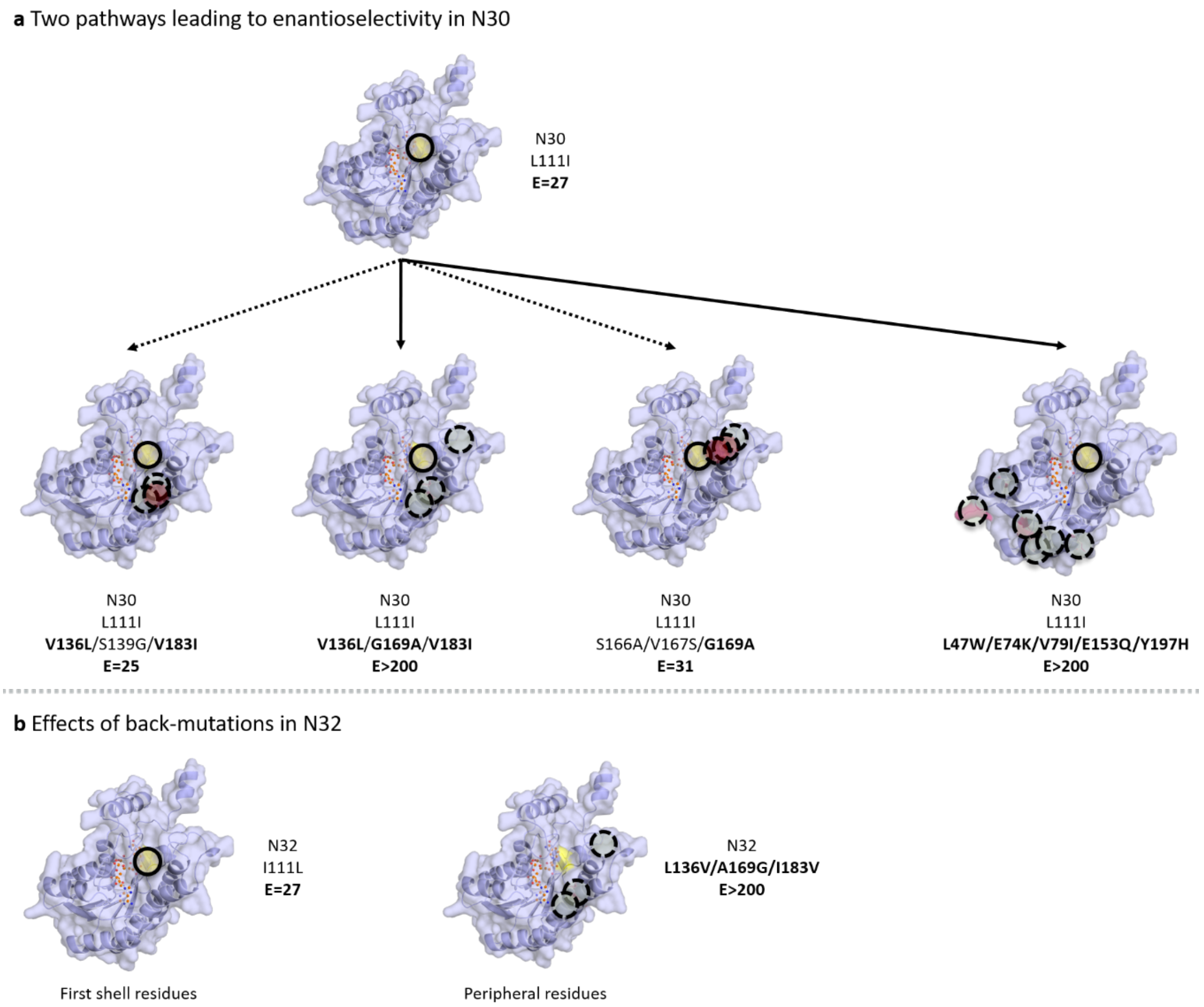

Fig.e 6. Overview of the changes in enantioselectivity between the N30 and N32 mutants. A) Two separate pathways leading to enantioselectivity in N30. B) Effects of back-mutations on N32 by incorporating residues from N30.

2. Kinetic data on all the mutants are needed to better elucidate how each mutation affects enantioselectivity.

Author response: Prompted by the Reviewer's comment, we determined the kinetics of three variants N32_ I111L, N30_L111I, N30_L111I/G169A/V136L/V183I with (+)-borneol, as (-)-borneol is not converted by N30_L111I and N30_L111I/G169A/V136L/V183I. In the case of

N32_I111L, activity towards (-)-borneol is exceedingly low (3 mU/mg). We tried to increase the enzyme concentration 10-fold, but the V_{max} is still too low for accurate kinetics.

We would like to respectfully direct the attention of the Reviewer to the fact that the combination of the very low substrate solubility in aqueous solution (max. 4 mM, increased to 9 mM by addition of 20% organic solvent), the high K_M-value of the variants, the very low activity towards (-)-borneol, and the limited possibility of increasing enzyme concentration due to precipitation, make comprehensive kinetic characterization challenging. Furthermore, 90 GC-measurements are required for the rate determination (n=3). In view of this considerable effort, we focused on the three variants that were deemed to be the most relevant. While limited by the solubility, we were able to obtain measurements where, at higher concentrations, the rate increase is no longer linear, which allows for estimation of the K_M-value by extrapolation. Importantly, the K_M of the unselective N30, the reconstituted selective variant N30_L111I/G169A/V136L/V183I, and the selective N32 are in the same range (12-20 mM), which indicates that the selectivity is based on k_{cat} and not on K_M.

The kinetic parameters were added to the manuscript, and the values are now reported as apparent K_M (K_{app}) to highlight the limitations of the method and acknowledge the non-saturating conditions. The K_{app} towards (+)-borneol of the variants does not differ substantially, pointing towards suppression of the activity towards (-)-borneol as the critical factor for selectivity. We have added corresponding text to the manuscript:

"The apparent kinetics of the key variants were assessed to determine whether differences in in enzyme kinetics contribute to the observed enantioselectivity (Supplementary Figure 13, Supplementary Table 1). The K_{app} was determined as 5.8 ± 2.5 mM for N32_I111L, 18.11 ± 5.0 mM for N30_L111I, and 11.86 ± 1.4 mM for N30_L111I/G169A/V136L/V183I. The K_{app} of N30_L111I is comparable to those of N30 and N32, indicating that the change in selectivity introduced by L111I is not driven by altered substrate affinity. In the case of N32_I111L and N30_L111I/G169A/V136L/V183I the K_{app} values are lower compared to N30 and N32. However, N30_L111I/G169A/V136L/V183I regains high stereoselectivity despite the reduced Kapp, further supporting the conclusion that substrate affinity alone does not determine enantioselectivity. In the case of N32_I111L this comparison is not concise. N32_I111L is a variant of N32 that has lost stereoselectivity; it displays similar specific activity to N30, but has a lower K_{app} than that of both variants. Only in this variant could the reduced K_{app} be associated with the detectable conversion of (−)-1-borneol. In the case of N30_L111I/V136L/G169A/V183I K_{app} could not be extrapolated from the datapoints indicating a high K_{app} while retaining enantioselectivity."

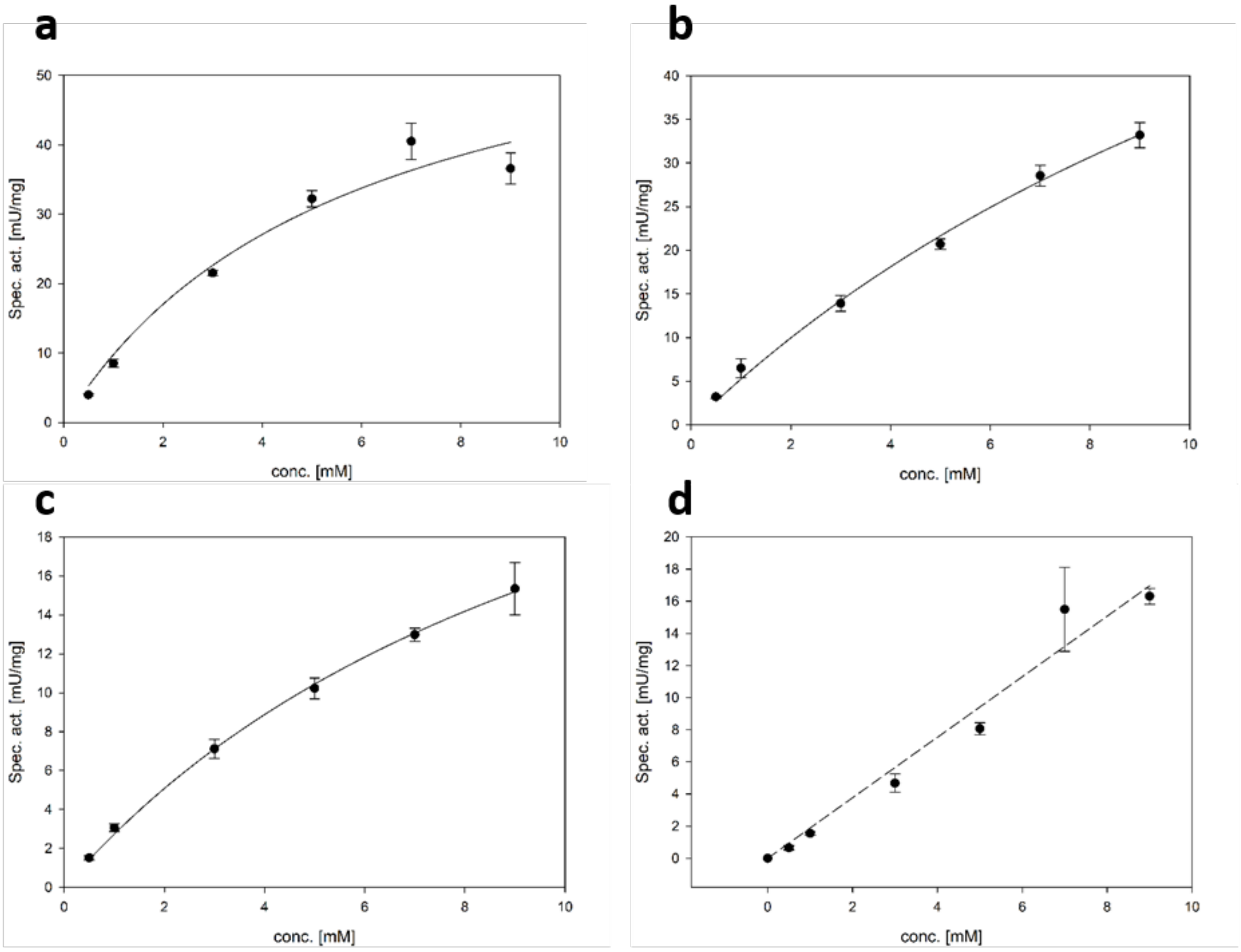

Supplementary Figure 13. Kinetic data fitted to the Michaelis-Menten model of (top left) N32_I111L, (top right) N30_ L111I and (bottom left) N30_ L111I/G169A/V136L/V183I, and N30_L111I/V136L/G169A/V183I (bottom right). Varying substrate conc. of (+)-borneol, 20% DMSO, 9 mM NAD^+, 20 µM purified enzyme, 0.1 M Tris-HCl pH = 9. Measured on GC-FID equipped with a ZB-5 column. (n=3). Raw data and GC-chromatograms of the measurement are deposited under 10.5281/zenodo.18925996 (Chromatogramms.zip; N32_I111L_kin.xlsx; N30_L111IG169AV136LV183I_kin.xlsx; and N30_L111I_kin.xlsx;).

***Supplementary Table 1**. Specific activity and K_{app}-values determined by gas chromatography for BDH variants by oxidation of (+)-1-borneol enantiomers.*

**Enzyme**	**Specific activity[I][mU/mg] (+)-1-borneol**	**K_{app} [mM]**
N32_I111L	36.6 ± 2.3	5.80 ± 2.5
N30_L111I	33.2 ± 1.4	18.11 ± 5.0
N30_L111I/G169A/V136L/V183I	15.3 ± 1.3	11.86 ± 1.4

3. The fonts in Figure 2A and 2B are too small to read.

Author response: Figure 2 has been replaced in accordance with the input of Reviewer 1, and font sizes for the whole figure have been increased.

4. In Table 1, the footnote cites [2], but this reference does not appear in the table.

Author response: We thank the reviewer for noticing this error. The footnote has been removed.

5. In the supplementary dataset, what do the numbers following the "_" in the protein names represent (e.g., what does the "0" in N2_0 indicate)?

Author response: This issue was also raised by Reviewer 1, please refer to our answer above (Question 2). In brief, the indicated values represent the joint posterior probabilities.

6. In Supplementary Figure 4, what do panels A, B, and C specifically represent?

Author response: A specification was added to the figure legend. A) represents N30, B) represents N32, and C) represents N6.

Reviewer #3:

The paper by Kourist et al presents results obtained using a variety of tools with the aim to shed light on the enantioselectivity of plant enzymes toward 1-borneol. The issue is interesting and the employed approaches showed to provide synergistic contributions. My review is focused on the computational part of the paper. I have discussed this review with a PhD student in my group, Andresa Messias (andresa@qi.fcen.uba.ar)

Comments/suggestions:

I wonder if the fact that the ligand bound simulations are unstable in all cases is due to a parametrization flaw. One way to shed light on this issue could be to perform QM-MM (taking the ligand as the QM region) simulations which may allow for ligand polarization. Since this may be prohibitive computationally, a possible strategy could be to perform multi scale machine learning - molecular mechanics (ML-MM) calculations that have been shown to achieve near QM-MM quality at a low computational cost. Two possible implementations for this that are easily available are:

a) Semelak JA et al, Advancing Multiscale Molecular Modeling with Machine Learning-Derived Electrostatics. J Chem Theory Comput. 2025 May 27;21(10):5194-5207. doi: 10.1021/acs.jctc.4c01792
AmberTools25 with the interface Torchani-AMBER: https://github.com/roitberg-group/torchani-amber
b) emle-engine: A Flexible Electrostatic Machine Learning Embedding Package for Multiscale Molecular Dynamics Simulations Zinovjev, K., et al Journal of Chemical Theory and Computation 2024 20 (11), 4514-4522 DOI: 10.1021/acs.jctc.4c00248
2) Binding free energies should be provided for the funnel meta dynamics simulations, in order to provide a more quantitative measurement of protein-ligand interactions. See for example Raniolo, S., Limongelli, V. Ligand binding free-energy calculations with funnel metadynamics. Nat Protoc 15, 2837–2866 (2020). https://doi.org/10.1038/s41596-020-0342-4.

Author response: We thank Reviewer #3 for their helpful comments, that prompted a substantial improvement of our computational protocol. Based on the combined feedback from Reviewers 3 and 4, we have extensively revised the structural modeling and free-energy strategy used in this work. The revised pipeline significantly strengthens the reliability of the conclusions, and now provides a unified mechanistic and framework for enantioselectivity.

At the beginning of this project, no experimental structure of the *Sr*BDH1 - borneol complex was available, and state-of-the-art deep-learning complex prediction tools were not yet accessible. For this reason, we initially employed docking-based modeling approaches. During the revision process, we adopted the new deep-learning structure-based complex prediction framework Chai-1, which has recently become available and has already proven highly reliable across multiple systems in our laboratory. Using Chai-1, we generated *Sr*BDH1 –(+) and (-) borneol complexes for all systems analyzed (WT, ancestors and variants). In all cases, (1) Borneol fits naturally into the active-site cavity without steric clashes and a clear induced-fit effect is observed in the catalytic pocket in compare with crystal structure. (2) Catalytic distances are perfectly preserved (d_1 and d_2), we now clarify what are those based on the measurements between the following heteroatoms: (d_1) the distance between borneol-oxygen and catalytic oxygen from the tyrosine, and (d_2) the distance between the oxidized borneol-carbon atom, and the reduced NAD^+ carbon atom. We include in our revision both figures and a new table from the new Chai1 models obtained (see Fig. 7 and Supplementary Table 7). This represents a major improvement over classical docking, and we now explicitly state in the manuscript that. We also highlight the potential of this methodology and might become an alternative of classical docking modeling strategy for systems lacking experimental complexes. Starting from the Chai-1 enzyme-substrate complexes, we applied two-approximations, one using classical molecular dynamics and the other one with ML/MM simulations using TorchANI-AMBER. Interestingly, unlike the MD simulations in our initial submission, the ML/MM simulations helpfully suggested by the Reviewer did indeed show a clear distinction in borneol selectivity and therefore we now focus our analysis in the main manuscript on the results from those simulations, with the conventional MD simulations moved to the SI for comparison.

In our revised workflow, we first perform a 100 ns restrained molecular dynamics simulation was performed in which harmonic positional restraints were applied to the substrate. This initial restrained phase was designed to enforce a stable coupling between *Sr*BDH1 and borneol, while allowing full structural relaxation of the protein environment, solvent reorganization, and rehydration of the catalytic pocket. After this equilibration period, all positional restraints were fully removed, and we ran either 1) an additional 400 ns of unbiased production molecular dynamics or 2) 10ns of ML/MM using TorchANI-AMBER (all simulations were performed in triplicate). Here we want to clarify that given the complexity of our models, with more than 1000 residues, our current velocity for ML/MM is 0.4ns/day, and also in reference DOI: 10.1021/acs.jctc.4c01792 they just ran 2ns of simulations given the computational cost; here, with just 10ns of simulation time, we were able to decipher enantioselectivity, which we could not do with 40x longer conventional MD simulations.

In the case of the conventional MD, we observed high instability of the complexes, whereas the ML/MM shows an improvement. With this ML/MM revised protocol, all complexes displayed significantly improved structural stability compared with our initial docking-based models, although the binding remained dynamically heterogeneous, in agreement with the experimentally observed moderate affinity and narrow selectivity window of borneol.

Finally, in response to the reviewer comments related to performing binding free energy calculations, we re-ran the free-energy calculations using our previously equilibrated structures. While the primary objective of the present study remains a mechanistic and qualitative description of enantioselective recognition rather than an absolute affinity ranking, the revised simulations now allow for a direct energetic comparison between systems and enantiomers. The resulting PMF profiles (**Supplementary Figures 33** to **45**) reveal that wild-type *Sr*BDH1 displays a narrow selectivity window between the (+) and (–) borneol enantiomers. This

selectivity progressively decreases when moving toward the reconstructed ancestral variants. Importantly, the wild-type enzyme and ancestor N6, which experimentally exhibit the lowest K_{app} values, also present the lowest PMF barriers among all systems analyzed. Conversely, the low-affinity mutants N32_I111L, N30_L111I, N30_L111I/G169A/V136L/V183I, and N32 display systematically higher PMF values. This establishes a clear quantitative correlation between the computed free-energy profiles and the experimental kinetic data. All PMF curves, numerical values, and their statistical analysis are now included in the revised manuscript in the form of new tables and dedicated discussion sections.

Reviewer #4:

Note: The review presented here focuses primarily on the structural and kinetic aspects of the reported work. I have intentionally omitted comments on the molecular dynamics experiments as I feel that I do not have the depth of expertise required to appropriately address the validity or potential implications of this element of the work.

In this manuscript the authors report on their attempt to reconstruct the evolutionary origins of enantioselectivity in borneol dehydrogenases. Guided by comparisons of sequence similarity and kinetic selectivity across species, they identify amino acids, most of which are outside of the active site, that play a putative role as the evolutionary switch-point for selectivity. Overall the way the data is presented is somewhat disconnected and, at times confusing. The relatively poor annotation and detail provided throughout, particularly in the figures, made it difficult to follow the narrative. While the applied principles appear to be sound and the data, as presented, seem to support the conclusions, the quality of some of the data, particularly the kinetics analysis, are questionable and raise questions about the interpretation.

Author response: We thank the referee for the encouraging assessment and constructive criticism. We have carefully revised the manuscript regarding experimental details and presentation, and Figure 6 has been added to further support the narrative.

Specific details of these criticisms follow.

Major points:
The data and analysis for the kinetics of the BDH-catalyzed reactions, upon which many of the manuscript's main observations and conclusions are founded, is of relatively poor quality, when presented at all. Despite having a rather extensive supplemental document accompanying the manuscript, raw data (or representatives of such) are not provided for the results shown in Table 1, beyond what is shown in Supp. Fig. 4.

Author response: We agree that transparent reporting of the data is crucial. For kinetics, we quantified product formation by GC in view of optimal data consistency. This means that 90 GC measurements were conducted for each kinetics analysis (n=3). For the sake of space, we did not share the original data. However, encouraged by the referee's comments, we deposited the GC-data of 3 kinetic analyses that are representative of all kinetic measurements and showed the initial rates. The initial rates were linear over the time-points measured by us. Therefore, we compared the kinetic parameters arising from taking different time points in order to assess the robustness of the v_0 measurements.

We have deposited raw data to Zenodo and added a link in the notes section of the manuscript. We highlight the corresponding files in the respective figure legends.

"Raw data of the biochemical analysis, structure predictions and simulation data have been deposited at Zenodo. The package contains chromatograms of three kinetic measurements, the extracted peak areas and calculations. The data is located at DOI: 10.5281/zenodo.18925996."

The curves provided in Supp. Fig. 4, if representative, suggests that the accuracy of the kinetics and specific activity data are poor. These data show incomplete curves that do not saturate and, as thus, are very difficult to fit accurately, particularly given that the error increases with increasing concentration. In fact, the data shown in A and B could likely be fit just as well with a linear model. This calls into question the actual reported values in Table 1 and, at the least, the reported precision. Since many of the decisions made in the experimental workflow stem from these activity-based assays, more thorough representation and analysis of the data, their quality and significance should be made available.

Author response: The referee raised an important issue with the accuracy of the kinetic measurements. We are confident that the product quantification with a flame ionisiation detector as basis of specific activity values has an acceptable precision. Biocatalysis experiments were repeated at least three times independently to determine the experimental error caused by extraction of the volatile and poorly soluble reactants after 24 h. Moreover, GC-MS is very sensitive and allowed us to rule out formation of (-)-camphor in measurements with the selective mutants. The experimental decisions were taken mostly on the finding that specific activity towards (-)-borneol drastically decreases, while the activity towards (+)-borneol is not significantly lower.

Regarding the determination of kinetic parameters, we agree with the referee about the limitations posed by the low solubility of borneol in aqueous solvents complicating enzymatic assays, which has been noted by others (doi: 10.1016/J.ABB.2012.09.013). This puts us effectively between a rock and a hard place in terms of our kinetic measurements. Specifically, the analysis of hydrophobic pockets necessitates the use of hydrophobic substrates. Solubility increase with addition of organic solvents worked only to a limited extent as the enzyme lost activity (Figure 5 in the main manuscript).

To achieve a more thorough representation of the data, we have uploaded the data of exemplary triplicate measurements to a repository and outlined the analysis. While not achieving saturation, most of the measurements exceed the linear range of the curve, which allows us to estimate on basis of a dynamic fit of the curve that the K_M-values of the mutants are not drastically altered. In only one case, the backmutation N32_L136V/A169G/I183V, the curve remained linear and no K_M-value was indicated. With the limited precision in mind, we indicated the K_M-values as apparent K_M-values (K_{app}) throughout the manuscript. To the best of our knowledge, there does not currently exist an experimental way how accurate kinetics of these variants with their high K_M-values and the poorly soluble substrate can be obtained.

We have stated this in a clearer way in the manuscript:

"We determined the Michaelis-Menten kinetics of the three ancestors towards the faster-converted (+)-1-borneol, which was complicated by the low solubility of the substrate in aqueous solution[43] and required extrapolation of data obtained below the K_M-value. Addition of 20% DMSO enhanced the solubility of borneol from 4.8 to 9.0 mM, while retaining around 40% of the overall enzymatic activity (Supplementary Fig. 4). Nevertheless, substrate saturation could not be reached, thus values are reported as apparent K_M-values (K_{app})."

- *The labeling of figures and references to figures in the text is inadequate and oftimes confusing. This made it difficult to follow the argument and identify key data in support. Some examples of this are provided here:*

  - *For the docking studies described on pp19-21, it was not clear what the authors were referring to as d1 and d2 in the figures or the table. Better description in the figure caption (Fig. 6) or labels on the figure itself, and some matching annotation on the table is needed to make this clear.*
    Author response: Thank you for pointing this out, we have now made sure the distances are clearly defined both in the figure caption (now Figure 8 after revisions), and in the main text.

  - *For figures 7 and 8, the figure caption refers to panels (B) and (C),butonly one panel (A) is labeled. I assume that the panels are read left to right, but this should be explicitly labeled*
    Author response: The figures have been exchanged and the label adjusted according to the reviewers input.

  - *Labels are needed in figures throughout the Supp. Figures document – for example, for Supp. Fig. 4 it is not described anywhere in the text or caption, which data corresponds to which enzyme or mutant – there are three panels, but no information in the figure caption describing what each is; it is unclear from the caption or the figures in Supp. Fig. 5, which peaks correspond to specific substrates or products*
    Author response: The issues raised by the reviewer were addressed by incorporating the respective enzymes in the figure legend of Supp. Figure 5 and the retention times of the respective peaks were indicated in the legend of Supp. Figure 6.

- While the structural data appear to be sound, the data could be represented more clearly and there are some questions that should be more fully addressed, specifically:

- *There is a distinct difference between the data reported for 8R0C and 8R0D,as evidenced by the substantially different B-factors, the much poorer crystallographic metrics for the former, and the poor clashscore/high number of RSRZ outliers for 8R0C. While these disparities may be justified by differences in data quality or structural features, this is not discussed in the manuscript. Some brief mention of what drives these differences would go a long way to assuage concerns about the structural data.*

Author Response: We respectfully disagree with reviewer, that our diffraction data and refined models are of insufficient quality. In Supplementary Table 4, we report several quality values, such as $CC_{1/2}$ (Karplus & Diederichs, Curr Opin Struct Biol 2015). For $CC_{1/2}$ we chose a "conservative" resolution cut off with ~ 0.5. Re-inspecting our XDS processing files, we can support the quality of our diffraction data with the Isa (theoretical value of $\langle I/\sigma\rangle_{ind}$ for an infinitely strong observation of the dataset calculated from coefficients of error model established during scaling. Isa gives insight into the level of fractional error in the dataset values): 8R0C with an Isa=26.0 as well as for 8R0C an Isa=31.5.

Further, the model quality as assed by Molprobity reported in Supplementary Table 4 indicates no problems in our structural models. For both structures we can report low clash scores and

very good Molprobity scores. Moreover, we calculated CC_{work} (model–data correlation): For 8R0C with a CC_{work}=0.87 as well as for 8R0C an CC_{work}=0.89.
Moreover, the reviewer mentions the RSRZ outliers for both structures.

8R0C:

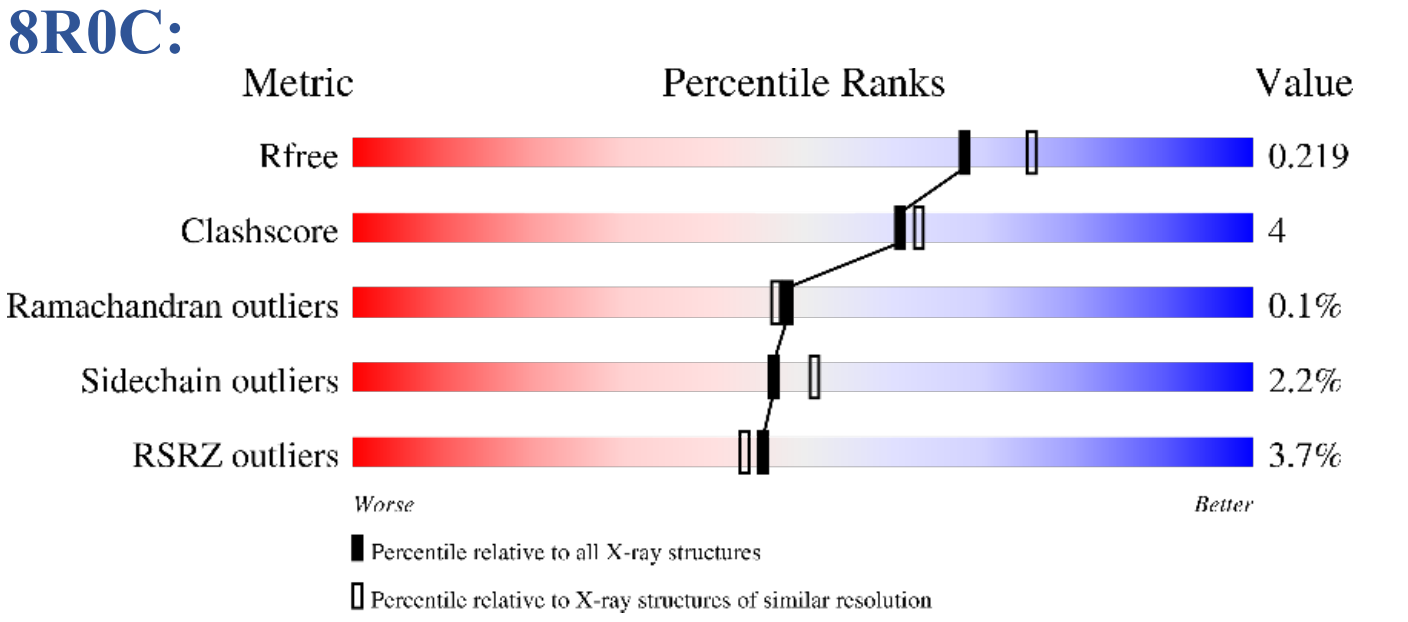

8R0D:

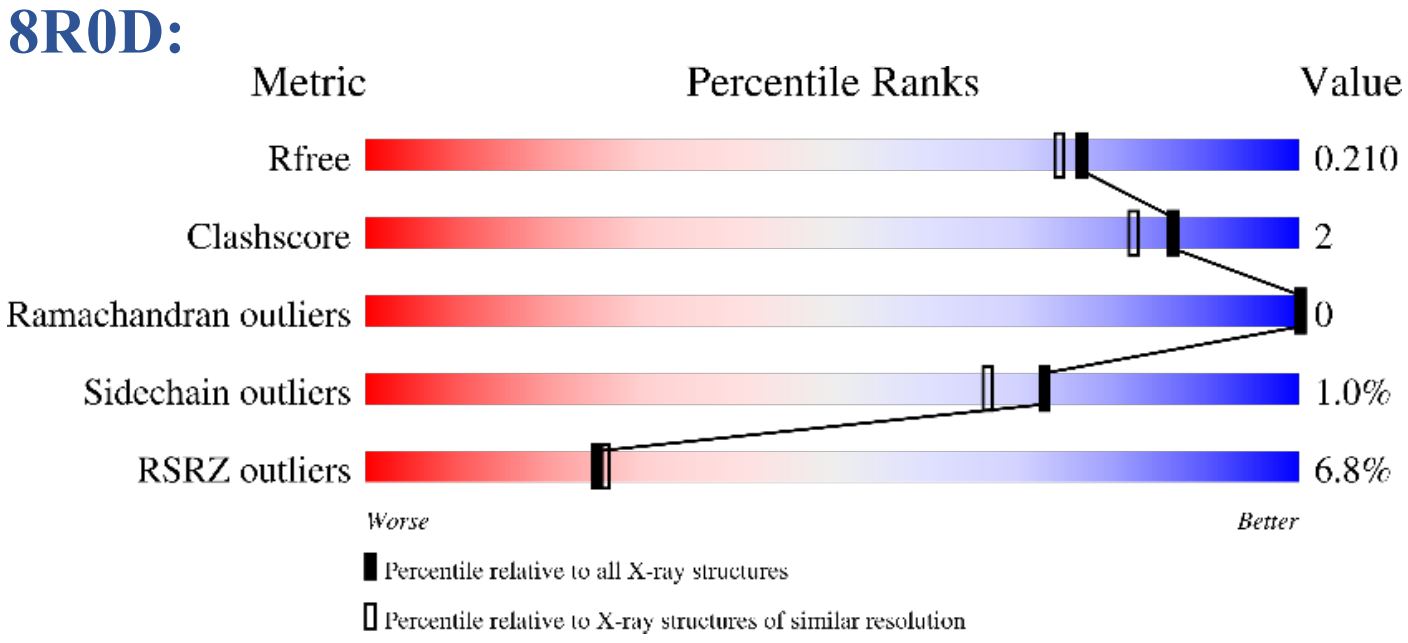

Our structures compare very well to both the percentile relative to all X-ray structures, deposited in the PBD, as well as the percentile relative to X-ray structures in the same resolution regime. We note that given the similar resolution of both structures, a direct comparison of both is not possible due to several facts like crystal symmetry, protein packing, presence or absence of low molecular weight compounds etc.

- *The way the structures are presented in Fig.4 does not clearly get the point across about the architecture of the active site. While attempts to get a liganded structure failed, it would help substantially to have some marker for the substrate or product position in these figures, even if it was a representation of the docked substrate as presented later in the manuscript. This would enable a clearer analysis of the features of the binding site. In addition, while the superpositions shown are useful, it would be helpful to show both a representation of the active site of each of SoBDH1 and SrBDH1 alone, preferably side-by-side. As additional minor points – it is not necessary to restate multiple times that "residues lining the active site are mostly hydrophobic" throughout the figure caption; it would be better to keep the case constant in the figure and the caption (currently the figure uses lower case letters and are referred to in the figure caption using upper case letters)*

Author response: We have re-drawn Figure 4 and rephrased the figure caption as suggested by the reviewer. The active site is schematically indicated in all four panels.

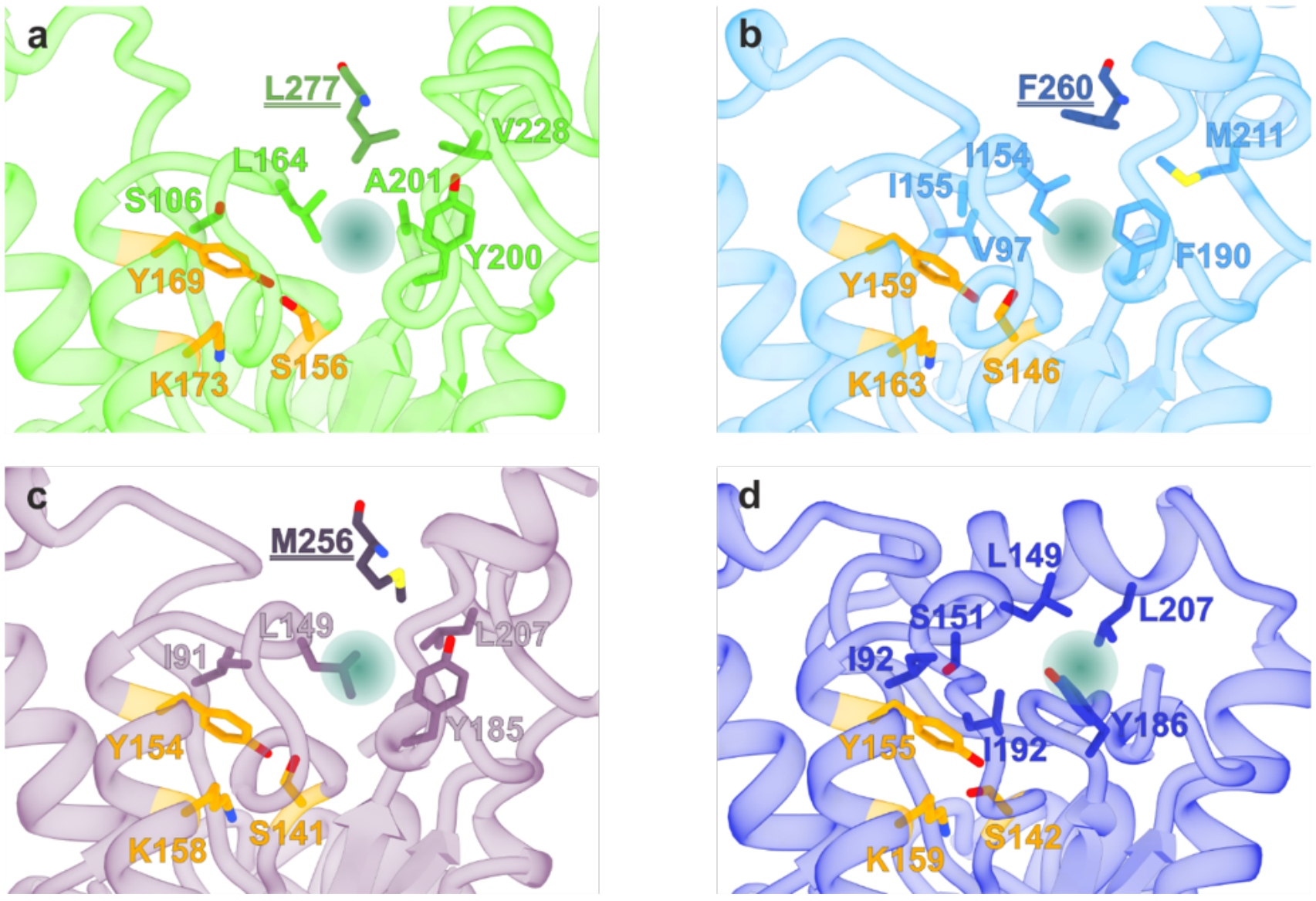

Fig. 4: Comparison of the active sites of **(a)** *Sr*BDH1 (PDB: 6ZYZ), **(b)** *So*BDH2 (PDB: 7o6P), **(c)** N32, and **(d)** N39. Catalytic residues are represented in orange. Residues originating from the adjacent protomer within the tetramer are underlined and represented in a darker shade, except for N39, where only chain A was resolved. Most residues lining the 4 active sites are hydrophobic. The teal-colored sphere represents the potential substrate binding-site.

- *On page 13, some points inferred from structural analyses could be clarified in the text, in some cases with the addition of figures or references. The authors describe differences observed in the loop αFG motif between chains – perhaps this could be shown with a figure in the supplement?*

Author response: Now, we show an additional figure in the Supplemental information (Fig. S4) with a superposition of the experimental and modelled BDH structures.

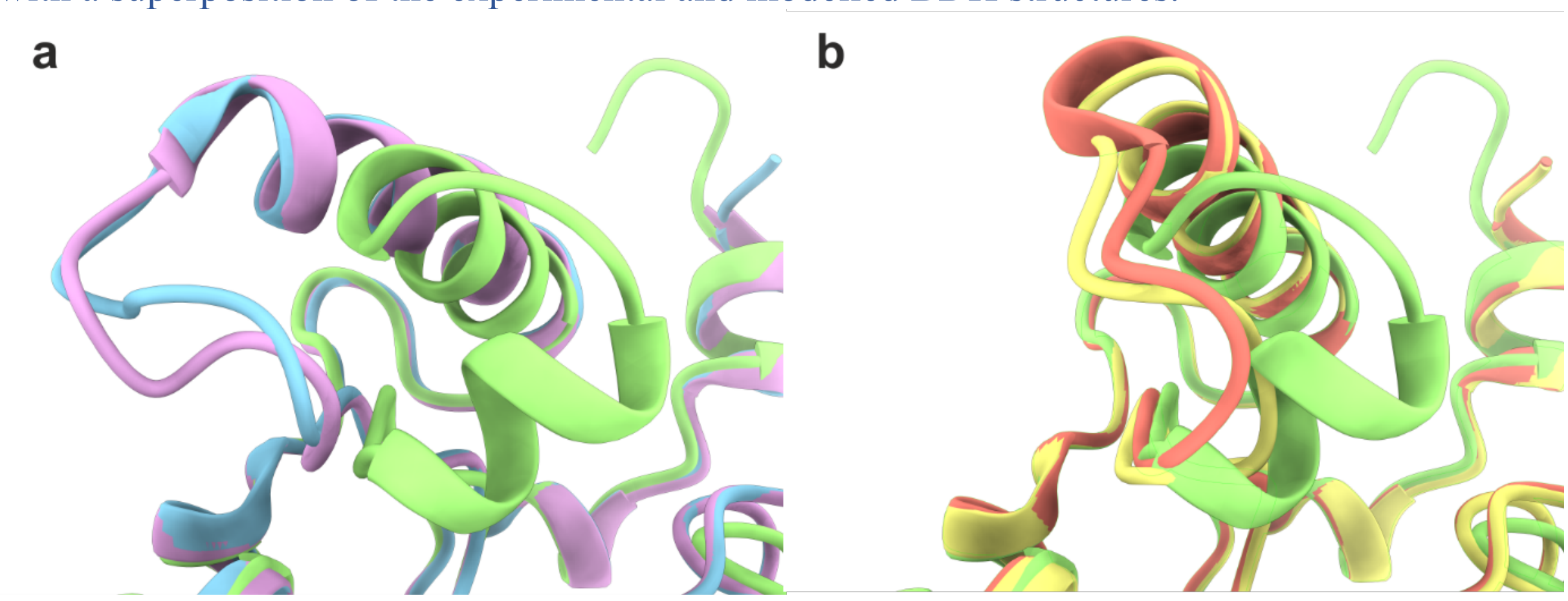

Supplementary Fig. 9: Comparison of the loop-αFG motif structure in the N6 AlphaFold3 model and in the 4 chains of *Sr*BDH1 (PDB: 6ZYZ). **a** Chains A (blue) and B (lilac) show an open conformation of the loop, differing from the predicted N6 model. **b** Chains C (yellow) and D (salmon), while not completely aligning with the predicted model, show a closed conformation, closer to N6.

On page 13, we have corrected the wording as highlighted below. Unfortunately, we had incorrectly mentioned the protein chains.

"As no high-resolution crystal structure of N6 was obtained, AlphaFold3 was used to generate a model, which showed a mean pLDDT of 89.4 with the lowest scores located at the termini (Supplementary Fig. 8). Despite an overall high similarity with *Sr*BDH1, differences can be observed in the loop-αFG motif of chain A and B, whereas chains C and D have a closed conformation, which aligns closer to the predicted AlphaFold3 model (Supplementary Fig. 9). Aligning the model of N6 to the crystal structure of *Sr*BDH1 yields an RMSD of 0.308 (Supplementary Table 5). Since the open confirmation appears to be an artefact unique to chain A of PDB: 6ZYZ, the generated models of non-crystallized variants were used for structural analysis."

It is not clear, in this context, what is meant by the statement "the generated models of non-crystallized variants were used for structural analysis" – Is this statement distinct from the prior discussion and made only to mention that the alphafold predictions are used in place of structures for those that did not crystallize, or does this refer somehow to which of the chains of the models predicted by alphafold were used in further analysis because of the observed difference between "open" and "closed" conformations?

- *It is not clear to me how accurate and appropriate the comparisons made based on the docking results (Table 2) are since these are comparisons between distances measured for one particular pose out of a docking run. Since these are docked structures, it is not immediately apparent that a 2 or 3 Å distance difference between systems is significant. It is stated that the "optimal" binding mode was determined by examining the distance between the 1-borneol O and the phenolic O of Tyr (and presumably taking that with the lowest value). This seems a rather limited basis for this selection. What about the binding geometry and other protein-ligand distances that may dominate the interaction? In addition, it would be more convincing if the docking was repeated for multiple starting poses and converged on a specific results and/or the "optimal" pose was further refined through MD or other means that considered flexibility of the ligand and protein.*

Author response: We thank the reviewer for these detailed and constructive comments (also coming from reviewer 3) regarding the modelling of *Sr*BDH1, which have led to substantial improvements in the clarity and quality of the structural and kinetic data presentation. The ambiguity concerning the docking distances d_1 and d_2 has now been addressed by explicitly defining these metrics in both Figures 6 & 7, and in Supplementary Table 7. We now state clearly that the distances correspond to the hydrogen-bonding interaction between borneol and the conserved catalytic tyrosine (d_1) and, and to the catalytic hydride transfer distance between borneol and NAD^+ (d_2). We further replaced the original docking-based representations with deep-learning-based enzyme–substrate complexes obtained using Chai-1. These Chai-1 models now provide an explicit and catalytically competent positioning of borneol within the active site for all systems. In all cases, borneol fits naturally into the active-site cavity, and displays a clear induced-fit effect, with a perfect match in catalytic distances to both the NAD^+ cofactor and the conserved tyrosine. These complexes are now used consistently throughout the revised manuscript as the structural reference for all subsequent conventional MD simulations, MM/ML and free-energy (metadynamics) simulations.

Reviewer #1 (Remarks to the Author):

I thank the authors for addressing all of the concerns I had raised in the initial review. I offer just two suggestions regarding the phylogeny on Fig. 2; the authors are free to incorporate or ignore them, and I ask for no further responses.

We thank the reviewer for their time and effort.

- *First, I would explain in the main text that the phylogeny is rooted by taking the two bacterial genes as outgroups.*

A sentence was added to highlight how the tree has been rooted.

- *Second, line 145 states that ten ancestral proteins were biochemically characterized. If enantioselectivity data are available, I would display all of them in the phylogeny rather than only the three ancestors currently shown.*

We have tried to add all the stereochemistry values, but it makes the figure very complex and very hard to understand. Therefore, Supplementary Table 2 was edited and E-values of the ten characterized ancestors were added to emphasize the transitions in selectivity throughout the investigated phylogeny.

Reviewer #2 (Remarks to the Author):

The authors have addressed my concerns.

We thank the reviewer for his time and efforts.

Reviewer #3 (Remarks to the Author):

The the paper by Zuson et al addresses the evolutionary origin of the enantioselectivity of plant dehydrogenases plants toward 1-borneol. The authors have performed an impressive work, combining ancestral sequence reconstruction, expression and characterization of relevant proteins, as well as state of the art simulations, shedding light on this interesting issue.

In the revised version, the authors have complied with the suggestions and concerns raised in my previous review related to the computational part of the paper. Moreover, the paper has been significantly improved also considering the changes made in response to the other reviewers. In my opinion, the paper is ready for publication in Nature Communications.

We thank the reviewer for his time and efforts.

Reviewer #4 (Remarks to the Author):

The authors have very thoroughly and substantially responded to the majority of the points raised during review. The revised manuscript is much improved and significantly clearer.

- *I do believe that the point raised about differences in some of the metrics for structure quality was not interpreted correctly (at no point were the structures suggested to be of insufficient quality - the suggestion was to address why these differences may have been observed). Having said that, the authors make a valid point in their rebuttal about the difference in space group and/or presence of ligands potentially causing the observed difference in some of the metrics (particularly B-factor and clashscore - there was a large difference between these metrics for the two structures, despite all of the*

other quality metrics being of similarly high quality). Assuming that the two structures were processed in the same way, it may be of value to briefly mention this explanation in the text, even if only in the methods section. Otherwise, the authors have done a great job at addressing all points.

The assumption of the reviewer is correct; both structures were processed in the same way. Both diffraction data sets were collected at 100 K with standard settings of the respective beamline. The diffraction data were both processed with XDS and scaled with XSCALE. Both crystal structures have been refined in PHENIX with essentially the same restraints and respective weights.

We have added the following paragraph to the Supplementary Material a new section entiteled "Supplementary Discussion". A link to the "Supplementary discussion" is provided in the "Methods" section of the manuscript.

"Despite identical processing of the diffraction data for SrBDH N32 and SrBDH N39, and refinement of both structures in PHENIX using essentially the same parameters, noticeable differences were observed in several refinement and validation statistics (Supplementary Table 4). Most prominently, the B-factors and clashscores differ substantially between the two structures.

To investigate the origin of these discrepancies, we examined the crystallographic properties of both datasets more closely. SrBDH N32 crystallized in the monoclinic space group $P2_1$, whereas SrBDH N39 crystallized in the orthorhombic space group *I*222, indicating a higher degree of crystal symmetry in the latter. The increased clashscore of SrBDH N32 is likely related not only to its slightly lower resolution, but primarily to the reduced symmetry and consequently lower number of crystal contacts. These differences in crystal packing may likewise account for the pronounced variation in the Wilson B-factors obtained from XDS.

Notably, the average B-factors of the refined models are consistent with the Wilson B-factors derived from the experimental diffraction data, arguing against over-refinement of either structure. Overall, the observed differences in refinement metrics are most plausibly explained by intrinsic differences in crystal properties, including crystal symmetry, molecular packing, thermal motion, and the presence or absence of low-molecular-weight compounds. Therefore, although both structures were determined at comparable resolution, a direct comparison of their refinement statistics is not straightforward."

- *Minor note - in the Supp Table 4 (page S47), the term "formiat" should be corrected to "formate"*

The two typos in Supplementary Table 4 have been corrected.

Review of Zuson et al. (NCOMMS-25-56314)

Note: The review presented here focuses primarily on the structural and kinetic aspects of the reported work. I have intentionally omitted comments on the molecular dynamics experiments as I feel that I do not have the depth of expertise required to appropriately address the validity or potential implications of this element of the work.

In this manuscript the authors report on their attempt to reconstruct the evolutionary origins of enantioselectivity in borneol dehydrogenases. Guided by comparisons of sequence similarity and kinetic selectivity across species, they identify amino acids, most of which are outside of the active site, that play a putative role as the evolutionary switch-point for selectivity. Overall the way the data is presented is somewhat disconnected and, at times confusing. The relatively poor annotation and detail provided throughout, particularly in the figures, made it difficult to follow the narrative. While the applied principles appear to be sound and the data, as presented, seem to support the conclusions, the quality of some of the data, particularly the kinetics analysis, are questionable and raise questions about the interpretation. Specific details of these criticisms follow.

Major points:

- The data and analysis for the kinetics of the BDH-catalyzed reactions, upon which many of the manuscript's main observations and conclusions are founded, is of relatively poor quality, when presented at all. Despite having a rather extensive supplemental document accompanying the manuscript, raw data (or representatives of such) are not provided for the results shown in Table 1, beyond what is shown in Supp. Fig. 4. The curves provided in Supp. Fig. 4, if representative, suggests that the accuracy of the kinetics and specific activity data are poor. These data show incomplete curves that do not saturate and, as thus, are very difficult to fit accurately, particularly given that the error increases with increasing concentration. In fact, the data shown in A and B could likely be fit just as well with a linear model. This calls into question the actual reported values in Table 1 and, at the least, the reported precision. Since many of the decisions made in the experimental workflow stem from these activity-based assays, more thorough representation and analysis of the data, their quality and significance should be made available.
- The labeling of figures and references to figures in the text is inadequate and oftimes confusing. This made it difficult to follow the argument and identify key data in support. Some examples of this are provided here:
    - For the docking studies described on pp 19-21, it was not clear what the authors were referring to as d_1 and d_2 in the figures or the table. Better description in the figure caption (Fig. 6) or labels on the figure itself, and some matching annotation on the table is needed to make this clear
    - For figures 7 and 8, the figure caption refers to panels (B) and (C), but only one panel (A) is labeled. I assume that the panels are read left to right, but this should be explicitly labeled
    - Labels are needed in figures throughout the Supp. Figures document – for example, for Supp. Fig. 4 it is not described anywhere in the text or caption, which data corresponds to which enzyme or mutant – there are three panels, but no information in the figure caption describing what each is; it is unclear from the caption or the figures in Supp. Fig. 5, which peaks correspond to specific substrates or products

- While the structural data appear to be sound, the data could be represented more clearly and there are some questions that should be more fully addressed, specifically:
    - There is a distinct difference between the data reported for 8R0C and 8R0D, as evidenced by the substantially different B-factors, the much poorer crystallographic metrics for the former, and the poor clashscore/high number of RSRZ outliers for 8R0C. While these disparities may be justified by differences in data quality or structural features, this is not discussed in the manuscript. Some brief mention of what drives these differences would go a long way to assuage concerns about the structural data.
    - The way the structures are presented in Fig. 4 does not clearly get the point across about the architecture of the active site. While attempts to get a liganded structure failed, it would help substantially to have some marker for the substrate or product position in these figures, even if it was a representation of the docked substrate as presented later in the manuscript. This would enable a clearer analysis of the features of the binding site. In addition, while the superpositions shown are useful, it would be helpful to show both a representation of the active site of each of SoBDH1 and SrBDH1 alone, preferably side-by-side. As additional minor points – it is not necessary to restate multiple times that "residues lining the active site are mostly hydrophobic" throughout the figure caption; it would be better to keep the case constant in the figure and the caption (currently the figure uses lower case letters and are referred to in the figure caption using upper case letters)
    - On page 13, some points inferred from structural analyses could be clarified in the text, in some cases with the addition of figures or references. The authors describe differences observed in the loop αFG motif between chains – perhaps this could be shown with a figure in the supplement? It is not clear, in this context, what is meant by the statement "the generated models of non-crystallized variants were used for structural analysis" – Is this statement distinct from the prior discussion and made only to mention that the alphafold predictions are used in place of structures for those that did not crystallize, or does this refer somehow to which of the chains of the models predicted by alphafold were used in further analysis because of the observed difference between "open" and "closed" conformations?
    - It is not clear to me how accurate and appropriate the comparisons made based on the docking results (Table 2) are since these are comparisons between distances measured for one particular pose out of a docking run. Since these are docked structures, it is not immediately apparent that a 2 or 3 Å distance difference between systems is significant. It is stated that the "optimal" binding mode was determined by examining the distance between the 1-borneol O and the phenolic O of Tyr (and presumably taking that with the lowest value). This seems a rather limited basis for this selection. What about the binding geometry and other protein-ligand distances that may dominate the interaction? In addition, it would be more convincing if the docking was repeated for multiple starting poses and converged on a specific results and/or the "optimal" pose was further refined through MD or other means that considered flexibility of the ligand and protein.
